# Tagging and Enriching Proteins Enables Cell-Specific Proteomics

**DOI:** 10.1016/j.chembiol.2016.05.018

**Published:** 2016-07-21

**Authors:** Thomas S. Elliott, Ambra Bianco, Fiona M. Townsley, Stephen D. Fried, Jason W. Chin

**Affiliations:** 1 Medical Research Council Laboratory of Molecular Biology, Francis Crick Avenue, Cambridge CB2 0QH, UK

## Abstract

Cell-specific proteomics in multicellular systems and whole animals is a promising approach to understand the differentiated functions of cells and tissues. Here, we extend our stochastic orthogonal recoding of translation (SORT) approach for the co-translational tagging of proteomes with a cyclopropene-containing amino acid in response to diverse codons in genetically targeted cells, and create a tetrazine-biotin probe containing a cleavable linker that offers a way to enrich and identify tagged proteins. We demonstrate that SORT with enrichment, SORT-E, efficiently recovers and enriches SORT tagged proteins and enables specific identification of enriched proteins via mass spectrometry, including low-abundance proteins. We show that tagging at distinct codons enriches overlapping, but distinct sets of proteins, suggesting that tagging at more than one codon enhances proteome coverage. Using SORT-E, we accomplish cell-specific proteomics in the fly. These results suggest that SORT-E will enable the definition of cell-specific proteomes in animals during development, disease progression, and learning and memory.

## Highlights

• A tetrazine-biotin probe containing a cleavable linker was created
• Proteomes labeled with cyclopropene amino acids were enriched and identified
• Proteome coverage is increased by targeting the amino acids to multiple codons
• Cell-specific proteomics was accomplished in the fly

## Teaser

Elliott et al. synthesize a cleavable tetrazine-biotin probe enabling identification of proteins that are tagged and enriched via stochastic orthogonal recoding of translation with enrichment (SORT-E). Proteome tagging at multiple codons increases coverage, and the authors demonstrate that SORT-E at several codons enables cell-specific proteomics in the fly.

## Introduction

Biomolecules, including proteins (Lang and Chin, 2014b, Ngo and Tirrell, 2011), DNA (Busskamp et al., 2014, Rieder and Luedtke, 2014), RNA (Asare-Okai et al., 2014), lipids (Yang et al., 2012), and sugars (Patterson et al., 2014, Prescher and Bertozzi, 2006), may be tagged with bio-orthogonal groups for many applications, including imaging, identification, and synthetic control. Global tagging of biomolecules may be achieved by feeding suitably tagged biosynthetic precursors to the natural biosynthetic machinery of the cell; an approach that requires the natural machinery to tolerate the introduction of the bio-orthogonal tag (Kiick et al., 2002). Analogs of natural precursors bearing bio-orthogonal tags may also be incorporated by engineered biosynthetic machineries with expanded substrate scope (Mahdavi et al., 2016, Ngo et al., 2013, Yuet et al., 2015). The creation of orthogonal biosynthetic machinery in cells enables the incorporation of diverse substrates without competition from endogenous substrates (Chin, 2014, Elliott et al., 2014a, Elliott et al., 2014b, Liu and Schultz, 2010). In addition to their use for tagging biomolecules, bio-orthogonal groups have also found extensive utility in a variety of other approaches, including as reactive handles in activity-based probes of protein function (Cravatt et al., 2008).

The majority of approaches to bio-orthogonal labeling reported to date take advantage of copper (I)-catalyzed reactions between azides and alkynes (Hong et al., 2009, Wang et al., 2003). These components are relatively small, and so metabolic precursors modified with azides and alkynes may be tolerated by the natural biosynthetic machinery of cells. However, certain azides are prone to reduction in the cellular milieu (Mancuso et al., 2013), and the reaction between azides and linear alkynes depends on copper (I) catalysis, which requires optimization to avoid damage to biomolecules (Hong et al., 2009). Strain-promoted reactions between azides and strained alkynes remove the copper dependence (Agard et al., 2004). Although many of these reactions are relatively slow, varying the groups appended to the azide or strained alkyne has led to increases in reaction rates. However, despite the substantial impact of azide-alkyne cycloadditions on biological discovery, additional approaches for the labeling of biomolecules and probes are required.

Recently, inverse electron-demand Diels-Alder reactions between strained alkenes, alkynes, and tetrazines have been recognized as excellent bio-orthogonal reactions (Blackman et al., 2008, Devaraj et al., 2008, Lang et al., 2012b, Patterson et al., 2012, Yang et al., 2012). These reactions can be exceedingly rapid (*k* ∼ 10^6^ M^−1^ s^−1^), proceed without catalysts, and produce nitrogen gas as the only by-product (Lang and Chin, 2014a). Alkenes and alkynes have been used to tag proteins, DNA, RNA, lipids, and sugars, enabling their labeling with tetrazine-fluorophore conjugates for imaging (Agarwal et al., 2015, Asare-Okai et al., 2014, Busskamp et al., 2014, Denk et al., 2014, Kurra et al., 2014, Lang et al., 2012a, Lang et al., 2012b, Nikic et al., 2014, Patterson et al., 2014, Pyka et al., 2014, Selvaraj et al., 2015, Uttamapinant et al., 2015, Yang et al., 2012). However, while azide-alkyne reactions form the basis of strategies to enrich tagged biomolecules for their identification by mass spectrometry (MS) (Bagert et al., 2016, Chesarino et al., 2014, Dieterich et al., 2006, Dieterich et al., 2007, Kleiner et al., 2015, Smeekens et al., 2015, Vanbeselaere et al., 2012, Zhang et al., 2013, Zheng et al., 2013), there are currently no methods for enriching and identifying biomolecules labeled with strained alkenes or alkynes through inverse electron-demand Diels-Alder reactions.

We, and others, have demonstrated that norbornene- (Kaya et al., 2012, Lang et al., 2012a, Plass et al., 2012), bicyclononyne- (Borrmann et al., 2012, Lang et al., 2012b), trans-cyclo-octene- (Lang et al., 2012b, Plass et al., 2012), and 1,3-disubstituted cyclopropene-containing (Elliott et al., 2014b) amino acids can be site-specifically incorporated into proteins in response to an amber codon introduced into a gene of interest using the pyrrolysyl-tRNA synthetase(PylRS)/tRNA_CUA_ pair and its active-site derivatives. This has enabled the imaging and control of protein function in vivo through the labeling of the tagged protein with appropriately functionalized tetrazine conjugates (Lang et al., 2012a, Lang et al., 2012b, Plass et al., 2012, Tsai et al., 2015, Uttamapinant et al., 2015).

**Figure 1:**
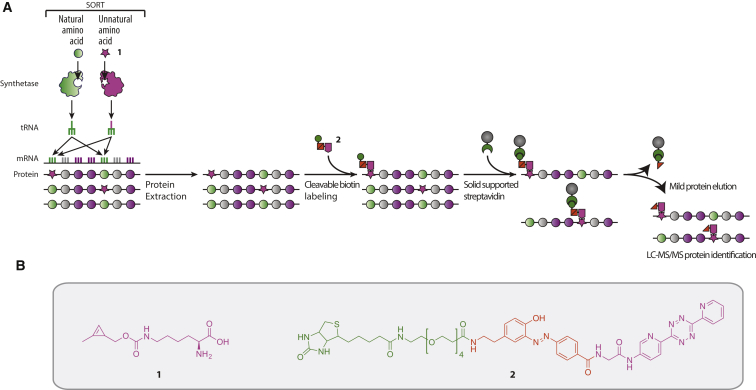
SORT-E Extends Proteome Tagging and Labeling to Protein Capture and Enrichment via Inverse Electron-Demand Diels-Alder Reactions with TDB, **2** (A) In SORT an unnatural amino acid (pink star) is recognized by an orthogonal pyrrolysyl-tRNA synthetase, and used to aminoacylate the cognate tRNA_XXX_ bearing a sense-decoding anticodon. This leads to the substoichiometric incorporation of the unnatural amino acid in response to the targeted sense codons. This approach has been implemented with the unnatural amino acid **1**. In SORT-E the proteins are extracted from cells and SORT-tagged proteins are captured with the tetrazine diazobenzene biotin compound, **2**, before capture on streptavidin beads. The beads are washed and enriched proteins are specifically eluted for detection by MS. (B) Structures of the unnatural amino acid *N*ɛ-(((2-methylcycloprop-2-en-1-yl)methoxy)carbonyl)-l-lysine, **1**, and the cleavable TDB (**2**), used in SORT-E.

An emerging application of bio-orthogonal labeling is for the imaging, and in some cases identification, of proteins expressed in particular cells at particular times in whole organisms (Elliott et al., 2014a, Elliott et al., 2014b, Erdmann et al., 2015, Yuet et al., 2015). We reported the first solution to this problem: stochastic orthogonal recoding of translation (SORT; Figure 1A) in which the CUA anticodon of tRNA_CUA_ is converted to a variety of triplets (XXX) that are complementary to diverse sense codons. This approach enables the incorporation of diverse amino acids in response to diverse sense codons (Elliott et al., 2014b). We demonstrated that **1** is a substrate for PylRS, and can be used for SORT (Elliott et al., 2014b). Because SORT uses an orthogonal synthetase and tRNA, there is no competition for the active site of the synthetase between **1** and natural substrates. Thus, SORT can be used to label newly synthesized proteins in cells and animals without the use of minimal media or starvation. Moreover, because the approach is genetically targeted it can be used to fluorescently label and identify newly synthesized proteins from specific cells, at specific developmental stages, within an animal via SORT with modification (SORT-M), in which **1** is labeled with tetrazine-fluorophore conjugates via an inverse electron-demand Diels-Alder reaction (Elliott et al., 2014b). We hypothesized that directing SORT to distinct codons by the use of tRNAs with distinct anticodons would lead to the labeling of different proteins with different efficiency, and that combining information on labeling at different codons may enable greater coverage of the proteome than labeling at any one codon (Elliott et al., 2014b).

Here we report SORT-E (SORT with enrichment) for the covalent capture and enrichment of SORT-labeled proteins via an inverse electron-demand Diels-Alder reaction with a tetrazine probe (Figure 1A). We demonstrate that SORT-E allows the substantial enrichment of proteomes tagged with **1** in response to diverse codons, and enables the identification and quantification of enriched proteins by MS (Bantscheff et al., 2012). We find that SORT-E does not preferentially identify proteins by molecular weight, but shows a slight bias toward the identification of low-abundance proteins, which should aid their identification. SORT-E at different codons leads to the enrichment of many proteins with different efficiencies, suggesting that proteome coverage may be increased by performing SORT experiments with several anticodon variants. To demonstrate the utility of SORT-E in a multicellular system, we create flies that tag newly synthesized proteins in ovary germ cells in response to several codons, and enrich and identify SORT-tagged proteins from these cells.

## Results

### Design and Synthesis of a Cleavable Tetrazine Diazobenzene Biotin Probe

We designed a probe, tetrazine diazobenzene biotin (TDB, **2**; Figure 1B), to enable the enrichment, purification, and identification of molecules labeled with strained alkenes or alkynes that undergo inverse electron-demand Diels-Alder reactions with tetrazines. The design of our probe builds on previous work developing and optimizing reagents for the covalent capture of proteins through azide-alkyne cycloadditions (Yang et al., 2010). Our probe has three components: (1) a tetrazine, (2) a biotin moiety, and (3) a cleavable diazobenzene linker between the tetrazine and biotin moieties (Szychowski et al., 2010, Verhelst et al., 2007) (Figure 1B). The 3,6-dipyridyl tetrazine is designed to facilitate rapid and selective inverse electron-demand Diels-Alder reactions of the probe with the tagged molecules in a sample. This group has good stability, and we previously reported that a TAMRA derivative of this tetrazine reacts with **1** at position 150 in GFP with a rate constant of 27 M^−1^ s^−1^ (Elliott et al., 2014b). While azide-strained alkyne cycloaddition reactions have been reported that are only 10-fold slower than this reaction (Dommerholt et al., 2014), these reactions use azides coupled to aromatic rings with electron-deficient substituents that cannot be incorporated into proteins for proteome labeling. The rate constants reported for the reactions between cyclo-octynes and azides, which are similar to those used for proteome labeling (Dommerholt et al., 2014), are 100- to 1,000-fold lower than the rate constant for the reaction of **1** in GFP with 3,6-dipyridyl tetrazines. The biotin moiety in TDB was designed to enable the capture of the labeled proteins on streptavidin-coated beads and facilitate the removal of unlabeled molecules in the sample by washing. The diazobenzene was designed to enable the selective release of labeled molecules, via reductive cleavage, for identification by MS.

**Figure 2:**
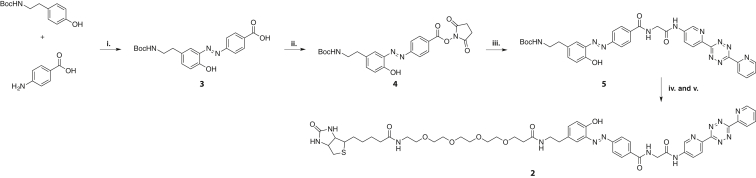
Synthesis of Compound **2** **i**. (a) 5 N HCl, NaNO_2_, 0°C, (b) NaHCO_3_, acetone, 0°C, 71% yield; **ii**. *N*-Hydroxysuccinimide, dimethylformamide (DMF), 1-ethyl-3-(3-dimethyl aminopropyl)carbodiimide, dimethylaminopyridine, room temperature, 48% yield; **iii**. Aminotetrazine, Et_3_N, DMF, room temperature, 28% yield; **iv**. trifluoroacetic acid, CH_2_Cl_2_, room temperature, quantitative yield; **v**. NHS-PEG_4_-Biotin (Thermo Scientific), DMF, Et_3_N, room temperature, 18% yield.

We developed a succinct synthesis of TDB probe **2** (Figure 2). Starting from two commercially available materials, a diazotization reaction afforded intermediate **3** in 71% yield. We found that direct coupling of carboxylate **3** with previously synthesized tetrazine amines (Lang et al., 2012a) could not be achieved using conventional carbodiimide or HATU chemistry. However, reacting a tetrazine amine with an activated ester, **4**, gave the protected diazobenzene **5** in 28% yield. Boc deprotection of **5** followed by coupling with an activated biotin conjugate gave the desired product, **2**, in 18% yield over two steps.

### Characterizing the Reaction of TDB with Proteins Incorporating **1**

**Figure 3:**
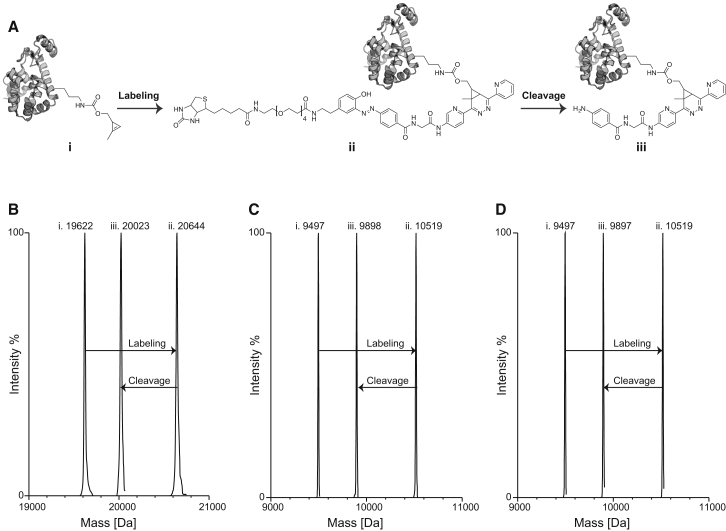
Quantitative Site-Specific Labeling of Genetically Encoded **1** with TDB, and Reductive Cleavage of the Diazobenzene by Mass Spectrometry (A) Proteins with site-specifically incorporated **1** (**I**) were labeled with 20 μM **2** at room temperature overnight to furnish (**II**). Treatment of (**II**) with 25 mM Na_2_S_2_O_4_ for 30 min generates the cleavage product (**III**). (B) Deconvoluted mass spectra for species (**I**), (**II**), and (**III**) for T4-lysozyme (K-83-**1**)-His6. (C) Deconvoluted mass spectra for species (**I**), (**II**), and (**III**) for ubiquitin (K-6-**1**)-His6. (D) Deconvoluted mass spectra for species (**I**), (**II**), and (**III**) for ubiquitin (K-48-**1**)-His6. For each protein, mass increases by 1,022 Da upon conjugation with **2** (as expected), and then decreases by 621 Da upon reductive cleavage of the biotin moiety (as expected).

Next we characterized (1) the labeling reaction between the probe and proteins in which we site-specifically incorporated **1**, and (2) the subsequent reductive cleavage of the linker by MS (Figure 3A). We produced T4 lysozyme (K-83-**1**)-His6, ubiquitin (K-6-**1**)-His6, and ubiquitin (K-48-**1**)-His6 from the corresponding genes (*T4 lysozyme (83TAG)-His6*, *Ubiquitin (6TAG)-His6*, and *Ubiquitin (48TAG)-His6*). Proteins were produced from *Escherichia coli* DH10B (T4 lysozyme) or BL21(DE3) (ubiquitin) expressing the PylRS/tRNA_CUA_ pair, which directs the incorporation of **1** in response to the amber codon, and purified by Ni-nitrilotriacetic acid chromatography with yields of 20–40 mg/l of culture. The incorporation of **1** in each protein was confirmed by electrospray ionization MS (ESI-MS) (Figures 3B–3D and S1).

We incubated each protein with 10 molar equivalents of **2** overnight at room temperature in 8 M guanidinium chloride. ESI-MS confirmed the quantitative labeling of each protein with **2** (Figures 3B–3D). Addition of Na_2_S_2_O_4_ (25 mM, room temperature) to the labeled protein led to quantitative reductive cleavage of the diazo bond within 30 min, as judged by ESI-MS (Figures 3B–3D). We further analyzed the reduced products by liquid chromatography-tandem MS (LC-MS/MS) to directly confirm the identity and position of the final protein modification (Figure S2). Taken together, these experiments demonstrate that we can quantitatively and specifically capture biomolecules bearing 1,3-disubstituted cyclopropenes using TDB, and that we can quantitatively and specifically cleave the probe to an amino benzamide once it is ligated to the biomolecule of interest.

### Enriching Labeled Proteins via SORT-E

Next we demonstrated that TDB (**2**) can be used to selectively enrich proteins that have been labeled with **1** via SORT. To label the proteomes of cells, we grew cultures containing one of four PylRS/tRNA_XXX_ pairs (where XXX = AGA, GCU, CAU, or UUU) in the presence of **1** (0.1 mM) (Elliott et al., 2014b). In control experiments we omitted **1** from the media. To confirm that **1** was incorporated into the proteome via SORT, we lysed the cells and treated a portion (240 μg) of each lysate with a tetrazine-fluorophore conjugate **6** (Figure S3) (Lang et al., 2012a). As expected, we see fluorescent labeling of the proteomes derived from cells grown in the presence of **1**, but almost no labeling of the proteomes derived from cells grown in the absence of **1** (Figure S3).

To enrich SORT-labeled proteomes, we labeled protein lysates (500 μl, at 8 mg/ml protein concentration) with **2** (20 μM, final concentration) at room temperature overnight. The specific and rapid reaction of **2** with 1,3-disubstituted cyclopropenes allowed us to use a relatively low concentration of **2**. Using a low concentration of **2** allowed us to add the streptavidin-coated beads (that have a high capacity, 10 mg biotinylated protein per 1 ml of settled resin) directly to the reaction mixture, as the capture of conjugates between proteins and **2** by the streptavidin beads is not limited by the binding of free **2** to the streptavidin beads. This is in contrast to other covalent enrichment approaches, which use much more labeling reagent in an effort to drive slower reactions to completion (equivalent azide reaction requires five times more linker, 1 mM CuSO_4_, 1 mM tris(2-carboxyethyl)phosphine, and an expensive ligand [Yang et al., 2010]) and precipitate proteins overnight to remove free label prior to capture on streptavidin. By omitting the precipitation step we simplify and accelerate the experiment, and by reducing the number of handling steps we potentially reduce protein loss and sources of experimental error. We incubated the labeled lysate with the beads (1.5 hr at room temperature) prior to washing.

**Figure 4:**
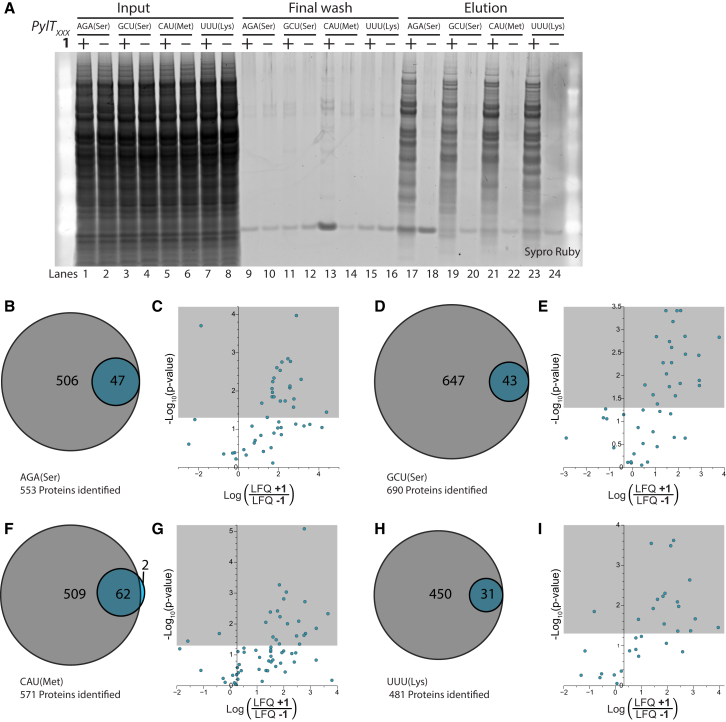
SORT-E Selectively Enriches Proteins Labeled with **1** (A) Cells expressing PylRS and either tRNA_AGA(Ser)_, tRNA_GCU(Ser)_, tRNA_CAU(Met)_, or tRNA_UUU(Lys)_ were grown either with or without **1** (0.1 mM). Lysates were labeled with **2** and tagged proteins captured with streptavidin beads, non-specifically bound proteins were washed away, and specifically captured proteins were eluted by cleavage of **2**. Aliquots from the initial lysate, the final wash, and eluted fraction were analyzed by SDS-PAGE. (B, D, F, and H) Venn diagrams representing the number of proteins identified by MS from the SORT-E elution (gray) and the no-amino-acid control (blue). (C, E, G, and I) Proteins that are identified in both the SORT-E samples (+**1**) and controls (–**1**) are significantly enriched in the +**1** samples. The volcano plots show the log ratio of the LFQ values for each protein in this subset (i.e., the enrichment factor), plotted against the log p value of the null hypothesis that there is no difference between the LFQ values. The area shaded in gray corresponds to the threshold of p values of <0.05.

We washed the beads extensively to remove non-specifically bound proteins. The final wash for all samples contained very little protein (Figure 4A), consistent with the effective removal of non-specifically bound proteins by the wash steps. Following the washes, we eluted proteins that were linked to the beads through the diazo group of **2** using PBS supplemented with 1% SDS and 25 mM Na_2_S_2_O_4_. Substantial amounts of protein from samples grown in the presence of **1** were eluted with Na_2_S_2_O_4_ while very little protein was eluted from samples grown in the absence of **1** (Figure 4A). We conclude that our procedure enables the specific capture of proteins labeled with **1**. Quantifying the protein in the GCU (Ser) elution (Figure S4) reveals that approximately 39 μg of protein was recovered. Since the input for the pulldown was 4 mg of protein, and SORT tags targeted proteins in response to target codons with an efficiency of substantially less than 1% (Elliott et al., 2014b), this enrichment step efficiently captures tagged proteins. We conclude that our approach, which we call stochastic orthogonal recoding of translation with enrichment (SORT-E), efficiently recovers and enriches SORT-labeled proteins. To define the limits of specific enrichment, we mixed SORT-labeled lysates with increasing amounts of unlabeled lysates. With a 10-fold excess of unlabeled lysate SORT-E still leads to selective enrichment, while for a 100-fold excess of unlabeled lysate non-specific labeling approaches the level of specific labeling (Figure S5). These data demonstrate that SORT-E provides a powerful approach for enriching proteins that are substoichiometrically tagged in target cells from a vast excess of untagged proteins.

### Identifying Enriched Proteins by Mass Spectrometry

To demonstrate that our approach enables the specific identification of proteins labeled with **1** by SORT and to further quantify the specificity of the approach for identifying labeled proteins, we performed in-gel tryptic digests and LC-MS/MS of the eluted proteins. For cells grown in the presence of **1** and the PylRS/tRNA_XXX_ pairs we identified 553, 690, 571, and 481 proteins (for XXX = AGA, GCU, CAU, and UUU, respectively) from approximately 1.3 μg of protein (i.e., using just 3.3% of our elution) (Figures 4B–4I and Table S1). The samples from cells grown in the absence of **1** yielded a small subset of the proteins identified from cells grown in the presence of **1**, with two exceptions for CAU. We identified 47, 43, 64, and 31 proteins from cells grown in the absence of **1** (for XXX = AGA, GCU, CAU, and UUU, respectively, Figures 4B–4I). Thus 92% (±2.2%) of proteins identified from cells grown in the presence of **1** are not found in cells grown in the absence of **1**. For the remaining 8% (±2.2%) of proteins that are captured for cells grown in both the presence and absence of **1**, we compared the abundance of each protein, as judged by label free quantification (LFQ) (Figures 4C, 4E, 4G, 4I, and S6) (Cox et al., 2014). The vast majority of proteins that are found in both samples, and for which we have statistically significant data, are enriched in the cells grown in the presence of **1** (log[LFQ + **1**/LFQ − **1**] > 0).

### SORT-E Enhances the Identification of Low-Abundance Proteins

**Figure 5:**
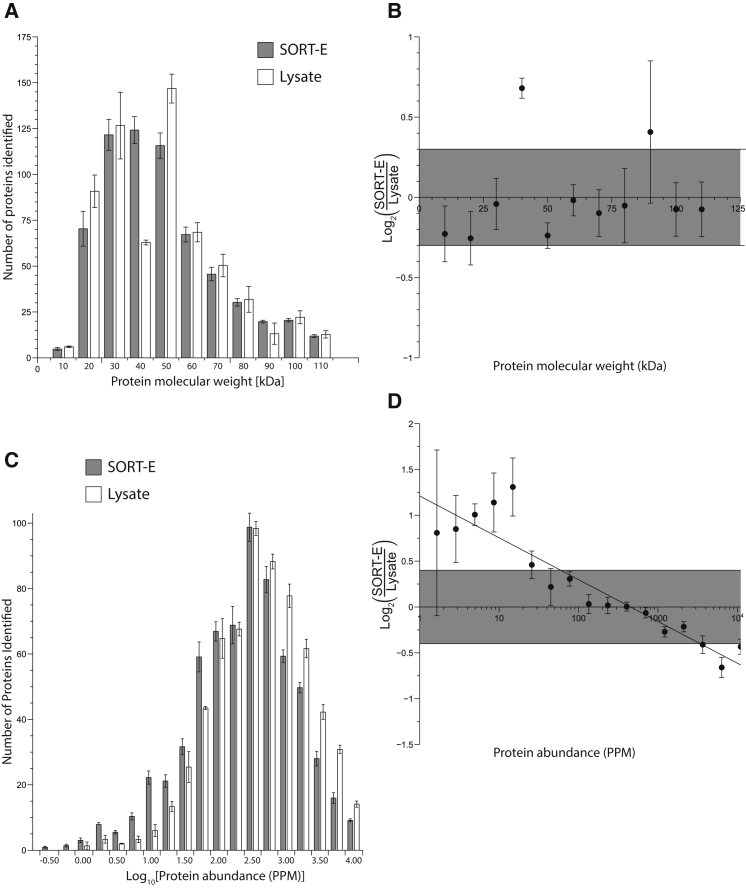
SORT-E Enriches Proteins of Most Molecular Weights Equally, but Preferentially Enriches Less Abundant Proteins from the Proteome (A) Number of proteins identified, either from crude lysates (white bars) or from SORT-E (gray bars). Data are binned by molecular weight. Error bars represent the SD of three biological replicates. (B) SORT-E identifies proteins across the full range of molecular weights of the *E. coli* proteome and shows no bias for larger or smaller proteins. The graph shows the log ratio of the number of proteins identified from SORT-E to the number of proteins identified from crude lysates, as a function of protein molecular weight, using the bins from (A). Error bars represent the SD of three biological replicates. Points that fall within the shaded area have a relative abundance of proteins before and after SORT-E that is within a factor of 1.32. (C) Number of proteins identified, either from crude lysates (white bars) or from SORT-E (gray bars). Data are binned by protein abundance in the PAX database of the *E. coli* proteome. Error bars represent the SD of three biological replicates. (D) SORT-E preferentially enriches low-abundance proteins. Log ratio of the number of proteins identified from SORT-E to the number of proteins identified from crude lysates, as a function of protein abundance defined in the PAX database. Error bars represent the SD of three biological replicates. Points that fall within the shaded area have a relative abundance of proteins before and after SORT-E that is within a factor of 1.32.

To address the extent to which our approach reproduces the distribution of proteins present in the proteome, we compared enriched proteomes identified via SORT-E with the proteome prior to TDB enrichment (Figure 5A). We find that the relative abundance of proteins for the vast majority of molecular weight ranges is comparable before and after TDB enrichment (Figures 5A and 5B). However, for the molecular weight range between 30 and 40 kDa we observed an increase in the number of proteins identified after the TDB enrichment.

To address the extent to which TDB enrichment captures protein present at different abundances in the cell, we compared the distribution of proteins before and after SORT-E as a function of abundance in the cell, using the data compiled in the PAX database (Figures 5C and 5D) (Wang et al., 2012, Wang et al., 2015). We found that the distribution of proteins following TDB enrichment (Figure 5C) is slightly shifted to favor low-abundance proteins, with respect to the distribution of proteins prior to enrichment. This observation was further supported by additional experiments (Figure S7). This basis of this enrichment is unclear, but it may result from the TDB enrichment decreasing sample complexity and bringing some low abundance proteins into a concentration range where they can be more easily detected by MS.

### SORT-E at Different Codons Enriches Different Proteins

**Figure 6:**
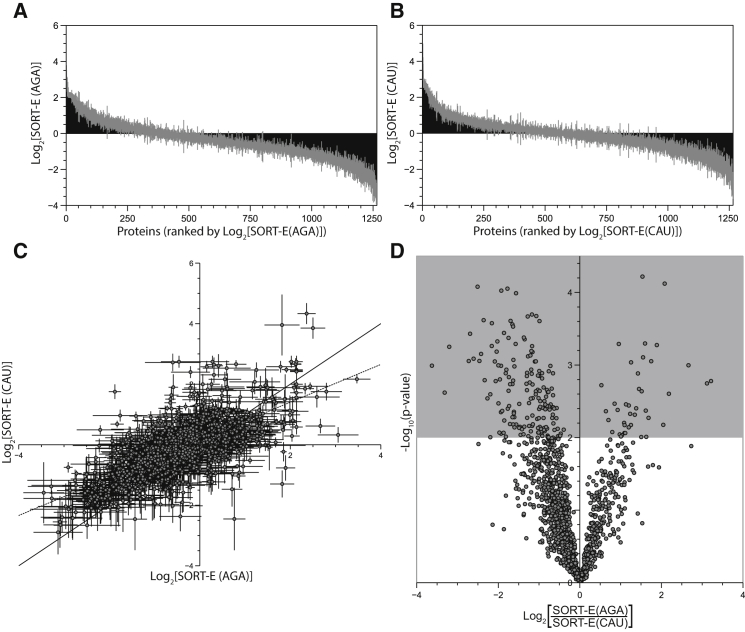
The Efficiency of Enrichment Depends on the Identity of the Anticodon used in SORT-E (A) SORT-E (AGA, Ser) enriches different proteins in the proteome to different extents. The graph shows the log of the ratio of the relative abundance for proteins detected for SORT-E to the relative abundance prior to enrichment. The data were measured in a multiplexed TMT experiment in which 1,265 proteins were common to all samples. The data are ranked by this ratio from most enriched to least enriched, to show the distribution of enrichments. Error bars, shown in gray, are the SD of three biological replicates each performed in duplicate. (B) As for (A), but for SORT-E (CAU, Met). (C) SORT-E (AGA, Ser) and SORT-E (CAU, Met) enrichments are globally correlated. The values in (A) and (B) were plotted to examine the correlation between protein enrichments in SORT experiments that use tRNAs with two distinct anticodons (AGA and CAU). The correlation (least-squares regression line y = 0.157 + 0.624x; R^2^ = 0.48, p = 10^−179^) between the enrichments for each codon is indicated by the dashed line. The solid line represents y = x, indicating what the best fit would look like if the enrichments were identical between the two anticodons. Error bars represent the st. dev. of three biological replicates. (D) SORT-E with distinct anticodons enriches a substantial subset of proteins with different efficiencies. The volcano plot shows the ratio of the data shown in (C), plotted against the p value of the null hypothesis that the protein is enriched with identical efficiencies for SORT-E (AGA, Ser) and SORT-E (CAU, Met). The 203 points in the upper shaded region correspond to the 16% of proteins that are significantly (p < 10^−2^) more enriched by SORT-E (AGA, Ser) (right branch) or SORT-E (CAU, Met) (left branch).

To investigate how the relative abundance of individual proteins is affected by the TDB pulldown using SORT systems addressed to different codons, we performed multiplexed tandem mass tagging (TMT) labeling and MS (McAlister et al., 2012, Thompson et al., 2003, Werner et al., 2012) (Figures 6 and S8; Table S2). For SORT-E (CAU, Met), in which **1** is stochastically incorporated in place of Met using a tRNA with a CAU anticodon, we observed proteins that increase as a fraction of total proteins after TDB enrichment, and proteins that are unchanged or depleted as a fraction of total proteins after TDB enrichment (Figure 6A). For SORT-E (AGA, Ser), in which **1** is stochastically incorporated in place of Ser using a tRNA with an AGA anticodon, we observed proteins that increase as a fraction of total proteins after TDB enrichment, and proteins that are unchanged or depleted as a fraction of total proteins after TDB enrichment (Figure 6B). We find that, broadly, there is a positive correlation in enrichment of proteins in SORT-E (CAU, Met) and SORT-E (AGA, Ser) (Figure 6C). However, there are clearly many proteins that are significantly and preferentially enriched by SORT-E (CAU, Met) over SORT-E (AGA, Ser) and vice versa (Figure 6D). The ratio of enrichments for SORT-E with CAU and AGA anticodons does not correlate with the relative abundance of their cognate codons in genes, as might be predicted by a simple model in which proteins are enriched on the basis of the relative frequency of a target codon in a gene (Figure S9). Similarly, the ratio of enrichments does not correlate with the solvent accessibility of the amino acids targeted for replacement by SORT-E, as might be expected if solvent accessibility controlled the extent to which **1** is tolerated and/or labeled at site in a protein (Figure S10). It remains possible that a combination of these factors may determine enrichment efficiency.

### SORT-E in a Multicellular System

We have previously demonstrated a modular system for SORT labeling in the fly *Drosophila melanogaster* (Elliott et al., 2014b). In this system *PylRS* is expressed from a promoter that contains a GAL4 upstream activating sequence (*UAS-PylRS*), and its cognate tRNA_XXX_ is expressed from a *PylT*_*XXX*_ gene on a U6 promoter. By crossing *UAS-PylRS/PylT*_*XXX*_ flies with flies that express the GAL4 transcription factor in specific cells at specific times, we were able to effect cell-specific proteome labeling. For example, we previously demonstrated selective SORT-M labeling in the germ cells of the fly ovary from stage 5 of oogenesis onward, using *nos-vp16-GAL4*, *PylRS*, *PylT*_*UGC*_ flies (Elliott et al., 2014b).

**Figure 7:**
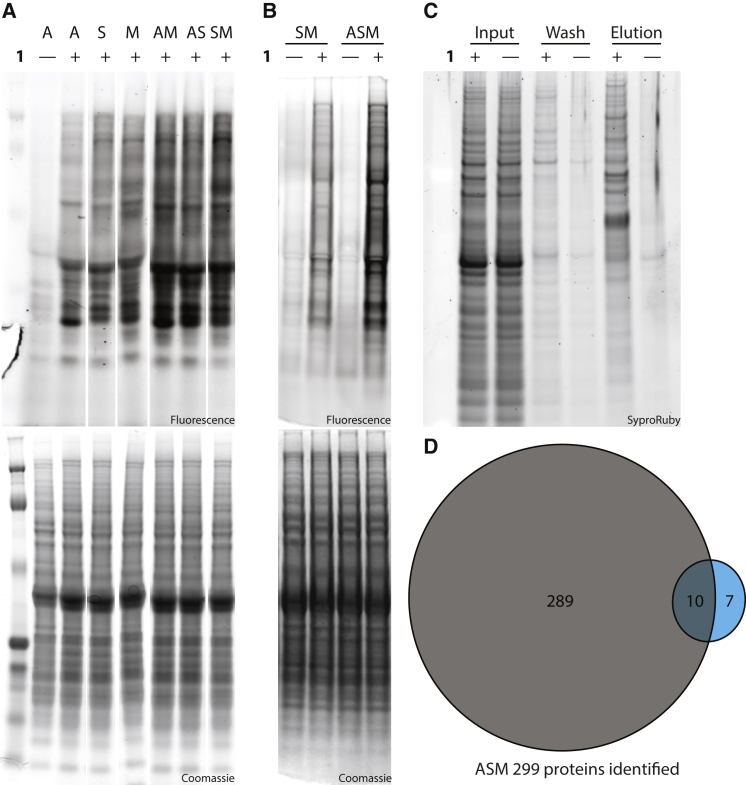
SORT-E from the Germ Cells of Fly Ovaries (A) Proteome labeling from fly ovaries is enhanced by combining two *PylT*_*XXX*_ genes with distinct anticodon sequences. Ovaries were harvested from 15 females expressing *PylRS* and *PylT*_*XXX*_ fed normal food containing **1** (10 mM), or in the control lane from 15 females not expressing *PylRS* and *PylT*_*XXX*_ but fed normal food containing **1** (10 mM). Protein lysates were labeled with tetrazine-fluorophore conjugate **7** (4 μM, Figure S11). *PylT*_*XXX*_ corresponds to variants of *PylT* bearing anticodon A = *PylT*_*UGC*_ (Ala); S = *PylT*_*GCU*_ (Ser), and M = *PylT*_*CAU*_ (Met) or combinations bearing two variants of *PylT*_*xxx*_, for example, AM = *PylT*_*UGC*_ (Ala) and *PylT*_*CAU*_ (Met). The data shown are from a gel in which intervening lanes have been removed (indicated by white space). The full gel is detailed in Figure S11. (B) Proteome labeling from fly ovaries is further enhanced by combining three *PylT*_*XXX*_ genes with distinct anticodon sequences. Experiment performed as in (A). The data shown are from a gel in which intervening lanes have been removed (indicated by white space). The full gel is detailed in Figure S11. (C) SORT-E from the germ cells of *nos-vp16-GAL4*, *PylRS*, *PylT* (Ala, Met, Ser) fly ovaries. Ovaries from flies fed amino acid **1** or control flies not fed the amino acid were lysed. Lysates were labeled with **2** and tagged proteins captured with streptavidin beads, non-specifically bound proteins were washed away, and specifically captured proteins were eluted by cleavage of **2**. All steps were performed as described for *E. coli* without further optimization. Aliquots from the initial lysate, the final wash, and eluted fractions were analyzed by SDS-PAGE. (D) Venn diagrams representing the number of proteins identified by MS from the SORT-E elution from flies fed **1** (gray) and the no-amino-acid control (blue).

To demonstrate that we can extend SORT-E to multicellular systems, we performed SORT tagging and enrichment of the proteome from the germ cells of fly ovaries. To enhance proteome tagging efficiency, and potentially proteome coverage, we first created flies containing additional copies of *PylT* with distinct anticodons. We found that the proteomes of flies containing two *PylT* genes with distinct anticodons, drawn from the group *PylT*_*UGC*_ (Ala) and *PylT*_*CAU*_ (Met), *PylT*_*GCU*_ (Ser), were more efficiently labeled than flies containing any one of these PylT_XXX_ sequences alone (Figures 7A and S11). Moreover, we found that the proteomes of flies containing *PylT*_*UGC*_ (Ala), *PylT*_*CAU*_ (Met), *PylT*_*GCU*_ (Ser) were more efficiently labeled than the proteomes of flies containing the most efficient pair of *PylT*_*XXX*_ variants (Figures 7B and S11) and used these flies, henceforth referred to as *PylRS*, *PylT* (Ala, Met, Ser) flies, for further experiments.

*nos-vp16-GAL4*, *PylRS*, *PylT* (Ala, Met, Ser) flies were fed with or without the addition of **1** to their food to provide SORT-labeled flies and genetically matched control flies that were not labeled via SORT. The proteins from the ovaries of each group of flies were extracted and subjected to the TDB-mediated enrichment procedure described herein for *E. coli*, without further optimization. Using this approach we were able to selectively enrich the proteome from the germ cells of the ovaries of flies fed amino acid **1** (Figure 7C). Additional experiments suggested that using 2-fold more nos-vp16-GAL4, *PylRS*, *PylT* (Ala, Met, Ser) ovary lysate than *PylRS/PylT*_*GCU*_ *E. coli* lysate as an input yielded comparable amounts of specifically eluted proteins (Figure S12). This suggests that the efficiency of SORT tagging in this fly line approaches that in *E. coli*. To further quantify the specificity of the approach for identifying labeled proteins from the fly ovary, we performed small-scale in-gel tryptic digests and LC-MS/MS on 2 μg of the eluted proteins. We identified 299 proteins in flies fed amino acid **1** and 17 proteins in control flies that were not fed the amino acid (Figure 7D and Table S3). The proteins shared between the two samples, on which we have statistically significant data, show amino acid-dependent enrichment (Figure S13). These data clearly demonstrate that SORT-E can be used to enrich tagged proteins from a multicellular system.

## Discussion

We have developed a TDB probe for the covalent capture of proteins containing **1**.

We have taken advantage of the site-specific incorporation of **1**, via genetic code expansion, to carefully characterize both the reaction of this probe with proteins containing **1** at specific sites and the reductive cleavage of the probe, ensuring that both steps are quantitative. We have demonstrated that proteomes labeled with **1** via SORT at diverse codons can be efficiently enriched (SORT-E) and then identified, demonstrating that inverse electron-demand Diels-Alder reactions can be used for the enrichment and identification, in addition to the imaging and control, of biomolecules. We anticipate that our approach may be extended to the capture, enrichment, and identification of proteins and other biomolecules that may be labeled with strained, or otherwise activated, alkenes or alkynes.

We demonstrate that SORT-E does not preferentially identify proteins of most molecular weights, and that SORT-E shows a slight bias toward the identification of low-abundance proteins, which should aid their identification. We find that SORT-E at different codons leads to the enrichment of many proteins with different efficiencies. While it has previously been argued that labeling at methionine is sufficient to cover the proteome (Yuet and Tirrell, 2014), as all proteins contain an N-terminal methionine we find that numerous proteins are more effectively enriched and detected via labeling at a non-methionine codon. Our results also suggest that coverage may be increased by tagging the proteome using tRNAs that target several codons. Moreover, we demonstrate that SORT-E can be used to selectively enrich the proteomes, tagged in response to several codons, from specific cells in the fly.

Because azide/alkyne cycloadditions and cyclopropene/tetrazine cycloadditions are mutually orthogonal bio-orthogonal reactions (Sachdeva et al., 2014, Shih et al., 2014), it will be possible to label distinct biomolecules in a single sample with azides or alkynes and cyclopropenes, and selectively and independently enrich and identify each population of labeled biomolecules. Given the diversity of biomolecules that can now be labeled with azides and strained alkenes, this opens up many exciting new experimental possibilities.

We anticipate that the combination of SORT, which we have previously demonstrated can be used to label the proteomes of selected cells within a whole organism, and the enrichment strategies we have carefully characterized here will enable the identification of proteins expressed in specific cells at specific times in development, disease progression, and learning and memory. Indeed, we are currently using SORT-E, in an expanding set of multicellular systems, to define cell-specific proteomes.

## Significance

**Well-characterized methods for tagging and enriching the proteomes of genetically targeted cells will provide a foundation for cell-specific proteomics in multicellular systems and whole animals. Such approaches are essential for defining how the distinct proteomes of cells in a body, or other multicellular system, carry out differentiated functions. Co-translational labeling of proteomes in *E. coli* and genetically targeted cells in the fly with a cyclopropene group, coupled with covalent capture via a tetrazine-biotin probe containing a cleavable linker, enables us to enrich labeled proteins from genetically targeted cells in an approach we term stochastic orthogonal recoding of translation with enrichment (SORT-E). Our approach allows the tagging of proteins in response to distinct codons. We demonstrate that tagging at distinct codons leads to different enrichments, suggesting that labeling at more than one codon enhances proteome coverage. We anticipate that SORT-E will enable the definition of cell-specific proteomes in animals during development, disease progression, and learning and memory.**

## Experimental Procedures

Sources of chemicals, details of chemical synthesis and MS, data analysis, and computer code used for analysis are provided in Supplemental Experimental Procedures.

### SORT-E in *E. coli*

SORT-E involves chemoselective labeling of proteomes tagged with **1** by TDB conjugate **2**, and enrichment and elution of SORT-E proteins.

For capture, enrichment, and elution of tagged proteins, 500 μl of cleared cell lysate (3.5–4 mg of 8 mg ml^−1^ lysate) was typically used. To 500 μl of cleared cell lysate, DTT (1 mM) was added and mixed with gentle vortexing, and the mixture was incubated at room temperature for 45 min. Iodoacetamide (5.5 mM) was added, mixed with gentle vortexing, then incubated for 30 min. **2** (5 μl, 20 μM final concentration, from a 2 mM stock in DMSO) was added, the reactions mixed by gentle vortexing, and the samples incubated in the dark overnight with end-over-end rotation (Hula mixer). 10-μl aliquots were taken at this stage as input sample for subsequent SDS-PAGE analysis. The samples were then diluted with PBS to a final volume of 5 ml and high-capacity streptavidin beads (150 μl of settled resin, pre-equilibrated in PBS, Thermo Scientific Streptavidin Agarose resin) added. The beads were incubated with end-over-end rotation (Hula mixer) for 1.5 hr and then collected by gravity filtration through a column (Poly-Prep chromatography column, Bio-Rad 731-1550). The beads were resuspended in urea buffer (500 μl, 8 M urea, 25 mM Tris, [pH 8]) and transferred to a smaller spin column (Mini Bio-spin chromatography column, Bio-Rad 731-1550). The beads were collected by mild centrifugation (1,000 rpm, 5-s pulse) and washed 2× urea buffer (500 μl) then 2 × 1% SDS in PBS (500 μl) with 15-min incubation times between each wash. The final wash acts as a control for the Na_2_S_2_O_4_ specific elution; the beads were resuspended in 1% SDS in PBS (150 μl) and incubated at room temperature for 30 min with end-over-end rotation (Hula mixer). The beads collected by mild centrifugation (1,000 rpm, 5-s pulse) and the supernatant kept for subsequent analysis by SDS-PAGE. Specifically bound proteins were then eluted by resuspending the beads in 1% SDS in PBS supplemented with 25 mM Na_2_S_2_O_4_ (150 μl) and incubating at room temperature for 30 min with end-over-end rotation (Hula mixer). The supernatant was collected by mild centrifugation (1,000 rpm, 5-s pulse) and analyzed by SDS-PAGE.

### Fly Lines and Culture Conditions

All flies were grown at 25°C on standard Iberian medium. Flies were fed **1** by mixing dried yeast with a solution of **1** (10 mM) to form a paste. This paste was added as a supplement to the normal Iberian fly food for a minimum of 24 hr and the yeast was changed daily.

Double- and triple-sense codon lines were created by recombination using the original lines FT58 (A = *PylT*_*UGC*_, Ala), FT60 (S = *PylT*_*GCU*_, Ser), FT62 (L = *PylT*_*CAG*_, Leu), and FT63 (M = *PylT*_*CAU*_, Met) (Elliott et al., 2014b). Trans-heterozygous virgins were collected for each pairwise combination of sense codon (AS, FT58/FT60; AL, FT58/FT62; AM, FT58/FT63; SL, FT60/FT62; SM, FT60/FT63; and LM, FT62/FT63) and crossed to males of the third chromosome balancer stock *w*;;TM3/TM6. Potential recombinant males were identified based on eye color, and individuals were backcrossed to virgins of *w*;;TM3/TM6 to make a balanced stock. Recombinant lines were then screened by crossing to *nos-vp16-GAL4* virgins (Bloomington 4937) to create FT58-60/*nos-vp16-GAL4* (AS), FT58-62/*nos-vp16-GAL4* (AL), etc., and compared with the original single-sense codon lines FT58/*nos-vp16-GAL4*, FT60/*nos-vp16-GAL4*, FT62/*nos-vp16-GAL4*, and FT63/*nos-vp16-GAL4*. The females were fed 10 mM **1** for 24–48 hr, after which the ovaries were extracted from 15 females of the indicated genotype and labeled with 4 μM **7** for 2 hr as described by Elliott et al. (2014b).

Triple-sense codon lines were generated in a similar manner. In this case the double-sense codon lines were crossed to a different single-sense codon line to generate trans-heterozygotes with three different sense codons. In this case not all combinations produced viable trans-heterozygotes and in some cases trans-heterozygotes were viable but gave no potential recombinant males. Successful combinations were FT58-63 (AM)/FT60 (S), FT60-63 (SM)/FT62 (L), and FT58-60 (AS)/FT63 (M), which generated the lines FT58-60-63 (ASM) and FT60-62-63 (SLM). These lines were screened by crossing to nos-vp16-GAL4 virgins and compared with the double-sense codon lines as described above (Elliott et al., 2014b).

### SORT-E from *D. melanogaster*

Ovaries were dissected from 250 female flies of FT58-60-63/*nos-vp16-GAL4* (ASM), which had been fed normal food either supplemented with **1** (10 mM) or without **1**. The ovaries were homogenized into 8 M urea and 15 mM Tris (250 μl) and the resultant protein lysate clarified by filtration. A Bradford assay was used to determine the protein concentration. For SORT-E and subsequent MS, typically 7 mg of fly ovary protein was used and labeled with **2** (20 μM) in an identical procedure as described in the protocol for *E. coli* above.

## Author Contributions

T.S.E. performed all chemical and biochemical experiments with *E. coli*. A.B., F.M.T., and T.S.E. performed the biochemical experiments with *D. melanogaster*. T.S.E. and S.D.F. analyzed the data. J.W.C. and T.S.E. wrote the paper with input from all authors.

## Supplemental Information

Document S1. Supplemental Experimental Procedures, Figures S1–S13, and Supplemental Program

Table S1. Protein Identities and LFQ of SORT-E from *E. coli*

Table S2. Protein Identities and Quantification of SORT-E via 9-Plex TMT

Table S3. Protein Identities and LFQ of SORT-E from *D. melanogaster*

Document S2. Article plus Supplemental Information
