# Supplementary material for: Tagging and Enriching Proteins Enables Cell-Specific Proteomics
Source: Cell Chem Biol. 2016 Jul 21;23(7):805–15. doi: 10.1016/j.chembiol.2016.05.018 (PMC4959846; doi:10.1016/j.chembiol.2016.05.018)
Supplement: Document S2. Article plus Supplemental Information [file mmc5.pdf]

Article

Cell Chemical Biology

Tagging and Enriching Proteins Enables Cell-Specific Proteomics

Highlights

- A tetrazine-biotin probe containing a cleavable linker was created
- Proteomes labeled with cyclopropene amino acids were enriched and identified
- Proteome coverage is increased by targeting the amino acids to multiple codons
- Cell-specific proteomics was accomplished in the fly

Authors

Thomas S. Elliott, Ambra Bianco, Fiona M. Townsley, Stephen D. Fried, Jason W. Chin

Correspondence

chin@mrc-lmb.cam.ac.uk

In Brief

Elliott et al. synthesize a cleavable tetrazine-biotin probe enabling identification of proteins that are tagged and enriched via stochastic orthogonal recoding of translation with enrichment (SORT-E). Proteome tagging at multiple codons increases coverage, and the authors demonstrate that SORT-E at several codons enables cell-specific proteomics in the fly.

Elliott et al., 2016, Cell Chemical Biology *23*, 805–815
July 21, 2016 © 2016 MRC Laboratory of Molecular Biology.
Published by Elsevier Ltd.
http://dx.doi.org/10.1016/j.chembiol.2016.05.018

CrossMark

CellPress

Please cite this article as: Elliott et al., Tagging and Enriching Proteins Enables Cell-Specific Proteomics, Cell Chemical Biology (2016), http://dx.doi.org/10.1016/j.chembiol.2016.05.018

Cell Chemical Biology
Article

Tagging and Enriching Proteins Enables Cell-Specific Proteomics

Thomas S. Elliott,[1] Ambra Bianco,[1,2] Fiona M. Townsley,[1] Stephen D. Fried,[1] and Jason W. Chin[1,*]
[1]Medical Research Council Laboratory of Molecular Biology, Francis Crick Avenue, Cambridge CB2 0QH, UK
[2]Present address: AstraZeneca, Darwin Building, Unit 310 Cambridge Science Park, Milton Road, Cambridge CB4 0FZ, UK
*Correspondence: chin@mrc-lmb.cam.ac.uk
http://dx.doi.org/10.1016/j.chembiol.2016.05.018

SUMMARY

Cell-specific proteomics in multicellular systems and whole animals is a promising approach to understand the differentiated functions of cells and tissues. Here, we extend our stochastic orthogonal recoding of translation (SORT) approach for the cotranslational tagging of proteomes with a cyclopropene-containing amino acid in response to diverse codons in genetically targeted cells, and create a tetrazine-biotin probe containing a cleavable linker that offers a way to enrich and identify tagged proteins. We demonstrate that SORT with enrichment, SORT-E, efficiently recovers and enriches SORT tagged proteins and enables specific identification of enriched proteins via mass spectrometry, including low-abundance proteins. We show that tagging at distinct codons enriches overlapping, but distinct sets of proteins, suggesting that tagging at more than one codon enhances proteome coverage. Using SORT-E, we accomplish cell-specific proteomics in the fly. These results suggest that SORT-E will enable the definition of cell-specific proteomes in animals during development, disease progression, and learning and memory.

INTRODUCTION

Biomolecules, including proteins (Lang and Chin, 2014b; Ngo and Tirrell, 2011), DNA (Busskamp et al., 2014; Rieder and Luedtke, 2014), RNA (Asare-Okai et al., 2014), lipids (Yang et al., 2012), and sugars (Patterson et al., 2014; Prescher and Bertozzi, 2006), may be tagged with bio-orthogonal groups for many applications, including imaging, identification, and synthetic control. Global tagging of biomolecules may be achieved by feeding suitably tagged biosynthetic precursors to the natural biosynthetic machinery of the cell; an approach that requires the natural machinery to tolerate the introduction of the bio-orthogonal tag (Kiick et al., 2002). Analogs of natural precursors bearing bio-orthogonal tags may also be incorporated by engineered biosynthetic machineries with expanded substrate scope (Mahdavi et al., 2016; Ngo et al., 2013; Yuet et al., 2015). The creation of orthogonal biosynthetic machinery in cells enables the incorporation of diverse substrates without competition from endogenous substrates (Chin, 2014; Elliott et al., 2014a, 2014b; Liu and Schultz, 2010). In addition to their use for tagging biomolecules, bio-orthogonal groups have also found extensive utility in a variety of other approaches, including as reactive handles in activity-based probes of protein function (Cravatt et al., 2008).

The majority of approaches to bio-orthogonal labeling reported to date take advantage of copper (I)-catalyzed reactions between azides and alkynes (Hong et al., 2009; Wang et al., 2003). These components are relatively small, and so metabolic precursors modified with azides and alkynes may be tolerated by the natural biosynthetic machinery of cells. However, certain azides are prone to reduction in the cellular milieu (Mancuso et al., 2013), and the reaction between azides and linear alkynes depends on copper (I) catalysis, which requires optimization to avoid damage to biomolecules (Hong et al., 2009). Strain-promoted reactions between azides and strained alkynes remove the copper dependence (Agard et al., 2004). Although many of these reactions are relatively slow, varying the groups appended to the azide or strained alkyne has led to increases in reaction rates. However, despite the substantial impact of azide-alkyne cycloadditions on biological discovery, additional approaches for the labeling of biomolecules and probes are required.

Recently, inverse electron-demand Diels-Alder reactions between strained alkenes, alkynes, and tetrazines have been recognized as excellent bio-orthogonal reactions (Blackman et al., 2008; Devaraj et al., 2008; Lang et al., 2012b; Patterson et al., 2012; Yang et al., 2012). These reactions can be exceedingly rapid ($k \sim 10^6$ M^{-1} s^{-1}), proceed without catalysts, and produce nitrogen gas as the only by-product (Lang and Chin, 2014a). Alkenes and alkynes have been used to tag proteins, DNA, RNA, lipids, and sugars, enabling their labeling with tetrazine-fluorophore conjugates for imaging (Agarwal et al., 2015; Asare-Okai et al., 2014; Busskamp et al., 2014; Denk et al., 2014; Kurra et al., 2014; Lang et al., 2012a, 2012b; Nikic et al., 2014; Patterson et al., 2014; Pyka et al., 2014; Selvaraj et al., 2015; Uttamapinant et al., 2015; Yang et al., 2012). However, while azide-alkyne reactions form the basis of strategies to enrich tagged biomolecules for their identification by mass spectrometry (MS) (Bagert et al., 2016; Chesarino et al., 2014; Dieterich et al., 2006, 2007; Kleiner et al., 2015; Smeekens et al., 2015; Vanbeselaere et al., 2012; Zhang et al., 2013; Zheng et al., 2013), there are currently no methods for enriching and identifying biomolecules labeled with strained alkenes or alkynes through inverse electron-demand Diels-Alder reactions.

We, and others, have demonstrated that norbornene- (Kaya et al., 2012; Lang et al., 2012a; Plass et al., 2012), bicyclononyne- (Borrmann et al., 2012; Lang et al., 2012b),

This is an open access article under the CC BY license (http://creativecommons.org/licenses/by/4.0/).

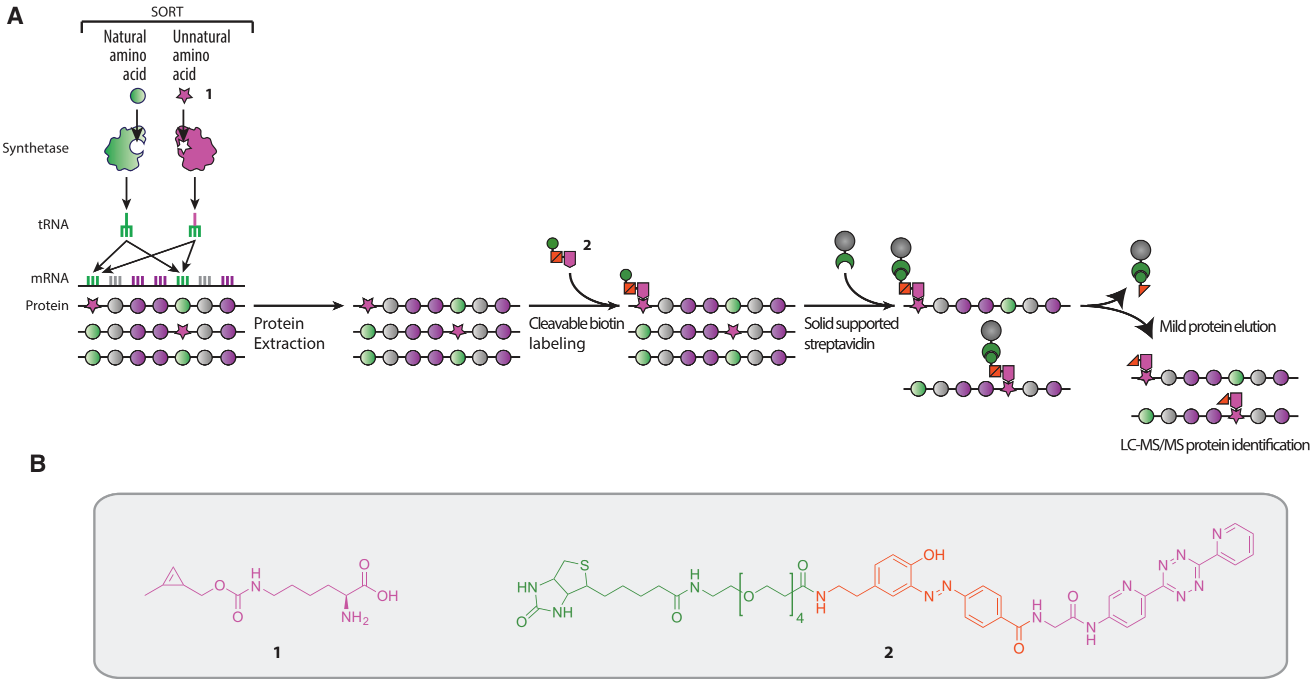

Figure 1. SORT-E Extends Proteome Tagging and Labeling to Protein Capture and Enrichment via Inverse Electron-Demand Diels-Alder Reactions with TDB, 2

(A) In SORT an unnatural amino acid (pink star) is recognized by an orthogonal pyrrolysyl-tRNA synthetase, and used to aminoacylate the cognate $tRNA_{XXX}$ bearing a sense-decoding anticodon. This leads to the substoichiometric incorporation of the unnatural amino acid in response to the targeted sense codons. This approach has been implemented with the unnatural amino acid **1**. In SORT-E the proteins are extracted from cells and SORT-tagged proteins are captured with the tetrazine diazobenzene biotin compound, **2**, before capture on streptavidin beads. The beads are washed and enriched proteins are specifically eluted for detection by MS.

(B) Structures of the unnatural amino acid *N*ε-(((2-methylcycloprop-2-en-1-yl)methoxy)carbonyl)-l-lysine, **1**, and the cleavable TDB (**2**), used in SORT-E.

trans-cyclo-octene- (Lang et al., 2012b; Plass et al., 2012), and 1,3-disubstituted cyclopropene-containing (Elliott et al., 2014b) amino acids can be site-specifically incorporated into proteins in response to an amber codon introduced into a gene of interest using the pyrrolysyl-tRNA synthetase(PylRS)/$tRNA_{CUA}$ pair and its active-site derivatives. This has enabled the imaging and control of protein function in vivo through the labeling of the tagged protein with appropriately functionalized tetrazine conjugates (Lang et al., 2012a, 2012b; Plass et al., 2012; Tsai et al., 2015; Uttamapinant et al., 2015).

An emerging application of bio-orthogonal labeling is for the imaging, and in some cases identification, of proteins expressed in particular cells at particular times in whole organisms (Elliott et al., 2014a, 2014b; Erdmann et al., 2015; Yuet et al., 2015). We reported the first solution to this problem: stochastic orthogonal recoding of translation (SORT; Figure 1A) in which the CUA anticodon of $tRNA_{CUA}$ is converted to a variety of triplets (XXX) that are complementary to diverse sense codons. This approach enables the incorporation of diverse amino acids in response to diverse sense codons (Elliott et al., 2014b). We demonstrated that **1** is a substrate for PylRS, and can be used for SORT (Elliott et al., 2014b). Because SORT uses an orthogonal synthetase and tRNA, there is no competition for the active site of the synthetase between **1** and natural substrates. Thus, SORT can be used to label newly synthesized proteins in cells and animals without the use of minimal media or starvation. Moreover, because the approach is genetically targeted it can be used to fluorescently label and identify newly synthesized proteins from specific cells, at specific developmental stages, within an animal via SORT with modification (SORT-M), in which **1** is labeled with tetrazine-fluorophore conjugates via an inverse electron-demand Diels-Alder reaction (Elliott et al., 2014b). We hypothesized that directing SORT to distinct codons by the use of tRNAs with distinct anticodons would lead to the labeling of different proteins with different efficiency, and that combining information on labeling at different codons may enable greater coverage of the proteome than labeling at any one codon (Elliott et al., 2014b).

Here we report SORT-E (SORT with enrichment) for the covalent capture and enrichment of SORT-labeled proteins via an inverse electron-demand Diels-Alder reaction with a tetrazine probe (Figure 1A). We demonstrate that SORT-E allows the substantial enrichment of proteomes tagged with **1** in response to diverse codons, and enables the identification and quantification of enriched proteins by MS (Bantscheff et al., 2012). We find that SORT-E does not preferentially identify proteins by molecular weight, but shows a slight bias toward the identification of low-abundance proteins, which should aid their identification. SORT-E at different codons leads to the enrichment of many proteins with different efficiencies, suggesting that proteome coverage may be increased by performing SORT experiments with several anticodon variants. To demonstrate the utility of SORT-E in a multicellular system, we create flies that tag newly

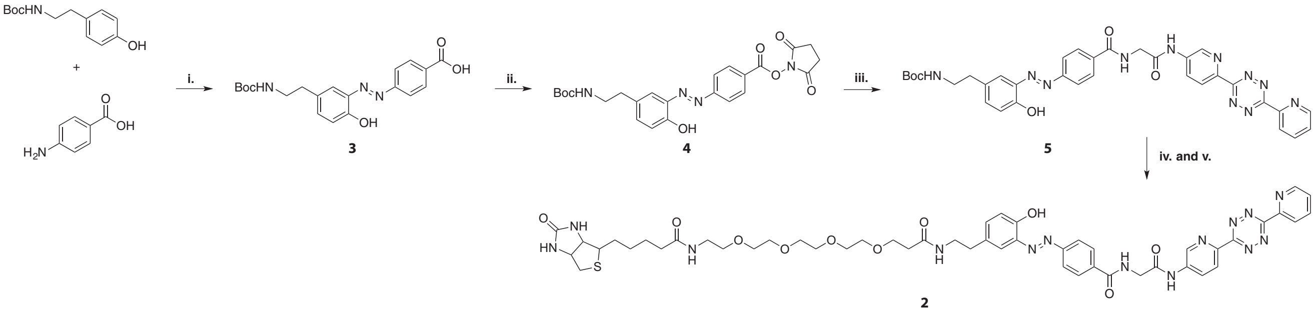

Figure 2. Synthesis of Compound 2

i. (a) 5 N HCl, $NaNO_2$, 0°C, (b) $NaHCO_3$, acetone, 0°C, 71% yield; **ii**. *N*-Hydroxysuccinimide, dimethylformamide (DMF), 1-ethyl-3-(3-dimethyl aminopropyl) carbodiimide, dimethylaminopyridine, room temperature, 48% yield; **iii**. Aminotetrazine, Et_3N, DMF, room temperature, 28% yield; **iv**. trifluoroacetic acid, CH_2Cl_2, room temperature, quantitative yield; **v**. NHS-PEG_4-Biotin (Thermo Scientific), DMF, Et_3N, room temperature, 18% yield.

synthesized proteins in ovary germ cells in response to several codons, and enrich and identify SORT-tagged proteins from these cells.

RESULTS

Design and Synthesis of a Cleavable Tetrazine Diazobenzene Biotin Probe

We designed a probe, tetrazine diazobenzene biotin (TDB, **2**; Figure 1B), to enable the enrichment, purification, and identification of molecules labeled with strained alkenes or alkynes that undergo inverse electron-demand Diels-Alder reactions with tetrazines. The design of our probe builds on previous work developing and optimizing reagents for the covalent capture of proteins through azide-alkyne cycloadditions (Yang et al., 2010). Our probe has three components: (1) a tetrazine, (2) a biotin moiety, and (3) a cleavable diazobenzene linker between the tetrazine and biotin moieties (Szychowski et al., 2010; Verhelst et al., 2007) (Figure 1B). The 3,6-dipyridyl tetrazine is designed to facilitate rapid and selective inverse electron-demand Diels-Alder reactions of the probe with the tagged molecules in a sample. This group has good stability, and we previously reported that a TAMRA derivative of this tetrazine reacts with **1** at position 150 in GFP with a rate constant of 27 M^{-1} s^{-1} (Elliott et al., 2014b). While azide-strained alkyne cycloaddition reactions have been reported that are only 10-fold slower than this reaction (Dommerholt et al., 2014), these reactions use azides coupled to aromatic rings with electron-deficient substituents that cannot be incorporated into proteins for proteome labeling. The rate constants reported for the reactions between cyclo-octynes and azides, which are similar to those used for proteome labeling (Dommerholt et al., 2014), are 100- to 1,000-fold lower than the rate constant for the reaction of **1** in GFP with 3,6-dipyridyl tetrazines. The biotin moiety in TDB was designed to enable the capture of the labeled proteins on streptavidin-coated beads and facilitate the removal of unlabeled molecules in the sample by washing. The diazobenzene was designed to enable the selective release of labeled molecules, via reductive cleavage, for identification by MS.

We developed a succinct synthesis of TDB probe **2** (Figure 2). Starting from two commercially available materials, a diazotization reaction afforded intermediate **3** in 71% yield. We found that direct coupling of carboxylate **3** with previously synthesized tetrazine amines (Lang et al., 2012a) could not be achieved using conventional carbodiimide or HATU chemistry. However, reacting a tetrazine amine with an activated ester, **4**, gave the protected diazobenzene **5** in 28% yield. Boc deprotection of **5** followed by coupling with an activated biotin conjugate gave the desired product, **2**, in 18% yield over two steps.

Characterizing the Reaction of TDB with Proteins Incorporating 1

Next we characterized (1) the labeling reaction between the probe and proteins in which we site-specifically incorporated **1**, and (2) the subsequent reductive cleavage of the linker by MS (Figure 3A). We produced T4 lysozyme (K-83-**1**)-His6, ubiquitin (K-6-**1**)-His6, and ubiquitin (K-48-**1**)-His6 from the corresponding genes (*T4 lysozyme (83TAG)-His6*, *Ubiquitin (6TAG)-His6*, and *Ubiquitin (48TAG)-His6*). Proteins were produced from *Escherichia coli* DH10B (T4 lysozyme) or BL21(DE3) (ubiquitin) expressing the PylRS/$tRNA_{CUA}$ pair, which directs the incorporation of **1** in response to the amber codon, and purified by Ni-nitrilotriacetic acid chromatography with yields of 20–40 mg/l of culture. The incorporation of **1** in each protein was confirmed by electrospray ionization MS (ESI-MS) (Figures 3B–3D and S1).

We incubated each protein with 10 molar equivalents of **2** overnight at room temperature in 8 M guanidinium chloride. ESI-MS confirmed the quantitative labeling of each protein with **2** (Figures 3B–3D). Addition of $Na_2S_2O_4$ (25 mM, room temperature) to the labeled protein led to quantitative reductive cleavage of the diazo bond within 30 min, as judged by ESI-MS (Figures 3B–3D). We further analyzed the reduced products by liquid chromatography-tandem MS (LC-MS/MS) to directly confirm the identity and position of the final protein modification (Figure S2). Taken together, these experiments demonstrate that we can quantitatively and specifically capture biomolecules bearing 1,3-disubstituted cyclopropenes using TDB, and that we can quantitatively and specifically cleave the probe to an amino benzamide once it is ligated to the biomolecule of interest.

Enriching Labeled Proteins via SORT-E

Next we demonstrated that TDB (**2**) can be used to selectively enrich proteins that have been labeled with **1** via SORT. To label

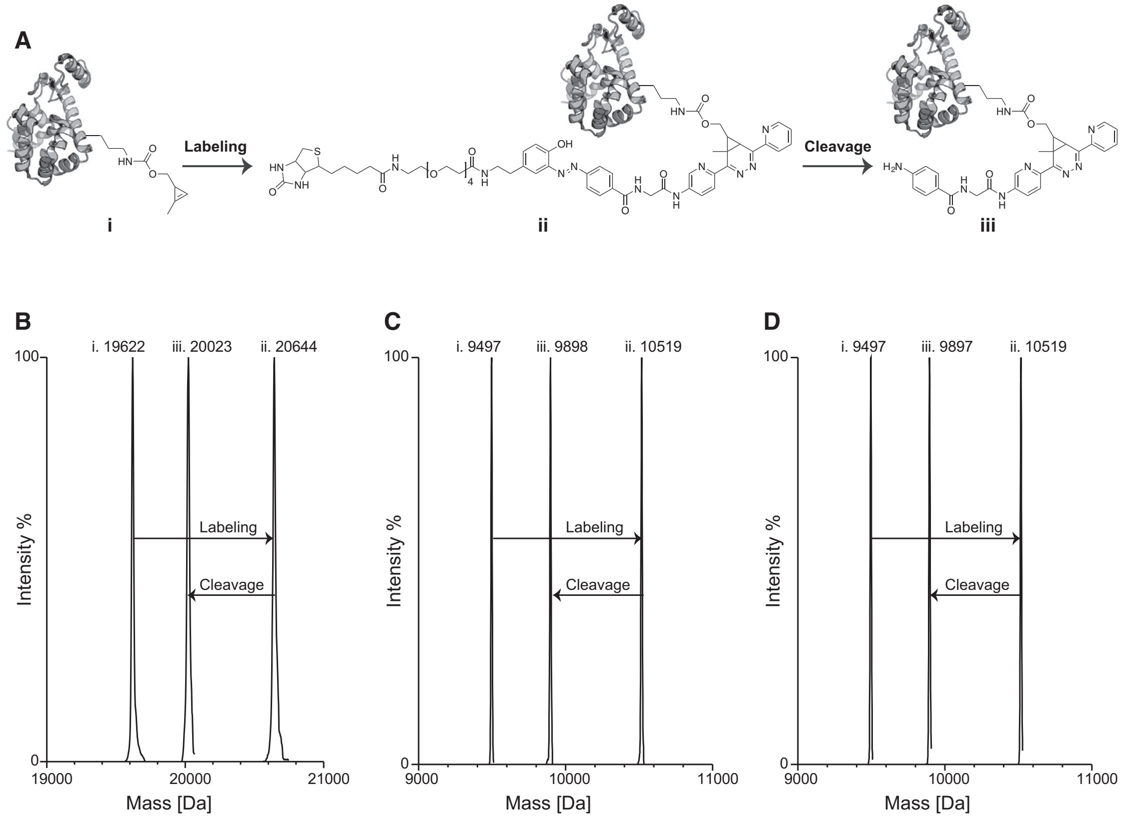

Figure 3. Quantitative Site-Specific Labeling of Genetically Encoded 1 with TDB, and Reductive Cleavage of the Diazobenzene by Mass Spectrometry

(A) Proteins with site-specifically incorporated **1** (**I**) were labeled with 20 μM **2** at room temperature overnight to furnish (**II**). Treatment of (**II**) with 25 mM $Na_2S_2O_4$ for 30 min generates the cleavage product (**III**).

(B) Deconvoluted mass spectra for species (**I**), (**II**), and (**III**) for T4-lysozyme (K-83-**1**)-His6.

(C) Deconvoluted mass spectra for species (**I**), (**II**), and (**III**) for ubiquitin (K-6-**1**)-His6.

(D) Deconvoluted mass spectra for species (**I**), (**II**), and (**III**) for ubiquitin (K-48-**1**)-His6. For each protein, mass increases by 1,022 Da upon conjugation with **2** (as expected), and then decreases by 621 Da upon reductive cleavage of the biotin moiety (as expected).

the proteomes of cells, we grew cultures containing one of four PylRS/tRNA$_{XXX}$ pairs (where XXX = AGA, GCU, CAU, or UUU) in the presence of **1** (0.1 mM) (Elliott et al., 2014b). In control experiments we omitted **1** from the media. To confirm that **1** was incorporated into the proteome via SORT, we lysed the cells and treated a portion (240 μg) of each lysate with a tetrazine-fluorophore conjugate **6** (Figure S3) (Lang et al., 2012a). As expected, we see fluorescent labeling of the proteomes derived from cells grown in the presence of **1**, but almost no labeling of the proteomes derived from cells grown in the absence of **1** (Figure S3).

To enrich SORT-labeled proteomes, we labeled protein lysates (500 μl, at 8 mg/ml protein concentration) with **2** (20 μM, final concentration) at room temperature overnight. The specific and rapid reaction of **2** with 1,3-disubstituted cyclopropenes allowed us to use a relatively low concentration of **2**. Using a low concentration of **2** allowed us to add the streptavidin-coated beads (that have a high capacity, 10 mg biotinylated protein per 1 ml of settled resin) directly to the reaction mixture, as the capture of conjugates between proteins and **2** by the streptavidin beads is not limited by the binding of free **2** to the streptavidin beads. This is in contrast to other covalent enrichment approaches, which use much more labeling reagent in an effort to drive slower reactions to completion (equivalent azide reaction requires five times more linker, 1 mM $CuSO_4$, 1 mM tris(2-carboxyethyl)phosphine, and an expensive ligand [Yang et al., 2010]) and precipitate proteins overnight to remove free label prior to capture on streptavidin. By omitting the precipitation step we simplify and accelerate the experiment, and by reducing the number of handling steps we potentially reduce protein loss and sources of experimental error. We incubated the labeled lysate with the beads (1.5 hr at room temperature) prior to washing.

We washed the beads extensively to remove non-specifically bound proteins. The final wash for all samples contained very little protein (Figure 4A), consistent with the effective removal of non-specifically bound proteins by the wash steps. Following the washes, we eluted proteins that were linked to the beads through the diazo group of **2** using PBS supplemented with 1% SDS and 25 mM $Na_2S_2O_4$. Substantial amounts of protein from samples grown in the presence of **1** were eluted with $Na_2S_2O_4$ while very little protein was eluted from samples grown in the absence of **1** (Figure 4A). We conclude that our procedure

Please cite this article as: Elliott et al., Tagging and Enriching Proteins Enables Cell-Specific Proteomics, Cell Chemical Biology (2016), http://dx.doi.org/10.1016/j.chembiol.2016.05.018

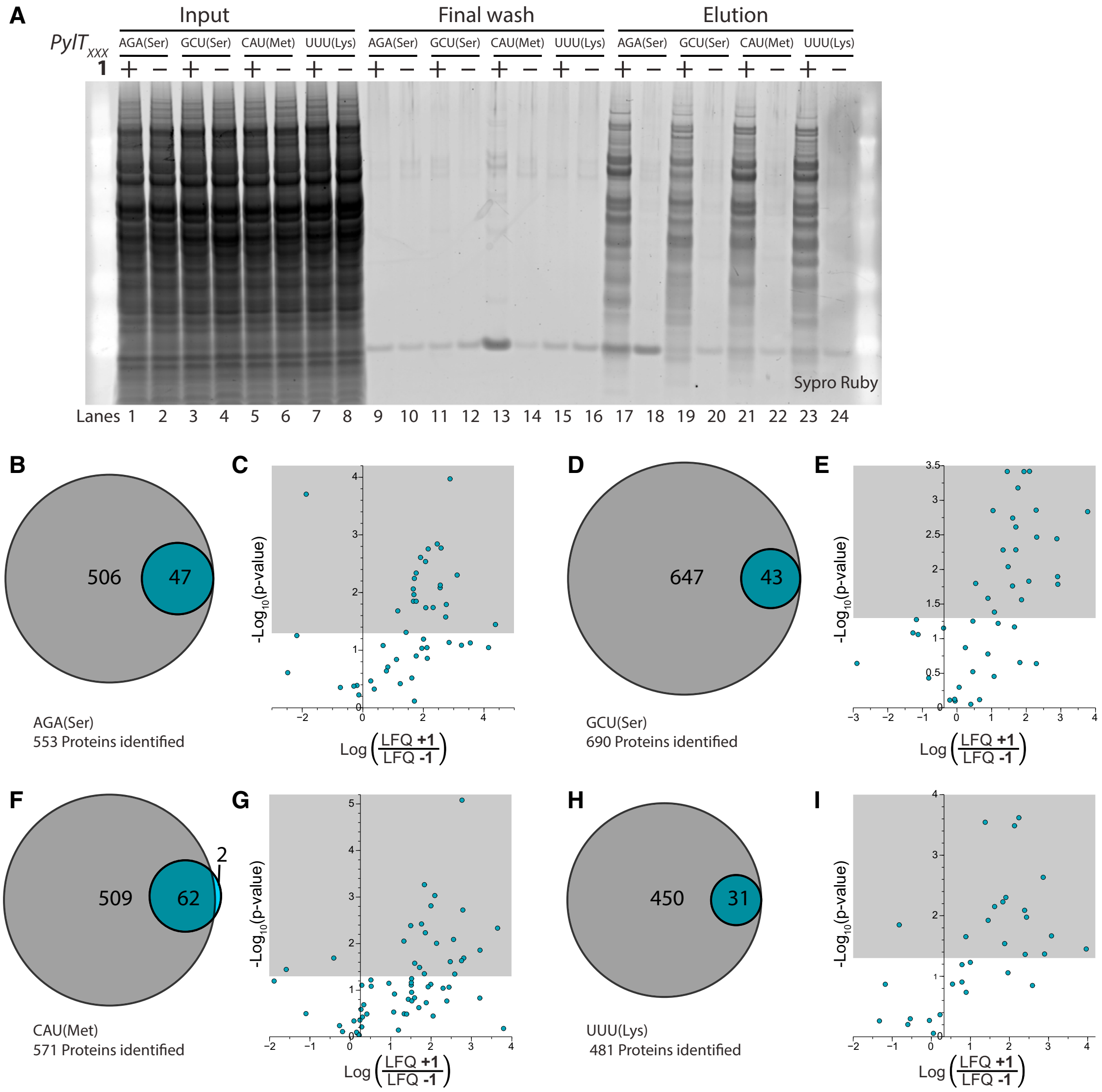

Figure 4. SORT-E Selectively Enriches Proteins Labeled with 1

(A) Cells expressing PylRS and either $tRNA_{AGA(Ser)}$, $tRNA_{GCU(Ser)}$, $tRNA_{CAU(Met)}$, or $tRNA_{UUU(Lys)}$ were grown either with or without **1** (0.1 mM). Lysates were labeled with **2** and tagged proteins captured with streptavidin beads, non-specifically bound proteins were washed away, and specifically captured proteins were eluted by cleavage of **2**. Aliquots from the initial lysate, the final wash, and eluted fraction were analyzed by SDS-PAGE.

(B, D, F, and H) Venn diagrams representing the number of proteins identified by MS from the SORT-E elution (gray) and the no-amino-acid control (blue).

(C, E, G, and I) Proteins that are identified in both the SORT-E samples (+**1**) and controls (–**1**) are significantly enriched in the +**1** samples. The volcano plots show the log ratio of the LFQ values for each protein in this subset (i.e., the enrichment factor), plotted against the log p value of the null hypothesis that there is no difference between the LFQ values. The area shaded in gray corresponds to the threshold of p values of <0.05.

enables the specific capture of proteins labeled with **1**. Quantifying the protein in the GCU (Ser) elution (Figure S4) reveals that approximately 39 μg of protein was recovered. Since the input for the pulldown was 4 mg of protein, and SORT tags targeted proteins in response to target codons with an efficiency of substantially less than 1% (Elliott et al., 2014b), this enrichment step efficiently captures tagged proteins. We conclude that our approach, which we call stochastic orthogonal recoding of translation with enrichment (SORT-E), efficiently recovers and enriches SORT-labeled proteins. To define the limits of specific enrichment, we mixed SORT-labeled lysates with increasing amounts of unlabeled lysates. With a 10-fold excess of unlabeled lysate SORT-E still leads to selective enrichment, while for a 100-fold excess of unlabeled lysate non-specific labeling approaches the level of specific labeling (Figure S5). These data demonstrate that SORT-E provides a powerful approach for enriching proteins that are substoichiometrically tagged in target cells from a vast excess of untagged proteins.

Identifying Enriched Proteins by Mass Spectrometry

To demonstrate that our approach enables the specific identification of proteins labeled with **1** by SORT and to further

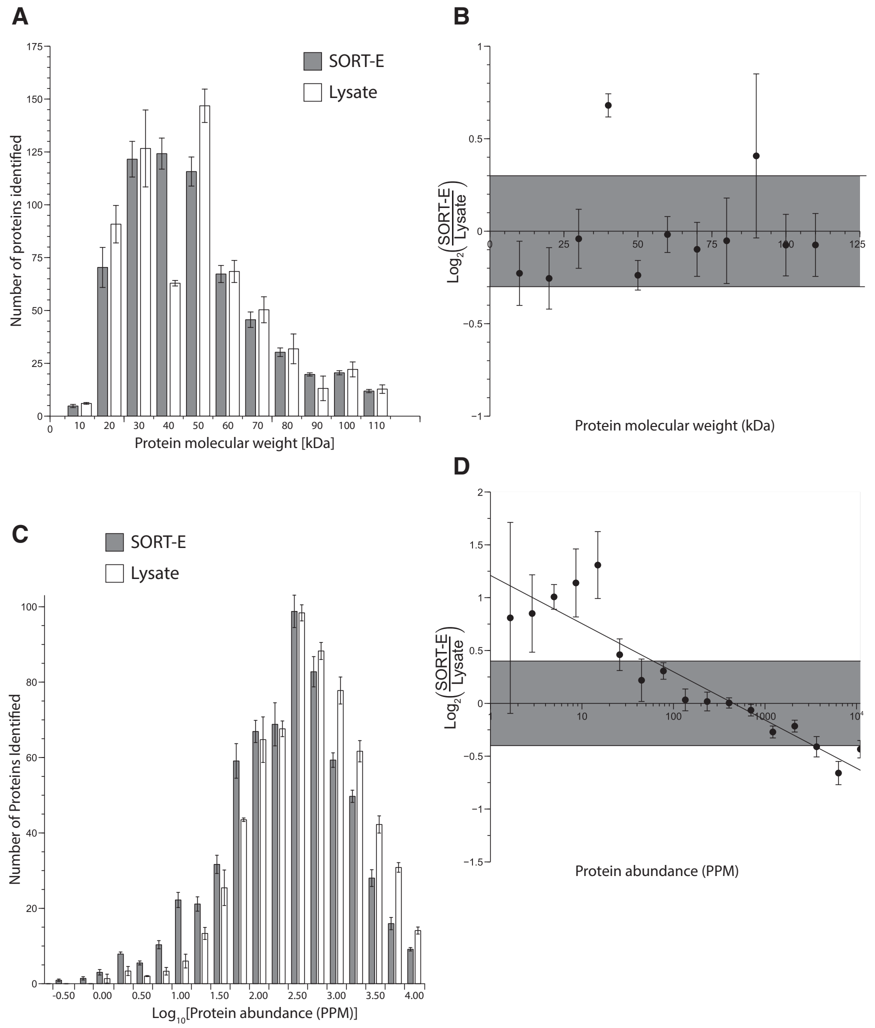

Figure 5. SORT-E Enriches Proteins of Most Molecular Weights Equally, but Preferentially Enriches Less Abundant Proteins from the Proteome

(A) Number of proteins identified, either from crude lysates (white bars) or from SORT-E (gray bars). Data are binned by molecular weight. Error bars represent the SD of three biological replicates.

(B) SORT-E identifies proteins across the full range of molecular weights of the *E. coli* proteome and shows no bias for larger or smaller proteins. The graph shows the log ratio of the number of proteins identified from SORT-E to the number of proteins identified from crude lysates, as a function of protein molecular weight, using the bins from (A). Error bars represent the SD of three biological replicates. Points that fall within the shaded area have a relative abundance of proteins before and after SORT-E that is within a factor of 1.32.

(C) Number of proteins identified, either from crude lysates (white bars) or from SORT-E (gray bars). Data are binned by protein abundance in the PAX database of the *E. coli* proteome. Error bars represent the SD of three biological replicates.

(D) SORT-E preferentially enriches low-abundance proteins. Log ratio of the number of proteins identified from SORT-E to the number of proteins identified from crude lysates, as a function of protein abundance defined in the PAX database. Error bars represent the SD of three biological replicates. Points that fall within the shaded area have a relative abundance of proteins before and after SORT-E that is within a factor of 1.32.

quantify the specificity of the approach for identifying labeled proteins, we performed in-gel tryptic digests and LC-MS/MS of the eluted proteins. For cells grown in the presence of **1** and the PylRS/$tRNA_{XXX}$ pairs we identified 553, 690, 571, and 481 proteins (for XXX = AGA, GCU, CAU, and UUU, respectively) from approximately 1.3 μg of protein (i.e., using just 3.3% of our elution) (Figures 4B–4I and Table S1). The samples from cells grown in the absence of **1** yielded a small subset of the proteins identified from cells grown in the presence of **1**, with two exceptions for CAU. We identified 47, 43, 64, and 31 proteins from cells grown in the absence of **1** (for XXX = AGA, GCU, CAU, and UUU, respectively, Figures 4B–4I). Thus 92% (±2.2%) of proteins identified from cells grown in the presence of **1** are not found in cells grown in the absence of **1**. For the remaining 8% (±2.2%) of proteins that are captured for cells grown in both the presence and absence of **1**, we compared the abundance of each protein, as judged by label free quantification (LFQ) (Figures 4C, 4E, 4G, 4I, and S6) (Cox et al., 2014). The vast majority of proteins that are found in both samples, and for which we have statistically significant data, are enriched in the cells grown in the presence of **1** (log[LFQ + **1**/LFQ − **1**] > 0).

SORT-E Enhances the Identification of Low-Abundance Proteins

To address the extent to which our approach reproduces the distribution of proteins present in the proteome, we compared enriched proteomes identified via SORT-E with the proteome prior to TDB enrichment (Figure 5A). We find that the relative abundance of proteins for the vast majority of molecular weight ranges is comparable before and after TDB enrichment (Figures 5A and 5B). However, for the molecular weight range between 30 and 40 kDa we observed an increase in the number of proteins identified after the TDB enrichment.

To address the extent to which TDB enrichment captures protein present at different abundances in the cell, we compared the distribution of proteins before and after SORT-E as a function of abundance in the cell, using the data compiled in the PAX database (Figures 5C and 5D) (Wang et al., 2012, 2015). We found that the distribution of proteins following TDB enrichment (Figure 5C) is slightly shifted to favor low-abundance proteins, with respect to the distribution of proteins prior to enrichment. This observation was further supported by additional experiments (Figure S7). This basis of this enrichment is unclear, but it may result from the TDB enrichment decreasing sample complexity

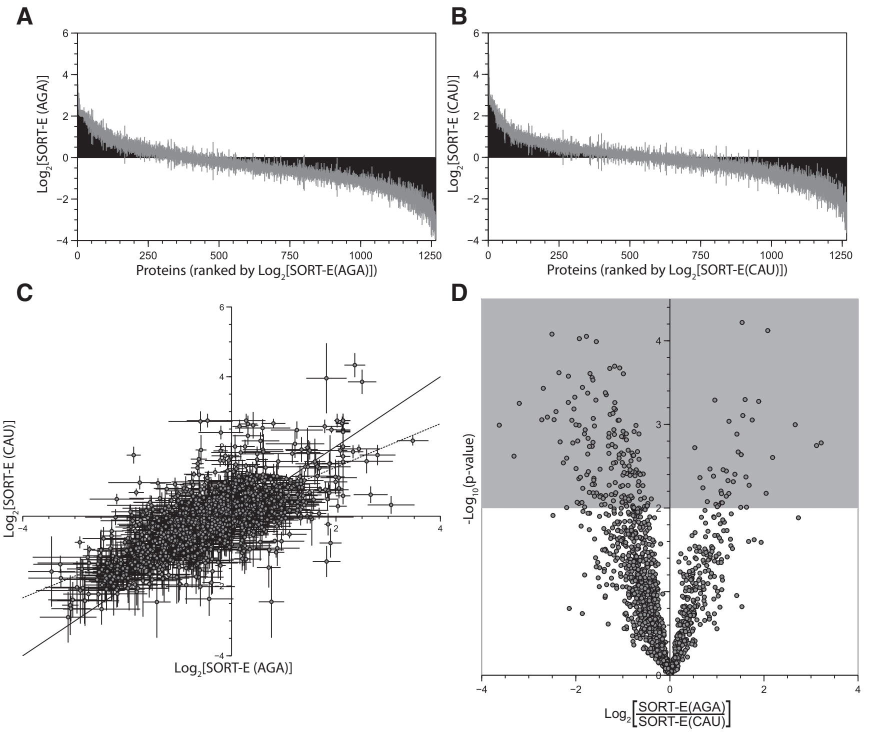

Figure 6. The Efficiency of Enrichment Depends on the Identity of the Anticodon used in SORT-E

(A) SORT-E (AGA, Ser) enriches different proteins in the proteome to different extents. The graph shows the log of the ratio of the relative abundance for proteins detected for SORT-E to the relative abundance prior to enrichment. The data were measured in a multiplexed TMT experiment in which 1,265 proteins were common to all samples. The data are ranked by this ratio from most enriched to least enriched, to show the distribution of enrichments. Error bars, shown in gray, are the SD of three biological replicates each performed in duplicate.

(B) As for (A), but for SORT-E (CAU, Met).

(C) SORT-E (AGA, Ser) and SORT-E (CAU, Met) enrichments are globally correlated. The values in (A) and (B) were plotted to examine the correlation between protein enrichments in SORT experiments that use tRNAs with two distinct anticodons (AGA and CAU). The correlation (least-squares regression line $y = 0.157 + 0.624x$; $R^2 = 0.48$, $p = 10^{-179}$) between the enrichments for each codon is indicated by the dashed line. The solid line represents $y = x$, indicating what the best fit would look like if the enrichments were identical between the two anticodons. Error bars represent the st. dev. of three biological replicates.

(D) SORT-E with distinct anticodons enriches a substantial subset of proteins with different efficiencies. The volcano plot shows the ratio of the data shown in (C), plotted against the p value of the null hypothesis that the protein is enriched with identical efficiencies for SORT-E (AGA, Ser) and SORT-E (CAU, Met). The 203 points in the upper shaded region correspond to the 16% of proteins that are significantly ($p < 10^{-2}$) more enriched by SORT-E (AGA, Ser) (right branch) or SORT-E (CAU, Met) (left branch).

and bringing some low abundance proteins into a concentration range where they can be more easily detected by MS.

SORT-E at Different Codons Enriches Different Proteins

To investigate how the relative abundance of individual proteins is affected by the TDB pulldown using SORT systems addressed to different codons, we performed multiplexed tandem mass tagging (TMT) labeling and MS (McAlister et al., 2012; Thompson et al., 2003; Werner et al., 2012) (Figures 6 and S8; Table S2). For SORT-E (CAU, Met), in which **1** is stochastically incorporated in place of Met using a tRNA with a CAU anticodon, we observed proteins that increase as a fraction of total proteins after TDB enrichment, and proteins that are unchanged or depleted as a fraction of total proteins after TDB enrichment (Figure 6A). For SORT-E (AGA, Ser), in which **1** is stochastically incorporated in place of Ser using a tRNA with an AGA anticodon, we observed proteins that increase as a fraction of total proteins after TDB enrichment, and proteins that are unchanged or depleted as a fraction of total proteins after TDB enrichment (Figure 6B). We find that, broadly, there is a positive correlation in enrichment of proteins in SORT-E (CAU, Met) and SORT-E (AGA, Ser) (Figure 6C). However, there are clearly many proteins that are significantly and preferentially enriched by SORT-E (CAU, Met) over SORT-E (AGA, Ser) and vice versa (Figure 6D). The ratio of enrichments for SORT-E with CAU and AGA anticodons does not correlate with the relative abundance of their cognate codons in genes, as might be predicted by a simple model in which proteins are enriched on the basis of the relative frequency of a target codon in a gene (Figure S9). Similarly, the ratio of enrichments does not correlate with the solvent accessibility of the amino acids targeted for replacement by SORT-E, as might be expected if solvent accessibility controlled the extent to which **1** is tolerated and/or labeled at site in a protein (Figure S10). It remains possible that a combination of these factors may determine enrichment efficiency.

SORT-E in a Multicellular System

We have previously demonstrated a modular system for SORT labeling in the fly *Drosophila melanogaster* (Elliott et al., 2014b). In this system *PylRS* is expressed from a promoter that contains a GAL4 upstream activating sequence (*UAS-PylRS*), and its cognate $tRNA_{XXX}$ is expressed from a $PylT_{XXX}$ gene on a U6 promoter. By crossing *UAS-PylRS*/$PylT_{XXX}$ flies with flies that express the GAL4 transcription factor in specific cells at specific times, we were able to effect cell-specific proteome labeling. For example, we previously demonstrated selective SORT-M labeling in the germ cells of the fly ovary from stage 5 of oogenesis onward, using *nos-vp16-GAL4*, *PylRS*, $PylT_{UGC}$ flies (Elliott et al., 2014b).

To demonstrate that we can extend SORT-E to multicellular systems, we performed SORT tagging and enrichment of the proteome from the germ cells of fly ovaries. To enhance proteome tagging efficiency, and potentially proteome coverage, we first created flies containing additional copies of *PylT* with distinct anticodons. We found that the proteomes of flies containing two *PylT* genes with distinct anticodons, drawn from the group $PylT_{UGC}$ (Ala) and $PylT_{CAU}$ (Met), $PylT_{GCU}$ (Ser), were

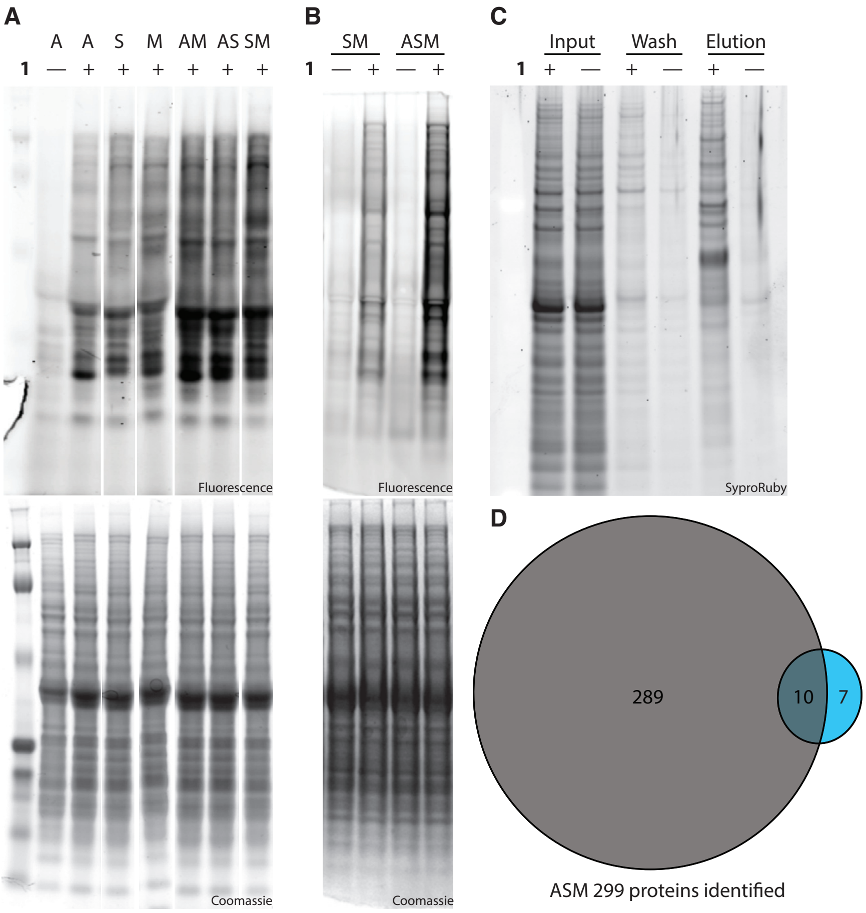

Figure 7. SORT-E from the Germ Cells of Fly Ovaries

(A) Proteome labeling from fly ovaries is enhanced by combining two *PylT*$_{XXX}$ genes with distinct anticodon sequences. Ovaries were harvested from 15 females expressing *PylRS* and *PylT*$_{XXX}$ fed normal food containing **1** (10 mM), or in the control lane from 15 females not expressing *PylRS* and *PylT*$_{XXX}$ but fed normal food containing **1** (10 mM). Protein lysates were labeled with tetrazine-fluorophore conjugate **7** (4 μM, Figure S11). *PylT*$_{XXX}$ corresponds to variants of *PylT* bearing anticodon A = *PylT*$_{UGC}$ (Ala); S = *PylT*$_{GCU}$ (Ser), and M = *PylT*$_{CAU}$ (Met) or combinations bearing two variants of *PylT*$_{xxx}$, for example, AM = *PylT*$_{UGC}$ (Ala) and *PylT*$_{CAU}$ (Met). The data shown are from a gel in which intervening lanes have been removed (indicated by white space). The full gel is detailed in Figure S11.

(B) Proteome labeling from fly ovaries is further enhanced by combining three *PylT*$_{XXX}$ genes with distinct anticodon sequences. Experiment performed as in (A). The data shown are from a gel in which intervening lanes have been removed (indicated by white space). The full gel is detailed in Figure S11.

(C) SORT-E from the germ cells of *nos-vp16-GAL4*, *PylRS*, *PylT* (Ala, Met, Ser) fly ovaries. Ovaries from flies fed amino acid **1** or control flies not fed the amino acid were lysed. Lysates were labeled with **2** and tagged proteins captured with streptavidin beads, non-specifically bound proteins were washed away, and specifically captured proteins were eluted by cleavage of **2**. All steps were performed as described for *E. coli* without further optimization. Aliquots from the initial lysate, the final wash, and eluted fractions were analyzed by SDS-PAGE.

(D) Venn diagrams representing the number of proteins identified by MS from the SORT-E elution from flies fed **1** (gray) and the no-amino-acid control (blue).

more efficiently labeled than flies containing any one of these PylT$_{XXX}$ sequences alone (Figures 7A and S11). Moreover, we found that the proteomes of flies containing *PylT*$_{UGC}$ (Ala), *PylT*$_{CAU}$ (Met), *PylT*$_{GCU}$ (Ser) were more efficiently labeled than the proteomes of flies containing the most efficient pair of *PylT*$_{XXX}$ variants (Figures 7B and S11) and used these flies, henceforth referred to as *PylRS*, *PylT* (Ala, Met, Ser) flies, for further experiments.

nos-vp16-GAL4, *PylRS*, *PylT* (Ala, Met, Ser) flies were fed with or without the addition of **1** to their food to provide SORT-labeled flies and genetically matched control flies that were not labeled via SORT. The proteins from the ovaries of each group of flies were extracted and subjected to the TDB-mediated enrichment procedure described herein for *E. coli*, without further optimization. Using this approach we were able to selectively enrich the proteome from the germ cells of the ovaries of flies fed amino acid **1** (Figure 7C). Additional experiments suggested that using 2-fold more nos-vp16-GAL4, *PylRS*, *PylT* (Ala, Met, Ser) ovary lysate than *PylRS*/*PylT*$_{GCU}$ *E. coli* lysate as an input yielded comparable amounts of specifically eluted proteins (Figure S12). This suggests that the efficiency of SORT tagging in this fly line approaches that in *E. coli*. To further quantify the specificity of the approach for identifying labeled proteins from the fly ovary, we performed small-scale in-gel tryptic digests and LC-MS/MS on 2 μg of the eluted proteins. We identified 299 proteins in flies fed amino acid **1** and 17 proteins in control flies that were not fed the amino acid (Figure 7D and Table S3). The proteins shared between the two samples, on which we have statistically significant data, show amino acid-dependent enrichment (Figure S13). These data clearly demonstrate that SORT-E can be used to enrich tagged proteins from a multicellular system.

DISCUSSION

We have developed a TDB probe for the covalent capture of proteins containing **1**.

We have taken advantage of the site-specific incorporation of **1**, via genetic code expansion, to carefully characterize both the reaction of this probe with proteins containing **1** at specific sites and the reductive cleavage of the probe, ensuring that both steps are quantitative. We have demonstrated that proteomes labeled with **1** via SORT at diverse codons can be

efficiently enriched (SORT-E) and then identified, demonstrating that inverse electron-demand Diels-Alder reactions can be used for the enrichment and identification, in addition to the imaging and control, of biomolecules. We anticipate that our approach may be extended to the capture, enrichment, and identification of proteins and other biomolecules that may be labeled with strained, or otherwise activated, alkenes or alkynes.

We demonstrate that SORT-E does not preferentially identify proteins of most molecular weights, and that SORT-E shows a slight bias toward the identification of low-abundance proteins, which should aid their identification. We find that SORT-E at different codons leads to the enrichment of many proteins with different efficiencies. While it has previously been argued that labeling at methionine is sufficient to cover the proteome (Yuet and Tirrell, 2014), as all proteins contain an N-terminal methionine we find that numerous proteins are more effectively enriched and detected via labeling at a non-methionine codon. Our results also suggest that coverage may be increased by tagging the proteome using tRNAs that target several codons. Moreover, we demonstrate that SORT-E can be used to selectively enrich the proteomes, tagged in response to several codons, from specific cells in the fly.

Because azide/alkyne cycloadditions and cyclopropene/tetrazine cycloadditions are mutually orthogonal bio-orthogonal reactions (Sachdeva et al., 2014; Shih et al., 2014), it will be possible to label distinct biomolecules in a single sample with azides or alkynes and cyclopropenes, and selectively and independently enrich and identify each population of labeled biomolecules. Given the diversity of biomolecules that can now be labeled with azides and strained alkenes, this opens up many exciting new experimental possibilities.

We anticipate that the combination of SORT, which we have previously demonstrated can be used to label the proteomes of selected cells within a whole organism, and the enrichment strategies we have carefully characterized here will enable the identification of proteins expressed in specific cells at specific times in development, disease progression, and learning and memory. Indeed, we are currently using SORT-E, in an expanding set of multicellular systems, to define cell-specific proteomes.

SIGNIFICANCE

Well-characterized methods for tagging and enriching the proteomes of genetically targeted cells will provide a foundation for cell-specific proteomics in multicellular systems and whole animals. Such approaches are essential for defining how the distinct proteomes of cells in a body, or other multicellular system, carry out differentiated functions. Co-translational labeling of proteomes in *E. coli* and genetically targeted cells in the fly with a cyclopropene group, coupled with covalent capture via a tetrazine-biotin probe containing a cleavable linker, enables us to enrich labeled proteins from genetically targeted cells in an approach we term stochastic orthogonal recoding of translation with enrichment (SORT-E). Our approach allows the tagging of proteins in response to distinct codons. We demonstrate that tagging at distinct codons leads to different enrichments, suggesting that labeling at more than one codon enhances proteome coverage. We anticipate that SORT-E will enable the definition of cell-specific proteomes in animals during development, disease progression, and learning and memory.

EXPERIMENTAL PROCEDURES

Sources of chemicals, details of chemical synthesis and MS, data analysis, and computer code used for analysis are provided in Supplemental Experimental Procedures.

SORT-E in *E. coli*

SORT-E involves chemoselective labeling of proteomes tagged with **1** by TDB conjugate **2**, and enrichment and elution of SORT-E proteins.

For capture, enrichment, and elution of tagged proteins, 500 μl of cleared cell lysate (3.5–4 mg of 8 mg ml^{-1} lysate) was typically used. To 500 μl of cleared cell lysate, DTT (1 mM) was added and mixed with gentle vortexing, and the mixture was incubated at room temperature for 45 min. Iodoacetamide (5.5 mM) was added, mixed with gentle vortexing, then incubated for 30 min. **2** (5 μl, 20 μM final concentration, from a 2 mM stock in DMSO) was added, the reactions mixed by gentle vortexing, and the samples incubated in the dark overnight with end-over-end rotation (Hula mixer). 10-μl aliquots were taken at this stage as input sample for subsequent SDS-PAGE analysis. The samples were then diluted with PBS to a final volume of 5 ml and high-capacity streptavidin beads (150 μl of settled resin, pre-equilibrated in PBS, Thermo Scientific Streptavidin Agarose resin) added. The beads were incubated with end-over-end rotation (Hula mixer) for 1.5 hr and then collected by gravity filtration through a column (Poly-Prep chromatography column, Bio-Rad 731-1550). The beads were resuspended in urea buffer (500 μl, 8 M urea, 25 mM Tris, [pH 8]) and transferred to a smaller spin column (Mini Bio-spin chromatography column, Bio-Rad 731-1550). The beads were collected by mild centrifugation (1,000 rpm, 5-s pulse) and washed 2× urea buffer (500 μl) then 2 × 1% SDS in PBS (500 μl) with 15-min incubation times between each wash. The final wash acts as a control for the $Na_2S_2O_4$ specific elution; the beads were resuspended in 1% SDS in PBS (150 μl) and incubated at room temperature for 30 min with end-over-end rotation (Hula mixer). The beads collected by mild centrifugation (1,000 rpm, 5-s pulse) and the supernatant kept for subsequent analysis by SDS-PAGE. Specifically bound proteins were then eluted by resuspending the beads in 1% SDS in PBS supplemented with 25 mM $Na_2S_2O_4$ (150 μl) and incubating at room temperature for 30 min with end-over-end rotation (Hula mixer). The supernatant was collected by mild centrifugation (1,000 rpm, 5-s pulse) and analyzed by SDS-PAGE.

Fly Lines and Culture Conditions

All flies were grown at 25°C on standard Iberian medium. Flies were fed **1** by mixing dried yeast with a solution of **1** (10 mM) to form a paste. This paste was added as a supplement to the normal Iberian fly food for a minimum of 24 hr and the yeast was changed daily.

Double- and triple-sense codon lines were created by recombination using the original lines FT58 (A = $PylT_{UGC}$, Ala), FT60 (S = $PylT_{GCU}$, Ser), FT62 (L = $PylT_{CAG}$, Leu), and FT63 (M = $PylT_{CAU}$, Met) (Elliott et al., 2014b). Trans-heterozygous virgins were collected for each pairwise combination of sense codon (AS, FT58/FT60; AL, FT58/FT62; AM, FT58/FT63; SL, FT60/FT62; SM, FT60/FT63; and LM, FT62/FT63) and crossed to males of the third chromosome balancer stock *w*;;TM3/TM6. Potential recombinant males were identified based on eye color, and individuals were backcrossed to virgins of *w*;;TM3/TM6 to make a balanced stock. Recombinant lines were then screened by crossing to *nos-vp16-GAL4* virgins (Bloomington 4937) to create FT58-60/*nos-vp16-GAL4* (AS), FT58-62/*nos-vp16-GAL4* (AL), etc., and compared with the original single-sense codon lines FT58/*nos-vp16-GAL4*, FT60/*nos-vp16-GAL4*, FT62/*nos-vp16-GAL4*, and FT63/*nos-vp16-GAL4*. The females were fed 10 mM **1** for 24–48 hr, after which the ovaries were extracted from 15 females of the indicated genotype and labeled with 4 μM **7** for 2 hr as described by Elliott et al. (2014b).

Triple-sense codon lines were generated in a similar manner. In this case the double-sense codon lines were crossed to a different single-sense codon line to generate trans-heterozygotes with three different sense codons. In this case

not all combinations produced viable trans-heterozygotes and in some cases trans-heterozygotes were viable but gave no potential recombinant males. Successful combinations were FT58-63 (AM)/FT60 (S), FT60-63 (SM)/FT62 (L), and FT58-60 (AS)/FT63 (M), which generated the lines FT58-60-63 (ASM) and FT60-62-63 (SLM). These lines were screened by crossing to nos-vp16-GAL4 virgins and compared with the double-sense codon lines as described above (Elliott et al., 2014b).

SORT-E from *D. melanogaster*

Ovaries were dissected from 250 female flies of FT58-60-63/*nos-vp16-GAL4* (ASM), which had been fed normal food either supplemented with **1** (10 mM) or without **1**. The ovaries were homogenized into 8 M urea and 15 mM Tris (250 μl) and the resultant protein lysate clarified by filtration. A Bradford assay was used to determine the protein concentration. For SORT-E and subsequent MS, typically 7 mg of fly ovary protein was used and labeled with **2** (20 μM) in an identical procedure as described in the protocol for *E. coli* above.

SUPPLEMENTAL INFORMATION

Supplemental Information includes Supplemental Experimental Procedures, 13 figures, three tables, and Supplemental Program and can be found with this article online at http://dx.doi.org/10.1016/j.chembiol.2016.05.018.

AUTHOR CONTRIBUTIONS

T.S.E. performed all chemical and biochemical experiments with *E. coli*. A.B., F.M.T., and T.S.E. performed the biochemical experiments with *D. melanogaster*. T.S.E. and S.D.F. analyzed the data. J.W.C. and T.S.E. wrote the paper with input from all authors.

ACKNOWLEDGMENTS

This work was supported by the Medical Research Council, UK (MC_U105181009 and MC_UP_A024_1008, to J.W.C.). S.D.F. was supported by a King's College junior research fellowship. We are grateful to Mark Skehel and the MRC-LMB Mass spectrometry service for protein characterization and the LFQ-based proteomics, and to Kate Heesom at Bristol University for TMT label-based proteomics.

Received: March 23, 2016
Revised: May 13, 2016
Accepted: May 23, 2016
Published: July 21, 2016

REFERENCES

Agard, N.J., Prescher, J.A., and Bertozzi, C.R. (2004). A strain-promoted [3 + 2] azide-alkyne cycloaddition for covalent modification of biomolecules in living systems. J. Am. Chem. Soc. *126*, 15046–15047.

Agarwal, P., Beahm, B.J., Shieh, P., and Bertozzi, C.R. (2015). Systemic fluorescence imaging of zebrafish glycans with bioorthogonal chemistry. Angew. Chem. Int. Ed Engl. *54*, 11504–11510.

Asare-Okai, P.N., Agustin, E., Fabris, D., and Royzen, M. (2014). Site-specific fluorescence labelling of RNA using bio-orthogonal reaction of trans-cyclooctene and tetrazine. Chem. Commun. (Camb.) *50*, 7844–7847.

Bagert, J.D., van Kessel, J.C., Sweredoski, M.J., Feng, L., Hess, S., Bassler, B.L., and Tirrell, D.A. (2016). Time-resolved proteomic analysis of quorum sensing in. Chem. Sci. *7*, 1797–1806.

Bantscheff, M., Lemeer, S., Savitski, M.M., and Kuster, B. (2012). Quantitative mass spectrometry in proteomics: critical review update from 2007 to the present. Anal. Bioanal. Chem. *404*, 939–965.

Blackman, M.L., Royzen, M., and Fox, J.M. (2008). Tetrazine ligation: fast bioconjugation based on inverse-electron-demand Diels-Alder reactivity. J. Am. Chem. Soc. *130*, 13518–13519.

Borrmann, A., Milles, S., Plass, T., Dommerholt, J., Verkade, J.M., Wiessler, M., Schultz, C., van Hest, J.C., van Delft, F.L., and Lemke, E.A. (2012). Genetic encoding of a bicyclo[6.1.0]nonyne-charged amino acid enables fast cellular protein imaging by metal-free ligation. Chembiochem *13*, 2094–2099.

Busskamp, H., Batroff, E., Niederwieser, A., Abdel-Rahman, O.S., Winter, R.F., Wittmann, V., and Marx, A. (2014). Efficient labelling of enzymatically synthesized vinyl-modified DNA by an inverse-electron-demand Diels-Alder reaction. Chem. Commun. *50*, 10827–10829.

Chesarino, N.M., Hach, J.C., Chen, J.L., Zaro, B.W., Rajaram, M.V., Turner, J., Schlesinger, L.S., Pratt, M.R., Hang, H.C., and Yount, J.S. (2014). Chemoproteomics reveals Toll-like receptor fatty acylation. BMC Biol. *12*, 91.

Chin, J.W. (2014). Expanding and reprogramming the genetic code of cells and animals. Annu. Rev. Biochem. *83*, 379–408.

Cox, J., Hein, M.Y., Luber, C.A., Paron, I., Nagaraj, N., and Mann, M. (2014). Accurate proteome-wide label-free quantification by delayed normalization and maximal peptide ratio extraction, termed MaxLFQ. Mol. Cell Proteomics *13*, 2513–2526.

Cravatt, B.F., Wright, A.T., and Kozarich, J.W. (2008). Activity-based protein profiling: from enzyme chemistry to proteomic chemistry. Annu. Rev. Biochem. *77*, 383–414.

Denk, C., Svatunek, D., Filip, T., Wanek, T., Lumpi, D., Frohlich, J., Kuntner, C., and Mikula, H. (2014). Development of a (18)F-labeled tetrazine with favorable pharmacokinetics for bioorthogonal PET imaging. Angew. Chem. Int. Ed Engl. *53*, 9655–9659.

Devaraj, N.K., Weissleder, R., and Hilderbrand, S.A. (2008). Tetrazine-based cycloadditions: application to pretargeted live cell imaging. Bioconjug. Chem. *19*, 2297–2299.

Dieterich, D.C., Link, A.J., Graumann, J., Tirrell, D.A., and Schuman, E.M. (2006). Selective identification of newly synthesized proteins in mammalian cells using bioorthogonal noncanonical amino acid tagging (BONCAT). Proc. Natl. Acad. Sci. USA *103*, 9482–9487.

Dieterich, D.C., Lee, J.J., Link, A.J., Graumann, J., Tirrell, D.A., and Schuman, E.M. (2007). Labeling, detection and identification of newly synthesized proteomes with bioorthogonal non-canonical amino-acid tagging. Nat. Protoc. *2*, 532–540.

Dommerholt, J., van Rooijen, O., Borrmann, A., Guerra, C.F., Bickelhaupt, F.M., and van Delft, F.L. (2014). Highly accelerated inverse electron-demand cycloaddition of electron-deficient azides with aliphatic cyclooctynes. Nat. Commun. *5*, 5378.

Elliott, T.S., Bianco, A., and Chin, J.W. (2014a). Genetic code expansion and bioorthogonal labelling enables cell specific proteomics in an animal. Curr. Opin. Chem. Biol. *21*, 154–160.

Elliott, T.S., Townsley, F.M., Bianco, A., Ernst, R.J., Sachdeva, A., Elsasser, S.J., Davis, L., Lang, K., Pisa, R., Greiss, S., et al. (2014b). Proteome labeling and protein identification in specific tissues and at specific developmental stages in an animal. Nat. Biotechnol. *32*, 465–472.

Erdmann, I., Marter, K., Kobler, O., Niehues, S., Abele, J., Muller, A., Bussmann, J., Storkebaum, E., Ziv, T., Thomas, U., et al. (2015). Cell-selective labelling of proteomes in *Drosophila melanogaster*. Nat. Commun. *6*, 7521.

Hong, V., Presolski, S.I., Ma, C., and Finn, M.G. (2009). Analysis and optimization of copper-catalyzed azide-alkyne cycloaddition for bioconjugation. Angew. Chem. Int. Ed Engl. *48*, 9879–9883.

Kaya, E., Vrabel, M., Deiml, C., Prill, S., Fluxa, V.S., and Carell, T. (2012). A genetically encoded norbornene amino acid for the mild and selective modification of proteins in a copper-free click reaction. Angew. Chem. Int. Ed Engl. *51*, 4466–4469.

Kiick, K.L., Saxon, E., Tirrell, D.A., and Bertozzi, C.R. (2002). Incorporation of azides into recombinant proteins for chemoselective modification by the Staudinger ligation. Proc. Natl. Acad. Sci. USA *99*, 19–24.

Kleiner, R.E., Verma, P., Molloy, K.R., Chait, B.T., and Kapoor, T.M. (2015). Chemical proteomics reveals a gammaH2AX-53BP1 interaction in the DNA damage response. Nat. Chem. Biol. *11*, 807–814.

Kurra, Y., Odoi, K.A., Lee, Y.J., Yang, Y., Lu, T., Wheeler, S.E., Torres-Kolbus, J., Deiters, A., and Liu, W.R. (2014). Two rapid catalyst-free click reactions for

in vivo protein labeling of genetically encoded strained alkene/alkyne functionalities. Bioconjug. Chem. *25*, 1730–1738.

Lang, K., and Chin, J.W. (2014a). Bioorthogonal reactions for labeling proteins. ACS Chem. Biol. *9*, 16–20.

Lang, K., and Chin, J.W. (2014b). Cellular incorporation of unnatural amino acids and bioorthogonal labeling of proteins. Chem. Rev. *114*, 4764–4806.

Lang, K., Davis, L., Torres-Kolbus, J., Chou, C., Deiters, A., and Chin, J.W. (2012a). Genetically encoded norbornene directs site-specific cellular protein labelling via a rapid bioorthogonal reaction. Nat. Chem. *4*, 298–304.

Lang, K., Davis, L., Wallace, S., Mahesh, M., Cox, D.J., Blackman, M.L., Fox, J.M., and Chin, J.W. (2012b). Genetic Encoding of bicyclononynes and trans-cyclooctenes for site-specific protein labeling in vitro and in live mammalian cells via rapid fluorogenic Diels-Alder reactions. J. Am. Chem. Soc. *134*, 10317–10320.

Liu, C.C., and Schultz, P.G. (2010). Adding new chemistries to the genetic code. Annu. Rev. Biochem. *79*, 413–444.

Mahdavi, A., Hamblin, G.D., Jindal, G.A., Bagert, J.D., Dong, C., Sweredoski, M.J., Hess, S., Schuman, E.M., and Tirrell, D.A. (2016). Engineered aminoacyl-tRNA synthetase for cell-selective analysis of mammalian protein synthesis. J. Am. Chem. Soc. *138*, 4278–4281.

Mancuso, L., Jurjens, G., Hermane, J., Harmrolfs, K., Eichner, S., Fohrer, J., Collisi, W., Sasse, F., and Kirschning, A. (2013). Bioreduction of aryl azides during mutasynthesis of new ansamitocins. Org. Lett. *15*, 4442–4445.

McAlister, G.C., Huttlin, E.L., Haas, W., Ting, L., Jedrychowski, M.P., Rogers, J.C., Kuhn, K., Pike, I., Grothe, R.A., Blethrow, J.D., et al. (2012). Increasing the multiplexing capacity of TMTs using reporter ion isotopologues with isobaric masses. Anal. Chem. *84*, 7469–7478.

Ngo, J.T., and Tirrell, D.A. (2011). Noncanonical amino acids in the interrogation of cellular protein synthesis. Acc. Chem. Res. *44*, 677–685.

Ngo, J.T., Schuman, E.M., and Tirrell, D.A. (2013). Mutant methionyl-tRNA synthetase from bacteria enables site-selective N-terminal labeling of proteins expressed in mammalian cells. Proc. Natl. Acad. Sci. USA *110*, 4992–4997.

Nikic, I., Plass, T., Schraidt, O., Szymanski, J., Briggs, J.A., Schultz, C., and Lemke, E.A. (2014). Minimal tags for rapid dual-color live-cell labeling and super-resolution microscopy. Angew. Chem. Int. Ed Engl. *53*, 2245–2249.

Patterson, D.M., Nazarova, L.A., Xie, B., Kamber, D.N., and Prescher, J.A. (2012). Functionalized cyclopropenes as bioorthogonal chemical reporters. J. Am. Chem. Soc. *134*, 18638–18643.

Patterson, D.M., Jones, K.A., and Prescher, J.A. (2014). Improved cyclopropene reporters for probing protein glycosylation. Mol. Biosyst. *10*, 1693–1697.

Plass, T., Milles, S., Koehler, C., Szymanski, J., Mueller, R., Wiessler, M., Schultz, C., and Lemke, E.A. (2012). Amino acids for Diels-Alder reactions in living cells. Angew. Chem. Int. Ed Engl. *51*, 4166–4170.

Prescher, J.A., and Bertozzi, C.R. (2006). Chemical technologies for probing glycans. Cell *126*, 851–854.

Pyka, A.M., Domnick, C., Braun, F., and Kath-Schorr, S. (2014). Diels-Alder cycloadditions on synthetic RNA in mammalian cells. Bioconjug. Chem. *25*, 1438–1443.

Rieder, U., and Luedtke, N.W. (2014). Alkene-tetrazine ligation for imaging cellular DNA. Angew. Chem. Int. Ed Engl. *53*, 9168–9172.

Sachdeva, A., Wang, K., Elliott, T., and Chin, J.W. (2014). Concerted, rapid, quantitative, and site-specific dual labeling of proteins. J. Am. Chem. Soc. *136*, 7785–7788.

Selvaraj, R., Giglio, B., Liu, S., Wang, H., Wang, M., Yuan, H., Chintala, S.R., Yap, L.P., Conti, P.S., Fox, J.M., et al. (2015). Improved metabolic stability for 18F PET probes rapidly constructed via tetrazine trans-cyclooctene ligation. Bioconjug. Chem. *26*, 435–442.

Shih, H.W., Kamber, D.N., and Prescher, J.A. (2014). Building better bioorthogonal reactions. Curr. Opin. Chem. Biol. *21*, 103–111.

Smeekens, J.M., Chen, W., and Wu, R. (2015). Mass spectrometric analysis of the cell surface N-glycoproteome by combining metabolic labeling and click chemistry. J. Am. Soc. Mass Spectrom. *26*, 604–614.

Szychowski, J., Mahdavi, A., Hodas, J.J., Bagert, J.D., Ngo, J.T., Landgraf, P., Dieterich, D.C., Schuman, E.M., and Tirrell, D.A. (2010). Cleavable biotin probes for labeling of biomolecules via azide-alkyne cycloaddition. J. Am. Chem. Soc. *132*, 18351–18360.

Thompson, A., Schafer, J., Kuhn, K., Kienle, S., Schwarz, J., Schmidt, G., Neumann, T., Johnstone, R., Mohammed, A.K., and Hamon, C. (2003). Tandem mass tags: a novel quantification strategy for comparative analysis of complex protein mixtures by MS/MS. Anal. Chem. *75*, 1895–1904.

Tsai, Y.H., Essig, S., James, J.R., Lang, K., and Chin, J.W. (2015). Selective, rapid and optically switchable regulation of protein function in live mammalian cells. Nat. Chem. *7*, 554–561.

Uttamapinant, C., Howe, J.D., Lang, K., Beranek, V., Davis, L., Mahesh, M., Barry, N.P., and Chin, J.W. (2015). Genetic code expansion enables live-cell and super-resolution imaging of site-specifically labeled cellular proteins. J. Am. Chem. Soc. *137*, 4602–4605.

Vanbeselaere, J., Chang, L.Y., Harduin-Lepers, A., Fabre, E., Yamakawa, N., Slomianny, C., Biot, C., Khoo, K.H., and Guerardel, Y. (2012). Mapping the expressed glycome and glycosyltransferases of zebrafish liver cells as a relevant model system for glycosylation studies. J. Proteome Res. *11*, 2164–2177.

Verhelst, S.H., Fonovic, M., and Bogyo, M. (2007). A mild chemically cleavable linker system for functional proteomic applications. Angew. Chem. Int. Ed Engl. *46*, 1284–1286.

Wang, Q., Chan, T.R., Hilgraf, R., Fokin, V.V., Sharpless, K.B., and Finn, M.G. (2003). Bioconjugation by copper(I)-catalyzed azide-alkyne [3 + 2] cycloaddition. J. Am. Chem. Soc. *125*, 3192–3193.

Wang, M., Weiss, M., Simonovic, M., Haertinger, G., Schrimpf, S.P., Hengartner, M.O., and von Mering, C. (2012). PaxDb, a database of protein abundance averages across all three domains of life. Mol. Cell Proteomics *11*, 492–500.

Wang, M., Herrmann, C.J., Simonovic, M., Szklarczyk, D., and von Mering, C. (2015). Version 4.0 of PaxDb: protein abundance data, integrated across model organisms, tissues, and cell-lines. Proteomics *15*, 3163–3168.

Werner, T., Becher, I., Sweetman, G., Doce, C., Savitski, M.M., and Bantscheff, M. (2012). High-resolution enabled TMT 8-plexing. Anal. Chem. *84*, 7188–7194.

Yang, Y.Y., Grammel, M., Raghavan, A.S., Charron, G., and Hang, H.C. (2010). Comparative analysis of cleavable azobenzene-based affinity tags for bioorthogonal chemical proteomics. Chem. Biol. *17*, 1212–1222.

Yang, J., Seckute, J., Cole, C.M., and Devaraj, N.K. (2012). Live-cell imaging of cyclopropene tags with fluorogenic tetrazine cycloadditions. Angew. Chem. Int. Ed Engl. *51*, 7476–7479.

Yuet, K.P., and Tirrell, D.A. (2014). Chemical tools for temporally and spatially resolved mass spectrometry-based proteomics. Ann. Biomed. Eng. *42*, 299–311.

Yuet, K.P., Doma, M.K., Ngo, J.T., Sweredoski, M.J., Graham, R.L., Moradian, A., Hess, S., Schuman, E.M., Sternberg, P.W., and Tirrell, D.A. (2015). Cell-specific proteomic analysis in *Caenorhabditis elegans*. Proc. Natl. Acad. Sci. USA *112*, 2705–2710.

Zhang, M.M., Wu, P.Y., Kelly, F.D., Nurse, P., and Hang, H.C. (2013). Quantitative control of protein S-palmitoylation regulates meiotic entry in fission yeast. PLoS Biol. *11*, e1001597.

Zheng, T., Jiang, H., and Wu, P. (2013). Single-stranded DNA as a cleavable linker for bioorthogonal click chemistry-based proteomics. Bioconjug. Chem. *24*, 859–864.

Cell Chemical Biology, Volume *23*

Supplemental Information

Tagging and Enriching Proteins Enables Cell-Specific Proteomics

Thomas S. Elliott, Ambra Bianco, Fiona M. Townsley, Stephen D. Fried, and Jason W. Chin

I. Supplemental Figures

Figure S1. Intact protein spectra prior to deconvolution

Figure S2. MS2 spectra of tryptic peptides

Figure S3. Fluorescence imaging of SORT-M

Figure S4. Quantification of SORT-E protein

Figure S5. Dilution of SORT-E input for enrichment threshold

Figure S6. SORT-E LFQ ratios and errors

Figure S7. SORT-E efficiently extracts low abundance proteins

Figure S8. Quantitation of SORT-E for TMT

Figure S9. SORT-E and codon abundance model

Figure S10. SORT-E and SASA model

Figure S11. SORT-M in *D. melanogaster* with different PylT variants

Figure S12. SORT-M and SORT-E in *D. melanogaster* comparison with *E. Coli*

Figure S13. SORT-E in *D. melanogaster* protein quantification

Table S1. Protein identities and LFQ of SORT-E from *E. coli*

Table S2. Protein identities and quantification of SORT-E via 9-Plex TMT

Table S3. Protein identities and LFQ of SORT-E from *D. Melanogaster*

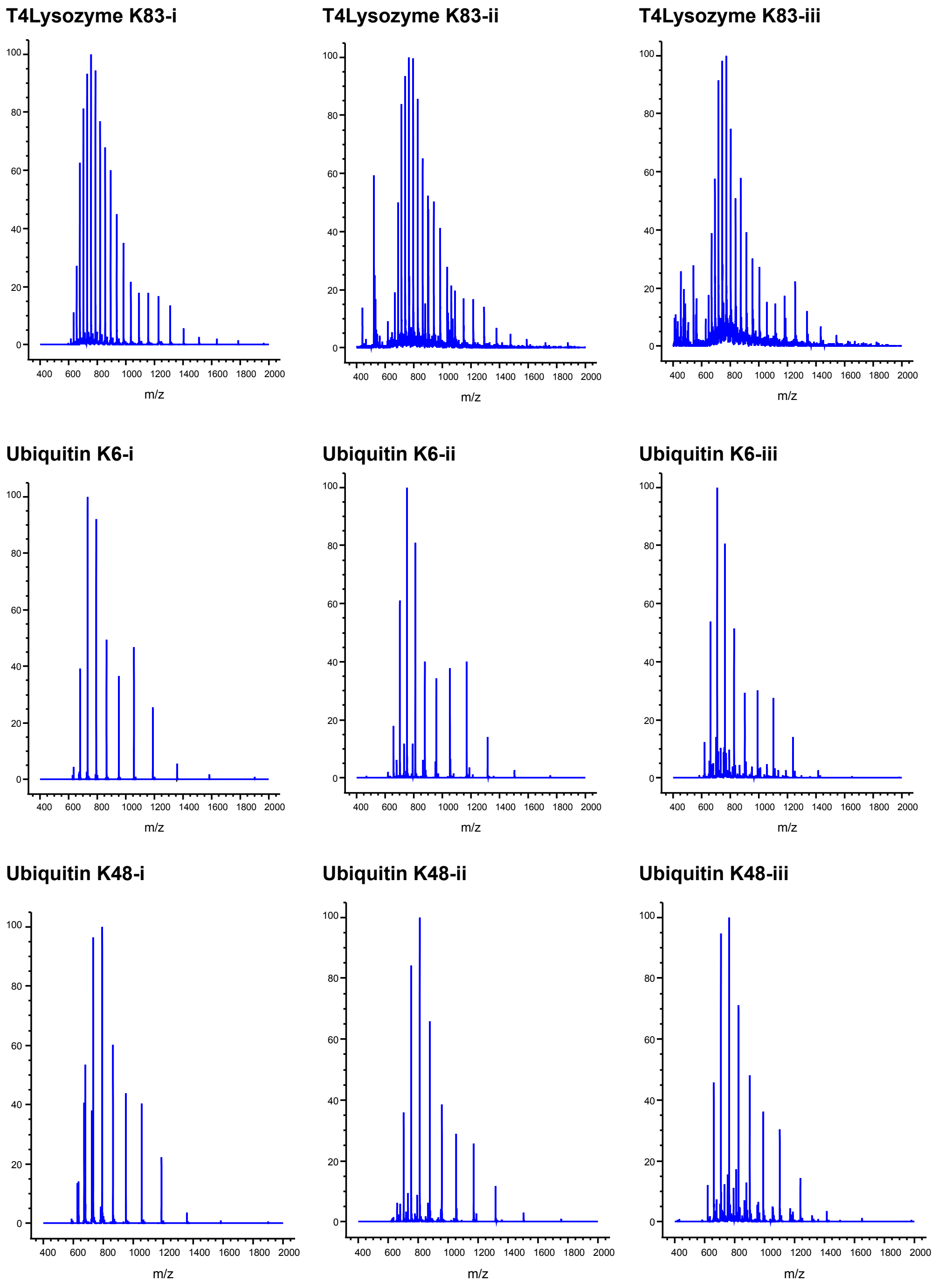

Figure S1 (related to Figure 3). Raw mass spectra for intact T4 lysozyme (K-83-**1**)-His6 (top row), ubiquitin (K-6-**1**)-His6 (middle row), and ubiquitin (K-48-**1**)-His6 (bottom row), as pure proteins (i), TDB conjugates (ii), and cleaved dipyridiyl-diaza cycloaddition products (iii). Spectra correspond to the deconvolved masses shown in **Figure 3**.

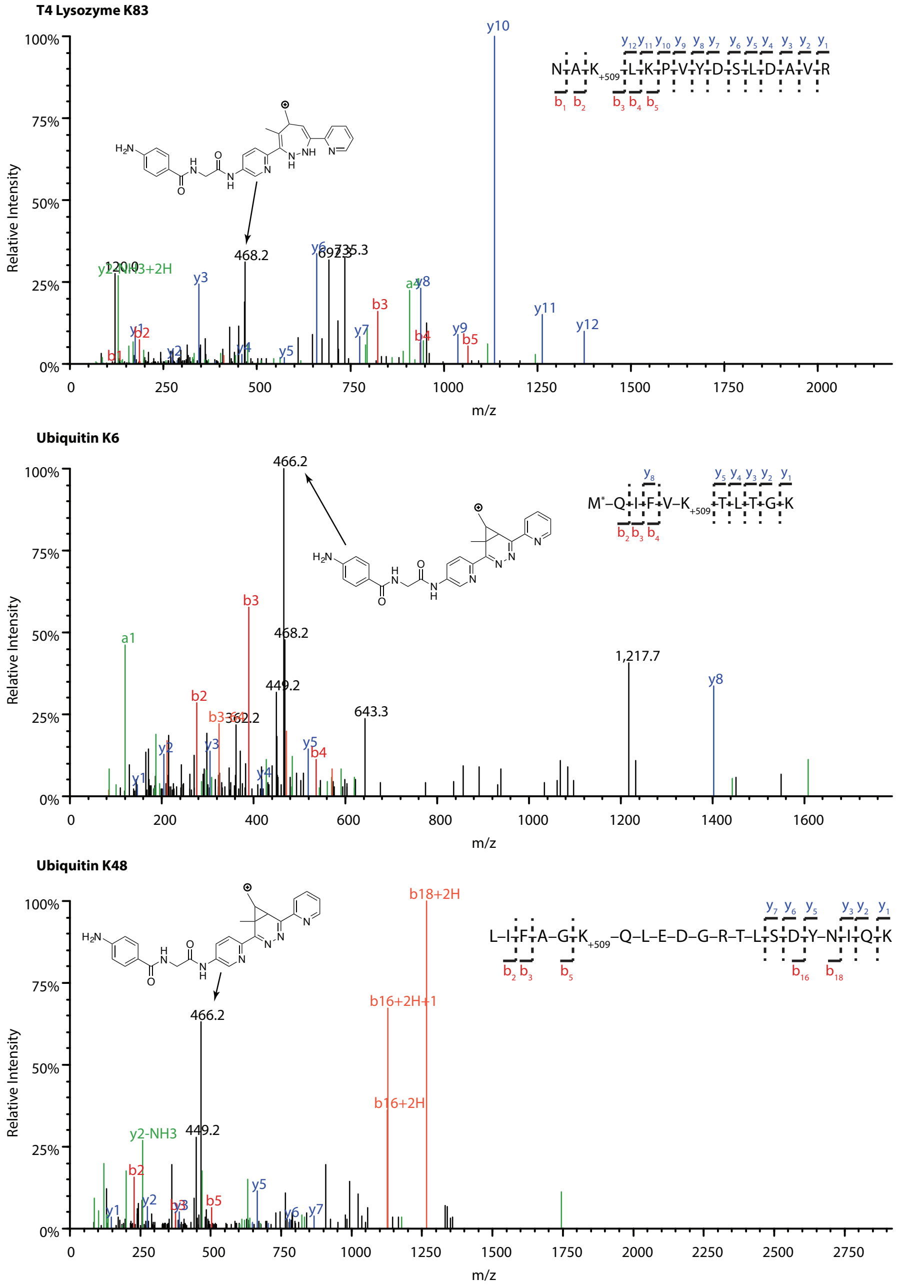

Figure S2 (related to Figure 3). MS2 spectra of tryptic peptides derived from species iii of T4 lysozyme (K-83-**1**)-His6 (top), ubiquitin (K-6-**1**)-His6 (middle), and ubiquitin (K-48-**1**)-His6 (bottom). The spectra confirm the presence of the dipyridiyl-diaza cycloaddition product at the correct site in each of the proteins. In the case of T4 Lysozyme, possible over-reduction of the modified side chain is observed, giving rise to a fragment peak 2 Da higher in mass.

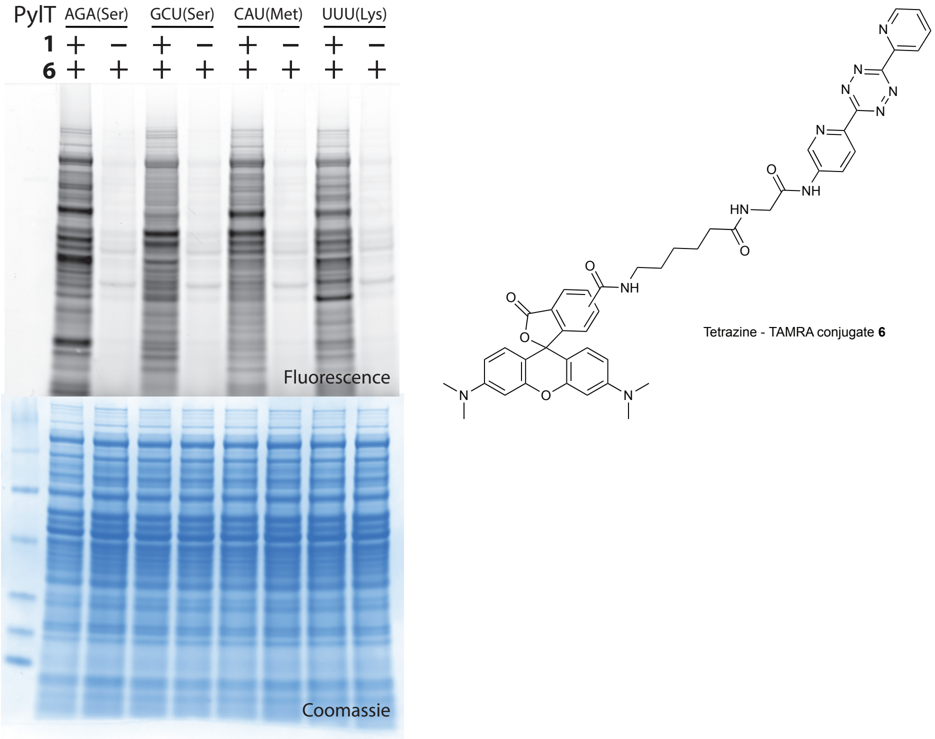

Figure S3 (related to Figure 4). *E. coli* expressing PylRS and either $PyltRNA_{AGA}$, $PyltRNA_{GCU}$, $PyltRNA_{CAU}$, or $PyltRNA_{UUU}$ were grown for 4 h at 37 ˚C – either with or without **1** (0.1 mM). Lysates were labelled with tetrazine-fluorophore conjugate, **6** (4 μM) for 4h, resolved by SDS-PAGE, and then analysed by Coomassie stain (bottom) or in-gel fluorescence (top). As based on the Coomassie image, all eight samples were identically loaded, but fluorescence labeling is strictly **1**-dependent for any tRNA anticodon used.

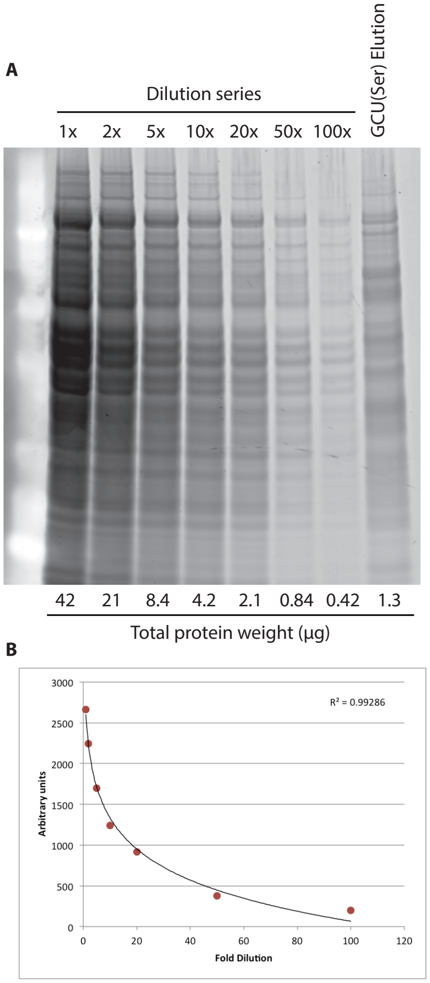

Figure S4 (related to Figure 4). Quantification of eluted protein from enrichment with **2**. **A.** SDS-PAGE gel analysis, lanes 1-7 loaded with serial dilutions of a lysate of known concentration from 42-0.42 µg of loaded protein (determined by Bradford assay). In lane 8 an aliquot of SORT-E proteins eluted from enrichment of GCU(Ser) lysate is loaded. Densitometry analysis of the lanes indicate that 1.3 µg of protein was loaded into lane 8. **B.** Standard curve generated from lane densitometry.

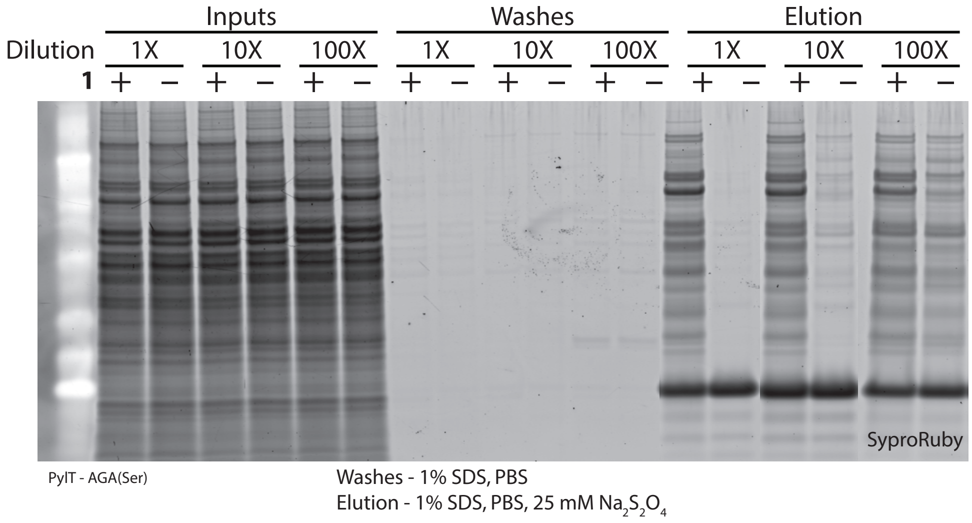

Figure S5 (related to Figure 4). *E. coli* expressing PylRS and $PyltRNA_{AGA}$ were grown for 4 h at 37 ˚C – either with or without **1** (0.1 mM). Next, lysates were either not diluted, diluted 10-fold, or diluted 100-fold with lysates from cultures that lacked **1**. Lysates were labelled with **2**, tagged proteins were captured with streptavidin beads, non-specifically bound proteins were washed away, and specifically captured proteins were eluted by cleavage of **2**. Aliquots from the initial lysate, the final wash, and eluted fraction were analysed by SDS-PAGE. In the case of the 10-fold and 100-fold diluted samples, 10-fold and 100-fold more input was used, so that the total amount of labelled protein is comparable in all lanes, but the concentration of labelled proteins with respect to non-labelled is lower. At 10-fold dilution, the elution profile is similar. At 100-fold dilution, non-specific reactions of **2** with unlabeled proteins begin to match the level of specific capture.

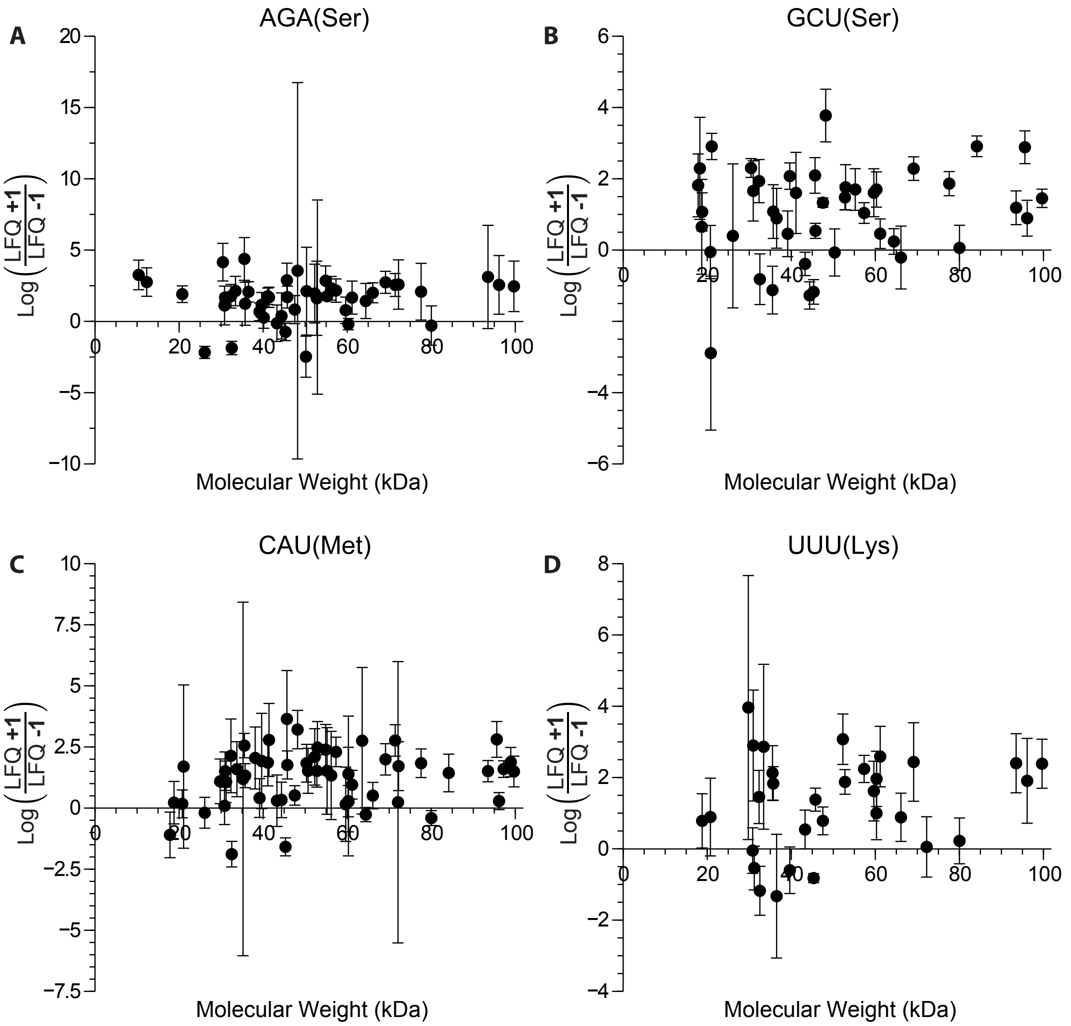

Figure S6 (related to Figure 4). The subset of proteins that are identified in both the SORT-E samples and controls (blue circles in **Figure 4** panels (B, D, F, G) from the main text are enriched, as judged by their LFQ values, in the SORT-E samples.

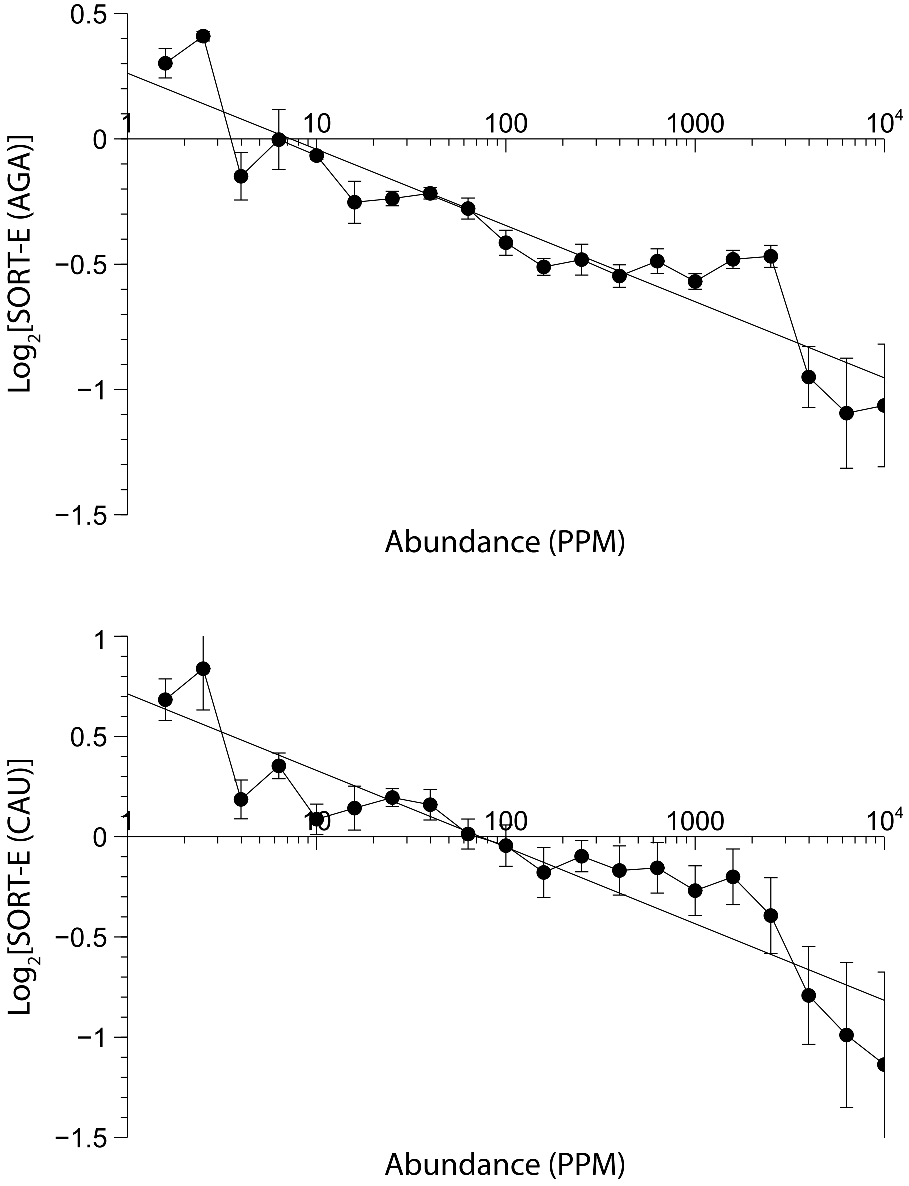

Figure S7 (related to Figure 5). (A, B) SORT-E enriches lower abundance proteins more efficiently than higher abundance proteins. The graphs show the log of the ratio of the relative abundance for proteins detected in SORT-E ((A) SORT-E (AGA, Ser); (B) SORT-E (CAU, Met)) to the relative abundance prior to enrichment. Each point corresponds to an average over all proteins in a given range of abundance, as defined in the PAX database (Wang et al., 2015; Wang et al., 2012). Error bars correspond to std. devs across biological triplicates. For SORT-E (AGA, Ser): $y = -0.132\ln(x) + 0.622; R^2 = 0.86$. For SORT-E (CAU, Met): $y = -0.166\ln(x) + 0.713; R^2 = 0.85$.

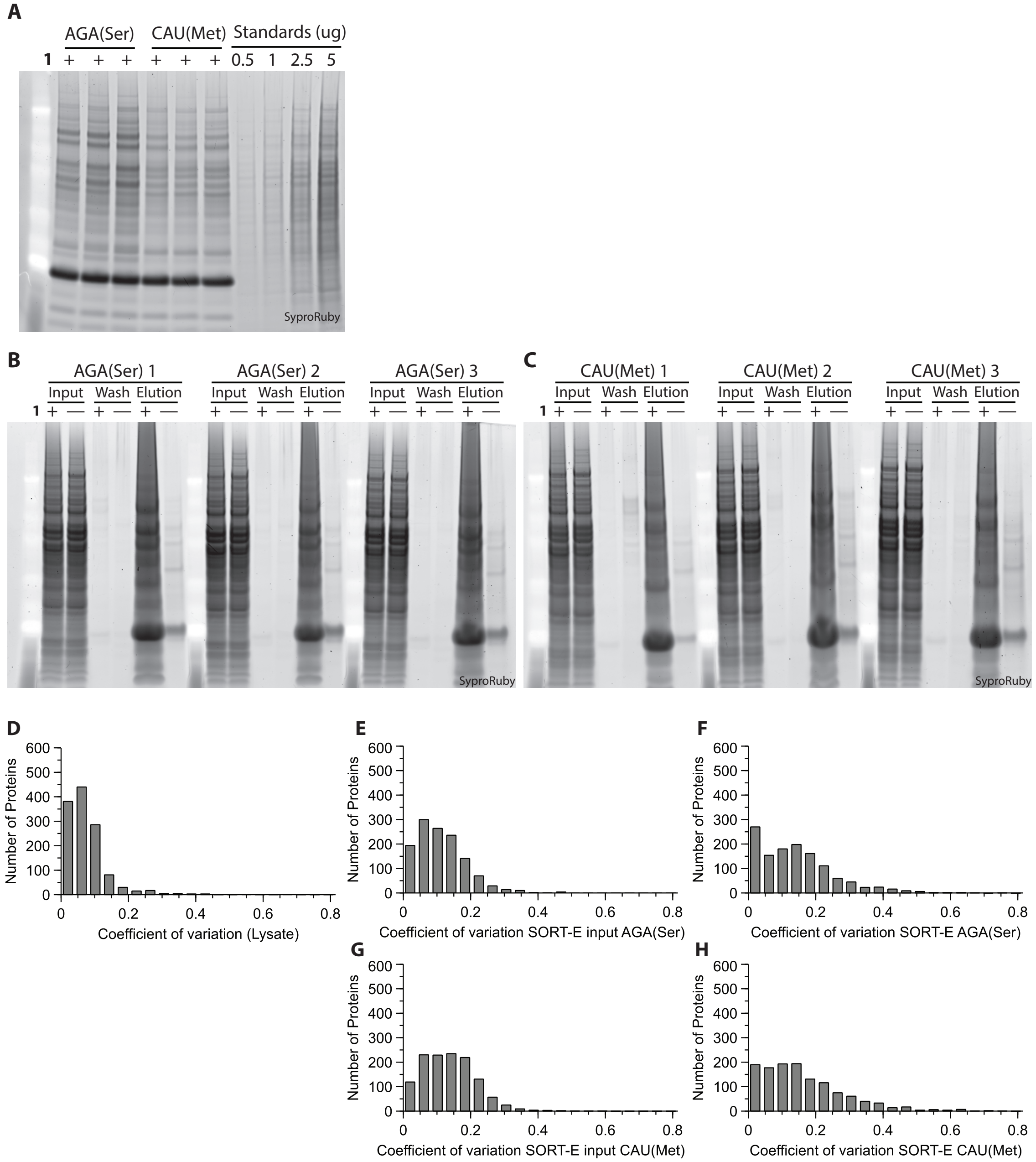

Figure S8 (related to Figure 6). (A) SDS-PAGE gel analysis for the quantification of eluted protein from SORT-E AGA(Ser) and SORT-E CAU(Met) used for TMT analysis, lanes 1-6 loaded with an aliquot of the combined eluent, lanes 7-10 loaded with serial dilutions of a lysate of know concentration form 0.5-5 µg of loaded protein (determined by Bradford assay). Lane densitometry calculations reveal 109 µg, 116 µg and 142 µg of SORT-E AGA(Ser) protein yield in the total 250 µL eluents. And 102 µg, 98 µg and 106 µg of SORT-E CAU(Met) protein yield in the total 250 µL eluents.

(B, C) Aliquots from the initial lysate, the final wash, and eluted fractions for each biological replicate of the SORT-E TMT experiment were analysed by SDS-PAGE.

(D, E, F, G, H) Histograms showing the coefficients of variation (CV) for protein abundances from three biological replicates. Protein abundances, as based on relative TMT ion counts, are very reproducible for all conditions studied. (D) The average CV for proteins from unmodified *E. coli* lysates is 0.075. (E, G) The average CV for proteins from SORT-E AGA(Ser) and CAU(Met) input proteins are 0.113 and 0.137, respectively. (F, H) The average CV from SORT-E AGA(Ser) and CAU(Met) proteins are 0.141 and 0.161, respectively.

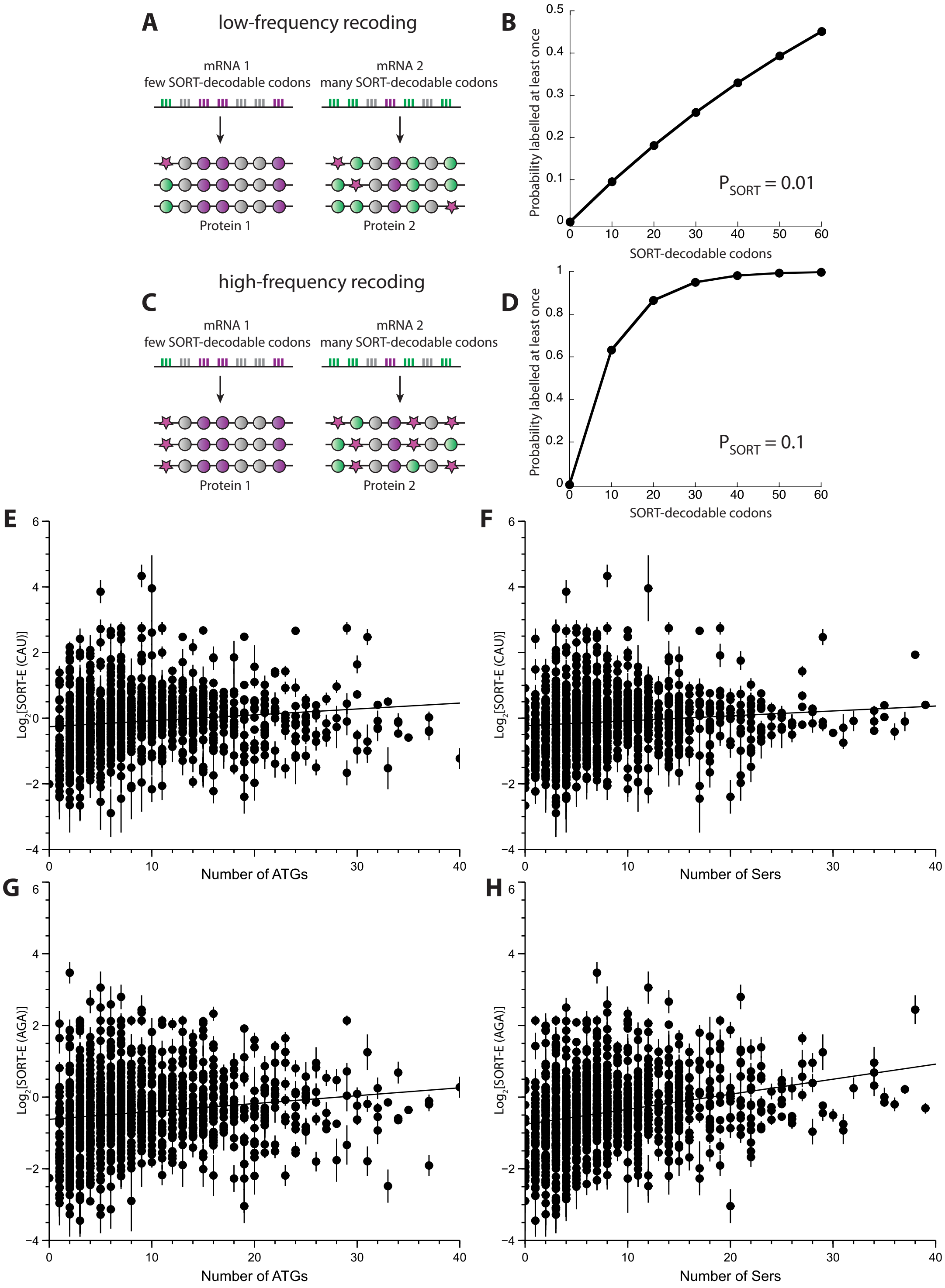

Figure S9 (related to Figure 6). (A, B) If stochastic recoding of sense codons were a rare event (P_{SORT} is small) , a protein that has more occurrences of the recodable codons has a higher likelihood of getting labeled, as based on Poisson statistics. (C, D) If stochastic recoding is relatively frequent, then a protein with more recodable codons will be labeled more times, but the likelihood of getting one label saturates quickly with

respect to the number of recodable codons. (E, F, G, H) The number of cognate codons in a given protein that can be decoded by the PylT variant only weakly explains the variance in the SORT-E pull-down efficiencies across proteins, though the correlation is stronger for serine than for methionine. It is reasonable that P_{SORT} would be lower for serine because the intracellular $\mathrm{tRNA}_{\mathrm{Ser}}$ concentration is ca. 4-fold greater than that of $\mathrm{tRNA}_{\mathrm{Met}}$ (Dong et al., 1996). The graphs show the log of the ratio of the relative abundance for proteins detected in SORT-E to the relative abundance prior to enrichment plotted against codons counts (where "Sers" includes UCU, UCA, and UCC). The least-square regression lines have the following parameters: (E) slope $= 0.018, R^2 = 0.019, p = 10^{-6}$. (F) slope $= 0.015, R^2 = 0.012, p = 10^{-4}$. (G) slope$= 0.022, R^2 = 0.023, p = 10^{-7}$. (H) slope $= 0.042, R^2 = 0.076, p = 10^{-23}$. These results are more consistent with high-frequency recoding (model C) than low-frequency recoding (model A). However this model does not take into account the efficiency with which tagged proteins may be captured.

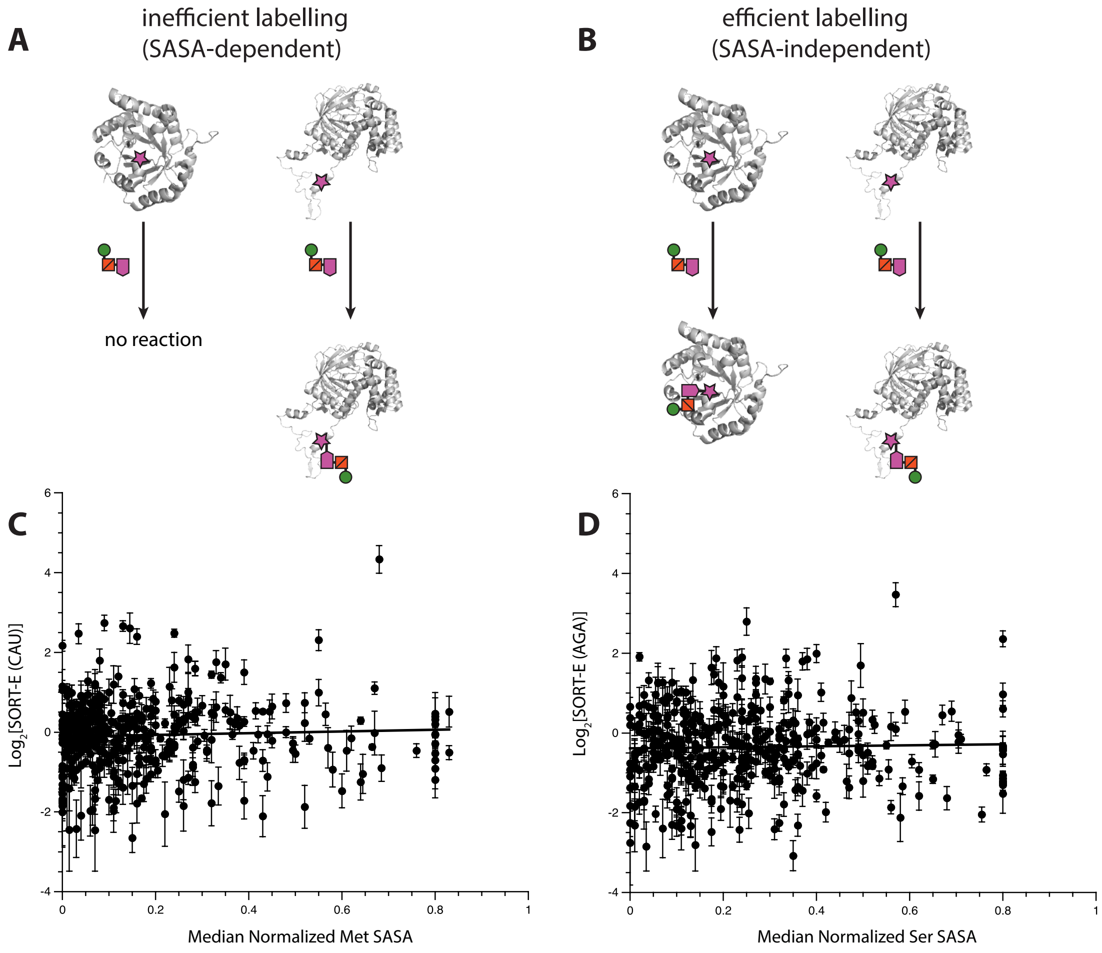

Figure S10 (related to Figure 6). (A, B) Proteins that have incorporated **1** are labeled to a tetrazine diazobenzene biotin compound, enabling them to be captured on streptavidin-coated beads. The efficiency of labeling could be affected by the accessibility of the positions in the protein where **1** can be incorporated, especially if labeling chemistry were slow and inefficient. (C, D) The median solvent-accessible surface area (SASA) of a SORT-decodable site in a given protein explains almost no variation in the SORT-E pull-down efficiencies across proteins. Normalized SASAs were calculated in PyMol for all *E. coli* proteins for which an x-ray structure was available in the PDB, and for each protein, the median SASA across all residues that could be replaced with **1** was taken. For (C), $R^2 = 0.002$; for (D), $R^2 = 0.0006$. These observations are consistent with the labeling chemistry being very efficient, and therefore not very hindered by protein structure.

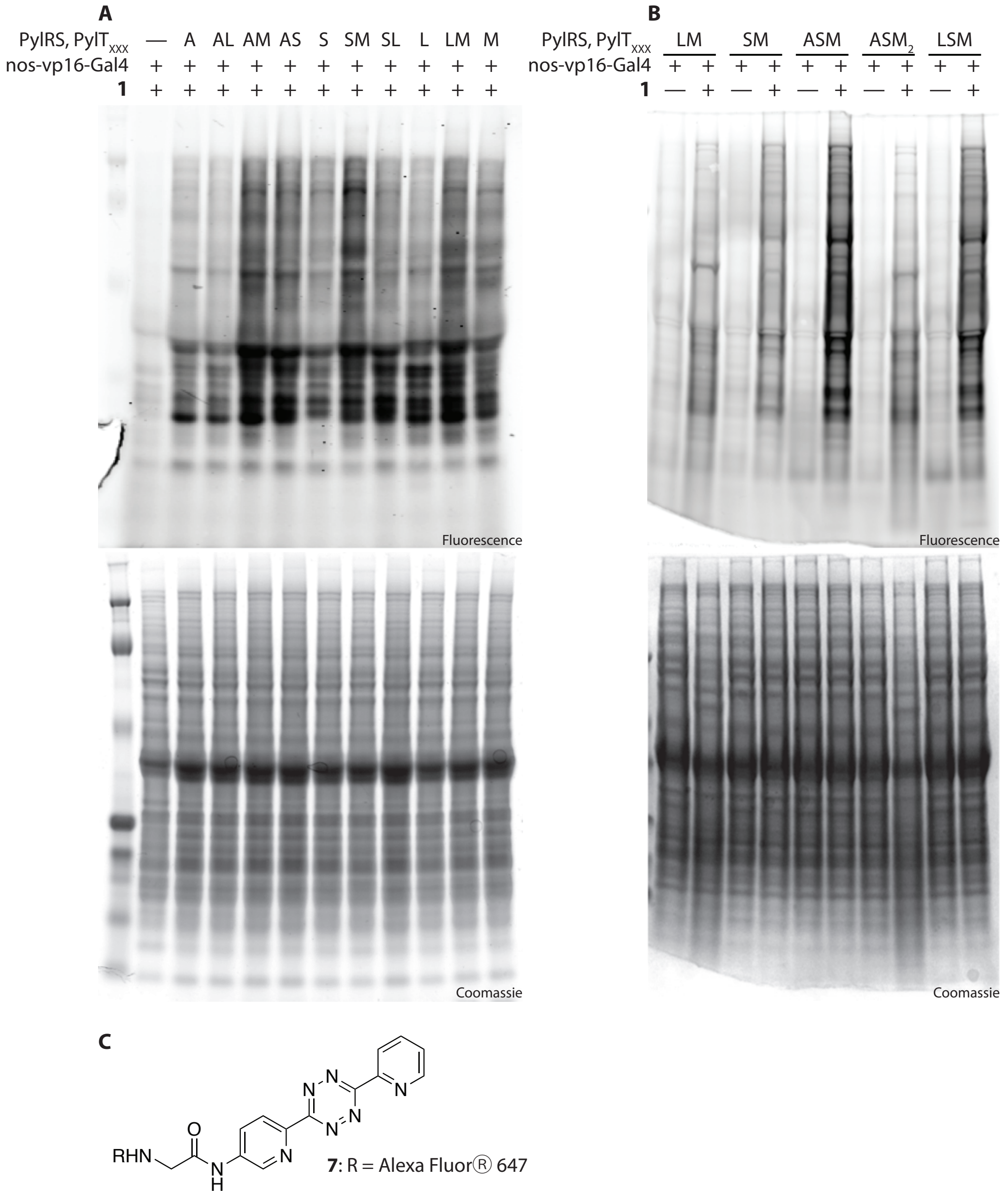

Figure S11 (related to Figure 7). (A) SDS-PAGE analysis of SORT-M labeling of ovary proteomes from *D. melanogaster*. Ovaries were harvested from 15 females expressing PylRS and $PylT_{XXX}$ fed normal food containing **1** (10 mM), or in the control lane from 15 females not expressing PylRS and $PylT_{XXX}$ but fed normal food containing **1** (10 mM). The protein lysates were labeled with tetrazine fluorophore conjugate **7** (4 µM) (Elliott et al., 2014). $PylT_{XXX}$ corresponds to variants of PylT bearing anti codon A = $PylT_{UGC}$ (Ala) or S = $PylT_{GCU}$ (Ser) or L = $PylT_{CAG}$ (Leu) or M = $PylT_{CAU}$ (Met) or combinations bearing two variants of $PylT_{xxx}$ for example AM = $PylT_{UGC}$ (Ala) and $PylT_{CAU}$ (Met).

(B) SDS-PAGE analysis of SORT-M labeling of ovary proteomes from *D. melanogaster*. Ovaries were harvested from 15 females expressing PylRS and $PylT_{xxx}$ fed either normal food containing **1** (10 mM) or normal food without **1**. The protein lysates were labeled with tetrazine fluorophore conjugate **7** (4 μM) (Elliott et al., 2014). Here $PylT_{xxx}$ corresponds to flies bearing combinations of two or three PylT variants for example LM = $PylT_{CAG}$ (Leu) and $PylT_{CAU}$ (Met); ASM = $PylT_{UGC}$ (Ala) and $PylT_{GCU}$ (Ser) and $PylT_{CAU}$ (Met).

(C) Structure of tetrazine fluorophore conjugate, **7** (Elliott et al., 2014).

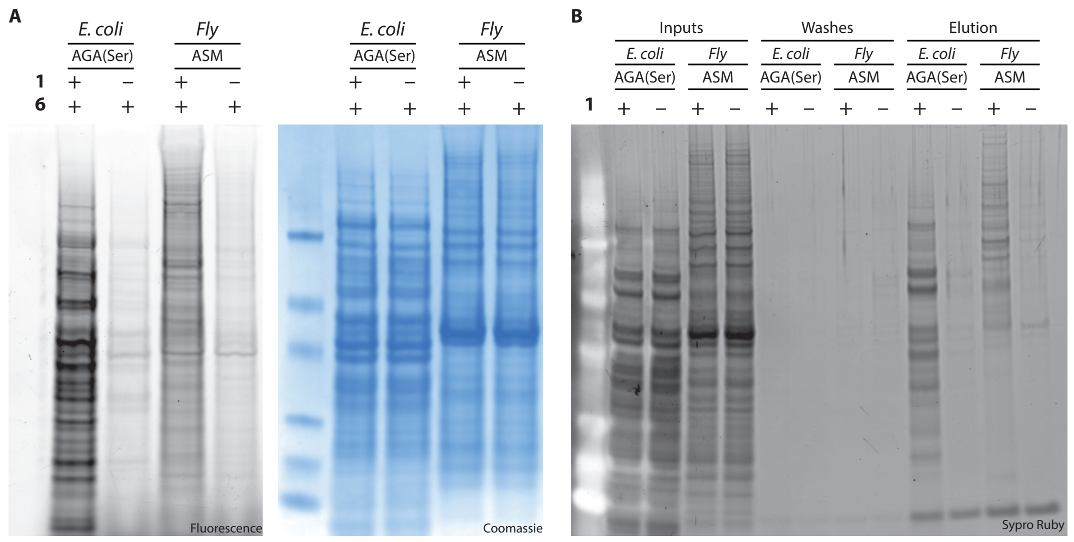

Figure S12 (related to Figure 7). (A) A representative SDS-PAGE analysis comparing the efficiency of SORT-M from *E. coli* cells to SORT-M from *D. melanogaster* ovaries. Equal quantities of protein lysate from *E. coli*, bearing PylRS and $PylT_{AGA}$ grown in LB supplemented with **1** (0.1 mM) or grown in LB without **1**; and protein lystate from *D. melanogaster*, bearing PylRS and three varients of PylT ($PylT_{UGC}$, $PylT_{GCU}$ and $PylT_{CAU}$), fed either normal food supplemented with **1** (10 mM) or normal food without **1**; were labeled with tetrazine fluorophore conjugate **6** (4 µM) (Lang et al., 2012). Densitometry calculations on fluorescence gels of three independent replicates show that *E. coli* proteome labeling is only 2.13±0.75 times more efficient than germline cell labeling. (B) SDS-PAGE analysis comparing the efficiency of SORT-E from *E. coli* cells to SORT-E from *D. melanogaster* ovaries. 3.5 mg of protein lysate from *E. coli*, bearing PylRS and $PylT_{AGA}$ grown in LB supplemented with **1** (0.1 mM) or grown in LB without **1**; and 7 mg of ovary protein lystate from *D. melanogaster*, bearing PylRS and three varients of PylT ($PylT_{UGC}$, $PylT_{GCU}$ and $PylT_{CAU}$), fed either normal food supplemented with **1** (10 mM) or normal food without **1**; were labeled with TDB probe **2** (20 µM) and subjected to the same SORT-E enrichment protocol. SDS-PAGE analysis of the input material, the final wash step, and the elution with $Na_2S_2O_4$ show comparable protein enrichment between *E. coli* and fly, in both an amino acid and $Na_2S_2O_4$ dependent manner. Indicating that germline cell labeling efficiency approaches that of *E. coli* cell labeling.

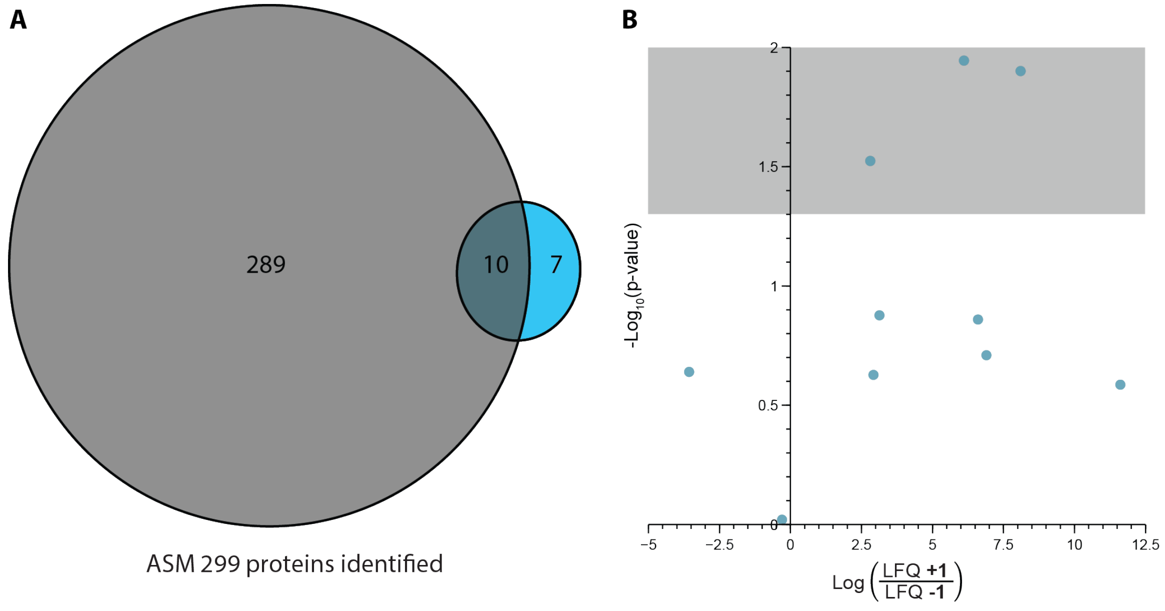

Figure S13 (related to Figure 7). (A) Venn diagram representing the number of proteins identified by mass spectrometry from the SORT-E elution (grey) and the no amino acid control (blue) of proteins from *D. melanogaster* ovaries. The majority of proteins identified in the no amino acid control were also identified in the corresponding SORT-E sample.

(B) Volcano plot of those 10 proteins identified in both the SORT-E sample (+**1**) and the no amino acid control sample (**-1**). The volcano plot shows the ratio of the LFQ values for each protein in this subset (i.e., the enrichment factor), plotted against the p-value of the null hypothesis that there is no difference between the LFQ values. The area shaded in gray corresponds to the threshold of p-values <0.05, i.e. values with a greater than 95% probability of correctly rejecting the null hypothesis.

Table S1 (related to Figure 4). Protein identifications and LFQ from MS analysis of SORT-E AGA(Ser), GCU(Ser), CAU(Met) and UUU(Lys). LFQ values for each replicate were generated in MaxQuant.

Table S2 (related to Figure 6). Protein identifications and relative quantitation from 9-plex TMT analysis of SORT-E AGA(Ser) and CAU(Met). Reporter ions 126, 127N and 127C correspond to E. coli control replicates, ions 128N, 128C and 129N correspond to AGA(Ser) or CAU(Met) input replicates (Labeled NPD1, 2 or 3) and ions 129C, 130N and 130C correspond to SORT-E AGA(Ser) or SORT-E CAU(Met) replicates (Labeled PD1, 2 or 3).

Table S3 (related to Figure 7). Protein identifications and LFQ from MS analysis of SORT-E from *D. melanogaster* ASM. LFQ values for each replicate were generated in MaxQuant.

II. Supplemental Methods

Chemical syntheses - general methods

All chemicals and solvents were purchased from Sigma-Alrich, Alfa Aesar or Fisher Scientific and used without further purification unless otherwise stated. Qualitative analysis by thin layer chromatography (TLC) was performed on aluminium sheets coated with silica (Merck TLC 60F-254). The spots were visualized under short wavelength ultra-violet lamp (254nm) or stained with basic, aqueous potassium permanganate, ethanolic ninhydrin or vanillin. Flash column chromatography was performed with specified solvent systems on silica gel 60 (mesh 230-400).

LC-MS analysis was performed on Agilent 1200 machine. The solvents used consisted of 0.2 % formic acid in water (buffer A) and 0.2 % formic acid in acetonitrile (buffer B). LC was performed using Phenomenex Jupiter C18 column (150 × 2 mm, 5μm) and monitored using variable wavelengths. Retention times (R_t) are recorded to a nearest 0.1 min and m/z ratio to nearest 0.01 mass units. The following programme was used for small molecule LC gradient: 0-1 min (A:B 10:90-10:90, 0.3 mL/min), 1-8 min (A:B 10:90-90:10, 0.3 mL/min), 8-10 min (A:B 90:10-90:10, 0.3 mL/min), 10-12 (A:B 90:10-10:90, 0.3 mL/min).

Mass spectrometry analysis following LC was carried out in ESI mode on a 6130 Quadrupole spectrometer and recorded in both positive and negative ion modes. NMR analysis was carried out on a Bruker 400MHz instrument. All reported chemical shifts (δ) relative to TMS were referenced to the residual protons in deuterated solvents used: d_1– chloroform (^{1}H δ = 7.26 ppm, ^{13}C δ = 77.16 ppm), d_6– dimethylsulfoxide (^{1}H δ = 2.49 ppm, ^{13}C δ = 39.52 ppm), D_2O (^{1}H δ = 4.70). APT or two-diomensional experiments (COSY, HSQC) were always performed to provide additional information used for analysis where needed. Coupling constants are given in Hz and described as: singlet – s, doublet – d, triplet – t, quartet – q, broad singlet – br, multiplet – m, doublet of doublets – dd, etc. and combinations thereof.

Synthesis of tetrazine diazobenzene biotin 2

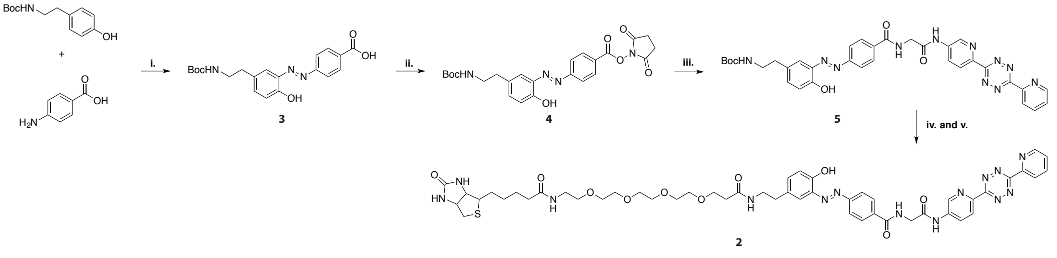

Scheme S1. Synthesis of **2**. *Reagents and conditions*: **i.** (a) 5N HCl, $NaNO_2$, 0 °C, (b) $NaHCO_3$, acetone, 0 °C, 71% yield; **ii.** *N*-hydroxysuccinimide, DMF, EDCI, DMAP, RT, 48% yield; **iii.** 2-amino-*N*-(6-(6-(pyridin-2-yl)-1,2,4,5-tetrazin-3-yl)pyridin-3-yl)acetamide, Et_3N, DMF, RT; **iv.** TFA, CH_2Cl_2, RT, quantitative yield; **v.** NHS-PEG$_4$-Biotin (Thermo scientific), DMF, Et_3N, RT, 18% yield.

i. (*E*)-4-((5-(2-((*tert*-butoxycarbonyl)amino)ethyl)-2-hydroxyphenyl)diazenyl)benzoic acid 3

$NaNO_2$ (629 mg, 9.12 mmol, 2.5 eq) was added to a suspension of 4-aminobenzoate (500 mg, 3.65 mmol, 1 eq) in hydrochloric acid (5N, 10 mL) at 0 ºC, in portions to maintain a constant 0 ºC. The suspension was stirred vigorously and after approximately 1 h, at 0 ºC, the solid had dissolved to give a yellow solution. The yellow solution was transferred to a dropping funnel and added dropwise to a suspension of *N*-boc-tyramine (865 mg, 3.65 mmol, 1 eq) and $NaHCO_3$ (4 g, 47.6 mmol, 13 eq) in saturated $NaHCO_3$ solution (40 mL) and acetone (5 mL) maintained at 0 ºC. The mixture was stirred vigorously for 18 h at 0 ºC and adjudged complete at this time. The reaction mixture was then diluted with HCl (1N, 100 mL) and the resultant precipitate filtered. The solid was washed with water (3× 30 mL) and then acetone (3× 30 mL) and dried under vacuum desiccation. **3** was obtained as a brown powder (1g, 71% yield). LRMS *m/z* (ES$^+$) 386 $[M+H]^+$, ^{1}H NMR analysis was in excellent agreement with the published values.

ii. 2,5-Dioxopyrrolidin-1-yl (*E*)-4-((5-(2-((*tert*-butoxycarbonyl)amino)ethyl)-2-hydroxyphenyl)diazenyl)benzoate 4

Carboxylic acid **3** (50 mg, 0.13 mmol, 1eq) was dissolved in DMF (1 mL), to this solution was added *N*-hydroxysuccinimide (18.1 mg, 0.16 mmol, 1.2 eq), followed by EDCI (30 mg, 0.16 mmol, 1.2 eq) and DMAP (8 mg, 0.07 mmol, 0.5 eq). The reaction mixture was stirred at room temeperature for 2 h and adjudged complete at this time by LCMS analysis. The reaction mixture was diluted with EtOAc (10 mL) and washed with HCl (1 M, 2× 10 mL) and then brine (10 mL). The organics were dried (Na_2SO_4) filtered and concentrated under vacuum to give a crude gum. The crude material was purified by silica gel column chromatography eluting EtOAc/hexane (40/60). The product fractions were combined and concentrated under vacuum to give **4** as a dark orange gum (30 mg, 48% yield). LRMS *m/z* (ES^-) 481 $[M-H]^-$

iii. *tert*-Butyl (*E*)-(4-hydroxy-3-((4-((2-oxo-2-((6-(6-(pyridin-2-yl)-1,2,4,5-tetrazin-3-yl)pyridin-3-yl)amino)ethyl)carbamoyl)phenyl)diazenyl)phenethyl)carbamate

Activated ester **4** (30 mg, 0.062 mmol, 1 eq) was dissolved in DMF (1 mL), to this solution was added aminotetrazine (32 mg, 0.093 mmol, 1.5 eq) followed by Et_3N (45 μL, 0.31 mmol, 5 eq). The reaction was stirred at room temperature for 1 h and was adjudged complete by LCMS analysis. The crude reaction mixture was diluted with EtOAc (5 mL) causing a precipitate to form. The solid was filtered and washed with HCl (1M, 5 mL). The solid was retained, and the filtrate extracted with more EtOAc (2× 5 mL). The organic fraction was dried under vacuum and the resultant solid combined with the solid isolated from filtration. This gave the desired product **5** (41 mg) as a red/orange solid, that used without further purification. LRMS *m/z* (ES^+) 676 $[M+H]^+$, *m/z* (ES^-) 674 $[M-H]^-$.

iv. (*E*)-4-((5-(2-Aminoethyl)-2-hydroxyphenyl)diazenyl)-*N*-(2-oxo-2-((6-(6-(pyridin-2-yl)-1,2,4,5-tetrazin-3-yl)pyridin-3-yl)amino)ethyl)benzamide 5a

Solid **5** (41 mg, 0.062 mmol, 1 eq) was suspended in CH_2Cl_2 (2 mL), to which was added TFA (2 mL) at room temperature, this immediately formed a red/orange solution. LCMS analysis after 5 minutes showed complete boc deprotection. The reaction was therefore concentrated to dryness by passing a stream of nitrogen over the reaction, giving the crude amine as a red/orange gum that was used directly without further purification. LRMS *m/z* (ES^+) 576 $[M+H]^+$.

v. Tetrazine-diazobenzene-biotin 2

Crude **5a** (approximately 0.062 mmol, 1eq) was dissolved in DMF (1 mL) to this was added NHS-Peg$_4$-Biotin (55 mg, 0.093 mmol, 1.5 eq, Thermo scientific EZ-Link NHS-PEG4-Biotin – 21363) and Et_3N (45 µL, 0.31 mmol, 5 eq). The reaction was monitored by LCMS analysis and was adjudged complete after 30 minutes. The reaction mixture was concentrated to dryness and the product purified by semi-preparative HPLC (10%-90% MeCN in H_2O over 35 minute gradient at 4 mL min^{-1} using Phenomenex Luna, 5µ, C18, 100 Å column). The product fractions were combined and freeze dried to give **2** (12 mg, 18% yield) as a red/orange powder. LRMS *m/z* (ES$^+$) 1049 [M+H]$^+$.

Expression, purification and labeling of proteins site-specifically incorporating 1.

*Expression of T4lysozyme K83-**1***

Electro-competent *E. coli* DH10B cells were co-transformed with pBK-*Mb*PylRS and pBAD-*T4LysK83*TAG-His6 PylT (Nguyen et al., 2011). Transformed cells were recovered in S.O.B. (1 mL, supplemented with 0.2% glucose) for 1 h at 37 ºC and used to inoculate LB containing 50 μg/mL kanamycin and 50 μg/mL tetracycline (LB-KT). The cells were incubated with shaking overnight at 37 ºC, 250 r.p.m. 1 mL of overnight culture was used to inoculate 100 mL of LB-KT½, which was then incubated (37 ºC, 250 r.p.m). At O.D.$_{600}$ ~0.3, the culture was divided equally and supplemented with either **1** (1 mM) or H_2O (500 μL) and incubated further (37 ºC, 250 r.p.m). At O.D.$_{600}$ ~0.6 protein expression was induced by the addition of arabinose (0.2%). After 4 h, the cells were harvested by centrifugation (4000 r.p.m, 20 min) and the pellet frozen until further use.

*Expression of Ubiquitin K6-**1** and Ubiquitin K48-**1***

Chemically competent *E. coli* BL21(DE3) cells (Merck Biosciences) were co-transformed with pBK-*Mb*PylRS and pCDF-pylT-UbTAG6-His6 or pCDF-pylT-UbTAG48-His6 (Madrzak et al., 2015; Virdee et al., 2010). Transformed cells were recovered in S.O.B. (1 mL, supplemented with 0.2% glucose) for 1 h at 37 ºC and used to inoculate LB containing 50 μg/mL kanamycin and 25 μg/mL spectinomycin (LB-KS). The cells were incubated with shaking overnight at 37 ºC, 250 r.p.m. 1 mL of overnight culture was used to inoculate 100 mL of LB-KS½, which was then incubated (37 ºC, 250 r.p.m). At O.D.$_{600}$ ~0.3, the culture was divided equally and supplemented with either **1** (1 mM) or H_2O (500 μL) and incubated further (37 ºC, 250 r.p.m). At O.D.$_{600}$ ~0.6 protein expression was induced by the addition of IPTG (0.5 mM). After 4 h, the cells were harvested by centrifugation (4000 r.p.m, 20 min) and the pellet frozen until further use.

*Purification of T4lysozyme K83-**1**, Ubiquitin K6-**1** and Ubiquitin K48-**1** from E. coli*

The frozen bacterial pellets were thawed on ice and resuspended in 2.5 mL lysis buffer (Bugbuster®, Novagen®, 50 μg/mL DNAse 1, Roche inhibitor cocktail and 20 mM imidazole). Cells were incubated (4 ºC, 30 minutes) then clarified by centrifugation (16000 g, 4 ºC, 30 minutes). The clarified lysates were transferred to fresh tubes and 100 μL Ni-NTA slurry added. The mixtures was incubated with agitation (4 ºC, 1 h) and then collected by centrifugation (1000 g, 4 ºC, 5 min). The beads were resuspended three times in 500 μL wash buffer (10 mM Tris-HCL, 40 mM imidazole, 200 mM NaCl, pH 8) and collected by centrifugation (1000 g, 4 ºC, 5 min). Finally, the beads were resuspended in 100 μL elution buffer (10 mM Tris-HCL, 300 mM imidazole, 200 mM NaCl, pH 8), pelleted by centrifugation (1000 g, 4 ºC, 5 min) and the supernatant collected into fresh tubes. The elution was repeated three times with 100 μL of elution buffer. The purified proteins were analysed by 4-12% SDS-PAGE and LC-MS and a Bradford assay was performed to determine protein concentration (using BSA standard, for the standard curve).

Intact Protein Mass Spectrometry

ESI-MS was carried out using an Agilent 1200 LC-MS system with a 6130 Quadrupole spectrometer. The solvent system consisted of 0.2 % (v/v) formic acid in H_2O as buffer A, and 0.2 % (v/v) formic acid in acetonitrile (MeCN) as buffer B. Protein UV absorbance was monitored at 214 and 280 nm. Protein MS spectra were acquired in positive ionisation mode, scanning between 400-2000 m/z. Collected spectra were averaged over the entire total ion current (TIC). Intact protein masses were calculated via spectral deconvolution using Agilent's LC/MSD Chemstation software with built-in deconvolution tool. The default deconvolution parameters were used (masses between 500-50000 Da, with a maximum allowable charge of +50, a minimum of 5 peaks in a peak set, a noise cut off of 1000 counts and an abundance cut off at 10% for selected peaks).

*In vitro labeling of T4lysozyme K83-**1**, Ubiquitin K6-**1** and Ubiquitin K48-**1** with Probe **2** and subsequent reduction with $Na_2S_2O_4$*

Purified proteins were diluted to a concentration of 20 μM in 8M GdmCl and used at a final reaction volume of 50 μL. **2** (10 molar equivalents, 5 μL from a 2 mM stock solution in DMSO) was added and the reactants mixed by aspirating several times, the mixture was then incubated at room temperature. The reaction progress was monitored by LC-MS (injecting 5 μL/100 pmol of protein) and showed complete conversion to the ligated products T4lysozyme K83-**ii,** Ubiquitin K6-**ii** and Ubiquitin K48-**ii** (**Figure 2**) after 4 hours.

Test reduction with $Na_2S_2O_4$

The crude reaction mixtures were then treated with $Na_2S_2O_4$ (25 mM, 4 μL of a 250 mM stock), mixed by aspirating several times and incubated at room temperature. The reaction progress was monitored by LC-MS (injecting 5 μL/100 pmol of protein) and showed complete reduction to the desired products T4lysozyme K83-**iii,** Ubiquitin K6-**iii** and Ubiquitin K48-**iii** (**Figure 2**) within 30 minutes. Note: The reaction turned from yellow to colourless with seconds of adding the $Na_2S_2O_4$.

Proteomic incorporation of 1 via SORT in E. coli and chemoselective labeling of lysates with tetrazine probes

*Proteomic incorporation of **1** via SORT*

Electrocompetent *E. coli* DH10B cells (50 μL) were doubly transformed with pBAD_wtT4L_MbPylT$_{XXX}$ plasmid (Elliott et al., 2014) (2 μL, necessary for expression of PyltRNA$_{XXX}$) and pBK-*Mb*PylS plasmid (Elliott et al., 2014) (2 μL necessary for expression of PylRS). Transformed cells were recovered in 1 ml S.O.B. (supplemented with 0.2% glucose) for 1 h at 37 °C. 100 μL of the recovery was used to inoculate 50 ml LB-KT (50 μg/ml kanamycin and 25 μg/ml tetracycline) and the cultures incubated overnight (37 °C, 250 r.p.m.). Overnight cultures were diluted to OD_{600} 0.3 with LB-KT½ and divided into two 50 mL culture. One culture was supplemented with **1** (0.1 mM final concentration) and other with H_2O (50 μL). Cultures were then incubated (37 °C, 250 r.p.m.). At OD_{600} ~ 0.6 the cultures were incubated for a further 4 h. Cells were

harvested by centrifugation (4,000 r.p.m., 4 °C, 20 min) and then washed three times by resuspending in 1 mL of ice cold PBS, and collecting by centrifugation (4,000 r.p.m., 4 °C, 20 min) between each wash. The final bacterial pellets were immediately frozen for storage.

*SORT-M: Chemoselective labeling of proteomes tagged with **1** by SORT with tetrazine-fluorophore conjugates.*

Frozen bacterial pellets were resuspended in 5 ml Urea buffer (8M urea, 25 mM Tris-HCl, pH 8) and lysed using a microtip sonicator (amplitude 15, 90 s total sonication time, Soniprep 150 *plus*). The lysate was clarified by centrifugation (4 °C, 14,000 r.p.m., 30 min) and the supernatant transfered to a fresh tube. A Bradford assay was performed to determine protein concentration (using BSA standards for the standard curve). To 50 μL of cleared cell lysate was added tetrazine-fluorophore conjugate **6** (4 μM final concentration, from a 200 μM stock in DMSO, **Figure S3**) (Lang et al., 2012). The reactions were mixed by aspirating several times and the samples then incubated in the dark (room temperature, 4 h). After this time a 5 μL aliquot was diluted into 10 μL of pure water; 5 μL of 4× LDS sample buffer supplemented (6 mM BCN-OH and 20 mM DTT) was added and mixed by vortexing gently. Samples were incubated for 10 min before boiling at 90 °C for 10 min. Samples were analysed by 4–12% SDS-PAGE and fluorescent images were acquired using a Typhoon Trio phosphoimager (GE Life Sciences).

*SORT-E: Chemoselective labeling of proteomes tagged with **1** by tetrazine-diazobenzene-biotin conjugate **2**, enrichment and elution of SORT-E proteins*

Cleared bacterial protein lysates were prepared in the same way as described above. For capture, enrichment and elution of tagged proteins, 500 μL of cleared cell lysate (8 mg mL^{-1} protein lysate) was typically used. Thus, to 500 μL of cleared cell lysate DTT (1 mM, final concentration), was added and mixed with gentle vortexing, the mixture was incubated at room temperature for 45 minutes. After this time iodoacetamide (5.5 mM, final concentration) was added, mixed with gentle vortexing, then incubated for 30 minutes at room temperature. **2** (5 μL, 20 μM final concentration, from a 2 mM stock in

DMSO) was then added and the reactions were mixed by gentle vortexing and the samples then incubated in the dark at room temperature overnight with end-over-end rotation (Hula mixer). 10 μL aliquots were taken at this stage as input sample for subsequent SDS-PAGE analysis. The samples were then diluted with PBS to a final volume of 5 mL and high capacity streptavidin beads (150 μL of settled resin, pre-equilibrated in PBS, Thermo scientific Streptavidin Agarose resin) added. The beads were incubated with end-over-end rotation (Hula mixer) for 1.5 hours, and then collected by gravity filtration through a column (Poly-Prep® chromatography column Bio-Rad 731-1550). The beads were resuspended in urea buffer (500 μL, 8M urea, 25 mM tris, pH 8) and transferred to a smaller spin column (Mini Bio-spin® chromatography column Bio-Rad 731-1550). The beads were collected by mild centrifugation (1000 rpm, 5 second pulse) and washed 2× urea buffer (500 μL) then 2× 1% SDS in PBS (500 μL) with 15 minute incubation times between each wash. The final wash acts as a control for the $Na_2S_2O_4$ specific elution, the beads were resuspended in 1% SDS in PBS (150 μL) and incubated at room temperature for 30 minutes with end-over-end rotation (Hula mixer). The beads collected by mild centrifugation (1000 rpm, 5 second pulse) and the supernatant kept for subsequent analysis by SDS-PAGE. Specifically bound proteins were then eluted by resuspending the beads in 1% SDS in PBS supplemented with 25 mM $Na_2S_2O_4$ (150 μL) and incubating at room temperature for 30 minutes with end-over-end rotation (Hula mixer). The supernatant was collected by mild centrifugation (1000 rpm, 5 second pulse) and analysed by SDS-PAGE.

SDS-PAGE analysis and LC-MS/MS of enriched proteins tagged via SORT

5 μL aliquots of each lysate input sample was diluted into 10 μL of pure water; 15 μL of each final wash sample and 15 μL of each elution sample were transferred to fresh tubes. 5 μL of 4× LDS sample buffer (supplemented with 80 mM DTT) was added and mixed by vortexing gently. Samples were incubated for 10 min before boiling at 90 °C for 10 min. Samples were analyzed by 4–12% SDS-PAGE, 200 V constant, 40 minutes. Gels were stained with Sypro®-Ruby using the standard manufacturer's protocol and imaged with a Typhoon Trio phosphoimager (GE Life Sciences) excitation – 410 nm, emission filter at 580 nm.

For LC-MS/MS each pull-down was performed on 3 biological replicates. Eluted proteins were loaded onto 4–12% SDS-PAGE precast gels, as described above and each lane excised and cut into approximately 24 slices. The excised protein gel pieces were placed in a well of a 96-well microtiter plate and destained with 50% v/v acetonitrile and 50 mM ammonium bicarbonate, reduced with 10 mM DTT and alkylated with 55 mM iodoacetamide. After alkylation, proteins were digested with 6 ng/μL trypsin (Promega, UK) overnight at 37 °C. The resulting peptides were extracted in 2% v/v formic acid, 2% v/v acetonitrile. The digest was analyzed by nano-scale capillary LC-MS/MS using an Ultimate U3000 HPLC (Thermo Scientific Dionex, San Jose, USA) to deliver a flow of ~300 nL/min. A C18 Acclaim PepMap100 5 μm, 100 μm × 20 mm nanoViper (Thermo Scientific Dionex, San Jose, USA) trapped the peptides before separation on a C18 Acclaim PepMap100 3 μm, 75 μm × 250 mm nanoViper (ThermoScientific Dionex, San Jose, USA). Peptides were eluted with a gradient of acetonitrile. The analytical column outlet was directly interfaced by means of a modified nano-flow electrospray ionization source, with a hybrid dual-pressure linear ion trap mass spectrometer (Orbitrap Velos, Thermo Scientific, San Jose, USA). Data-dependent analysis was carried out, using a resolution of 30,000 for the full MS spectrum, followed by ten MS/MS spectra in the linear ion trap. MS spectra were collected over a *m*/*z* range of 300–2,000. MS/MS scans were collected using a threshold energy of 35 for collision-induced dissociation. LC-MS/MS data were then searched against a protein database (UniProt KB) using the Mascot search engine program (Matrix Science, UK). Database search parameters were set with a precursor tolerance of 5 p.p.m. and a fragment ion mass tolerance of 0.8 Da. Two missed enzyme cleavages were allowed and variable modifications for oxidized methionine, carbamidomethyl cysteine, pyroglutamic acid, phosphorylated serine, threonine and tyrosine were included. Proteomic data were worked up in the MaxLFQ software suite (1) to identify proteins from peptide sequences and calculate label-free quantifications (LFQ).

SORT-E: Combined with a 9-Plex TMT analysis

Production of control protein lysates

E. coli DH10B cells were inoculated into three LB cultures (50mL) and incubated overnight (37 °C, 250 r.p.m.). Overnight cultures were diluted to OD_{600} 0.3 with LB (250mL) and incubated (37 °C, 250 r.p.m.). At OD_{600} ~ 0.6 the cultures were incubated for a further 4 h. Cells were then harvested by centrifugation (4,000 r.p.m., 4 °C, 20 min) and frozen until further use.
Frozen bacterial pellets were resuspended in 50 mL Urea buffer (8M urea, 25 mM tris, pH 8) and lysed using a microtip sonicator (amplitude 15, 90 s total sonication time, Soniprep 150 *plus*). The lysate was cleared by centrifugation (4 °C, 14,000 r.p.m., 30 min) and the supernatant aspirated to a fresh tube, a Bradford assay was performed to determine protein concentration (using BSA standards, for standard curve).

Production of SORT-E input material
For SORT-E AGA(Ser) and SORT-E CAU(Met) three biological replicates of cleared bacterial protein lysates were prepared in the same way as described above for SORT-E, but scaled up by a factor of 10.

Production of E. coli SORT-E material
For SORT-E AGA(Ser), 3.5 mL (25 mg protein) of the above cleared protein lysate were used for each replicate. For SORT-E CAU(Met), 7 mL (50 mg protein) of the above cleared protein lysates were used for each replicate. Thus, to each lysate DTT (1 mM) was added and mixed with gentle vortexing, the mixture was incubated at room temperature for 45 minutes. After this time iodoacetamide (5.5 mM) was added, mixed with gentle vortexing, then incubated for 30 minutes at room temperature. **2** (5 µL, 20 µM final concentration, from a 2 mM stock in DMSO) was then added and the reactions were mixed by gentle vortexing and the samples then incubated in the dark at room temperature overnight with end-over-end rotation (Hula mixer). 10 µL aliquots were taken at this stage as input sample for subsequent SDS-PAGE analysis. The labelled protein samples were precipitated by the addition of 10 volumes of ice-cold methanol and incubated at -25 °C overnight. The proteins were pelleted by centrifugation (1000g, 30min) and washed three times with further volumes (10 mL) of ice-cold methanol. Washed protein pellets were re-suspended in 3.5 mL or 7 mL of Urea buffer (8M urea, 25

mM tris, pH 8) respectively. High capacity streptavidin beads (50 µL of settled resin, Thermo scientific Streptavidin Agarose resin) were added to each sample and incubated with end-over-end rotation (Hula mixer) for 1.5 hours. Beads were then collected by gravity filtration through a column (Poly-Prep® chromatography column Bio-Rad 731-1550). The beads were resuspended in urea buffer (500 µL, 8M urea, 25 mM tris, pH 8) and transferred to a smaller spin column (Mini Bio-spin® chromatography column Bio-Rad 731-1550). The beads were collected by mild centrifugation (1000 rpm, 5 second pulse) and washed 2× urea buffer (500 µL) then 2× 1% SDS in PBS (500 µL) with 15 minute incubation times between each wash. The final wash acts as a control for the $Na_2S_2O_4$ specific elution, the beads were resuspended in 1% SDS in PBS (50 µL) and incubated at room temperature for 30 minutes with end-over-end rotation (Hula mixer). The beads were collected by mild centrifugation (1000 rpm, 5 second pulse) and the supernatant kept for subsequent analysis by SDS-PAGE. Specifically bound proteins were then eluted by resuspending the beads in 1% SDS in PBS supplemented with 50 mM $Na_2S_2O_4$ (50 µL) and incubating at room temperature for 30 minutes with end-over-end rotation (Hula mixer). The supernatant was collected by mild centrifugation (1000 rpm, 5 second pulse), this elution step was repeated a further four times and the eluents combined to give a total of 250 µL SORT-E protein fraction for each sample. A 15 µL aliquot was taken for SDS-PAGE analysis. SORT-E protein eluents were then 10-fold diluted with PBS and then concentrated using centrifugal spin filters (3 kDa cut-off, Merk-Millpore Amicon Ultra-0.5 mL) to a final volume of approximately 100 µL. Protein concentration was determined by a BCA colorimetric assay (ThermoFisher – 23225).

Preparation of 9-Plex TMT analysis

50 µg of each protein sample replicate for each test condition (3× control proteins, 3× SORT-E input and 3× SORT-E) were trypsinised and the resulting peptides labelled with unique isobaric mass tags as per the manufacturers instructions (TMT10plex™ Isobaric Mass Tag Labeling Kit; ThermoFisher – 90113). An aliquot of the TMT labelled pool was evaporated to dryness, resuspended in 5% formic acid and then desalted using SepPak cartridges according to the manufacturers instructions (Waters, Milford,

Massachusetts, USA). Eluate from the SepPak cartridge was again evaporated to dryness and resuspended in 1% formic acid prior to analysis by nano-LC MSMS using an Orbitrap Fusion Tribrid Mass Spectrometer.

Nano-LC Mass Spectrometry

The sample was fractionated using an Ultimate 3000 nanoHPLC system in line with an Orbitrap Fusion Tribrid mass spectrometer (Thermo Scientific). In brief, peptides in 1% (vol/vol) formic acid were injected onto an Acclaim PepMap C18 nano-trap column (Thermo Scientific). After washing with 0.5% (vol/vol) acetonitrile 0.1% (vol/vol) formic acid peptides were resolved on a 250 mm × 75 μm Acclaim PepMap C18 reverse phase analytical column (Thermo Scientific) over a 150 min organic gradient, using 7 gradient segments (1-6% solvent B over 1min., 6-15% B over 58min., 15-32%B over 58min., 32-40%B over 5min., 40-90%B over 1min., held at 90%B for 6min and then reduced to 1%B over 1min.) with a flow rate of 300 nl min^{-1}. Solvent A was 0.1% formic acid and Solvent B was aqueous 80% acetonitrile in 0.1% formic acid. Peptides were ionized by nano-electrospray ionization at 2.0kV using a stainless steel emitter with an internal diameter of 30 μm (Thermo Scientific) and a capillary temperature of 275°C.

All spectra were acquired using an Orbitrap Fusion Tribrid mass spectrometer controlled by Xcalibur 2.0 software (Thermo Scientific) and operated in data-dependent acquisition mode using an SPS-MS3 workflow. FTMS1 spectra were collected at a resolution of 120 000, with an automatic gain control (AGC) target of 200 000 and a max injection time of 50ms. The TopN most intense ions were selected for MS/MS. Precursors were filtered according to charge state (to include charge states 2-7) and with monoisotopic precursor selection. Previously interrogated precursors were excluded using a dynamic window (40s +/-10ppm). The MS2 precursors were isolated with a quadrupole mass filter set to a width of 1.2m/z. ITMS2 spectra were collected with an AGC target of 5000, max injection time of 120ms and CID collision energy of 35%.

For FTMS3 analysis, the Orbitrap was operated at 60 000 resolution with an AGC target of 50 000 and a max injection time of 120ms. Precursors were fragmented by high energy collision dissociation (HCD) at a normalised collision energy of 55% to ensure

maximal TMT reporter ion yield. Synchronous Precursor Selection (SPS) was enabled to include up to 5 MS2 fragment ions in the FTMS3 scan.

Analysis of Proteomic Data

Shotgun proteomic data were worked up in the MaxLFQ software suite (1) to identify proteins from peptide sequences and calculate label-free quantifications (LFQ). Identified proteins and LFQs were exported to Excel, where proteins were counted and compared across different experiments. The number of proteins in common between SORT-E (AGA, Ser), SORT-E (GCU, Ser), SORT-E (CAU, Met) and SORT-E (UUU, Lys) were calculated in Excel, and Venn-Diagrams were constructed using eulerAPE (2). For further analyses, identified proteins were paired with their molecular weights (MW), relative abundances, and codon counts (the number of occurrences of a given codon within the protein's open reading frame). Codon counts and molecular weights were extracted on a protein-by-protein basis by parsing through the annotated genebank file for the *E. coli* str. K-12 substr. MG1655 whole genome (accession code NC_000913), matching gene names (including synonyms) against our proteomic data set, and extracting (or calculating) the relevant data. These operations were automated through a python script called `CodonCounter.py` (provided as supplemental program 1) equipped with the BioPython package. Protein abundances were taken from an integrated meta-dataset of *E. coli* protein abundances (accession code 511145), compiled by PaxDB (http://pax-db.org/). As the abundances were indexed under locus tags, `CodonCounter.py` also cross-referenced locus to gene names in order to assist the compilation of these data.

Sorting and t-test calculations were carried out in Excel, and plots were prepared in Veusz (http://home.gna.org/veusz/).

Analysis of Tandem Mass Tag (TMT) Proteomic Data

Protein mixtures obtained from one of three replicates for one of five test conditions (unmodified *E. coli* lysate, lysates from *E. coli* equipped with components to perform SORT-E (AGA, Ser) or SORT-E (CAU, Met) (i.e., the SORT-E inputs), and the

corresponding SORT-E enrichments were reacted with one of nine tandem mass tag labels, and submitted to LC-MS/MS in sets of nine.

The raw data files were processed and quantified using Proteome Discoverer software v1.4 (Thermo Scientific). Peptide precursor mass tolerance was set at 10ppm, and MS/MS tolerance was set at 0.6Da. Search criteria included oxidation of methionine (+15.9949 Da) as a variable modification and carbamidomethylation of cysteine (+57.0214 Da) and the addition of the TMT mass tag (+229.163 Da) to peptide N-termini and lysine as fixed modifications. Searches were performed with full tryptic digestion and a maximum of 1 missed cleavage was allowed. The reverse database search option was enabled and all peptide data was filtered to satisfy a false discovery rate (FDR) of 5%. Proteome Discoverer searched peptide sequences against the UniProt *E. coli* database using the SEQUEST algorithm to assign peptides to proteins. Relative peptide abundances were calculated based on TMT ion counts. The protein abundance was taken as the median abundance across all peptides identified to that protein, and these data were exported to an Excel spreadsheet. Statistics for protein abundances were generated by calculating the mean, standard deviation, and coefficient of variation (see **Figure S7 D-H**) of these medians across the three biological replicates.

The sum of the TMT ion counts across all proteins identified in the SORT-E pull-down experiments was about one-fourth the analogous sums for the non-pull-down experiments, despite the fact that an identical amount of protein was loaded for all experiments. We believe this is because SORT-E elution conditions solubilize a streptavidin contaminant from beads (which appears in gels as a dense low molecular-weight band in elution lanes (e.g., elution lanes in Figures 3, S5)), which lowers the fraction of peptides arising from *E. coli* proteins. Before further analysis, we therefore normalized the TMT ion counts from every mass channel, making the sum of all TMT ion counts in each channel the same.

Where we refer to a protein's SORT-E pull down efficiency, we mean the average of that protein's TMT ion counts from the three mass channels derived from the enrich samples divided by the average of that protein's TMT ion counts from the three mass channels derived from the input samples prior to enrichment.

Mathematical manipulations, sorting, and t-test calculations were carried out in Excel, and plots were prepared in Veusz.

Fly lines and culture conditions

All flies were grown at 25°C on standard Iberian medium. Flies were fed **1** by mixing dried yeast with a solution of **1** (10mM) to form a paste. This paste was added as a supplement to the normal Iberian fly food for a minimum of 24h and the yeast was changed daily.

Double and triple sense codon lines were created by recombination using the original lines FT58 (A = $PylT_{UGC}$, Ala), FT60 (S = $PylT_{GCU}$, Ser), FT62 (L = $PylT_{CAG}$, Leu) and FT63 (M = $PylT_{CAU}$, Met)(Elliott et al., 2014). Trans-heterozygous virgins were collected for each pair-wise combination of sense codon (AS – FT58/FT60, AL – FT58/FT62, AM – FT58/FT63, SL – FT60/FT62, SM – FT60/FT63, and LM – FT62/FT63) and crossed to males of the third chromosome balancer stock *w*; ; TM3/TM6. Potential recombinant males were identified based on eye colour, and individuals were backcrossed to virgins of *w*; ; TM3/TM6 to make a balanced stock. Recombinant lines were then screened by crossing to nos-vp16-GAL4 virgins (Bloomington 4937) to create FT58-60/nos-vp16-GAL4 (AS), FT58-62/nos-vp16-GAL4 (AL) etc. and compared with the original single sense codon lines FT58/nos-vp16-GAL4, FT60/nos-vp16-GAL4, FT62/nos-vp16-GAL4 and FT63/nos-vp16-GAL4. The females were fed 10 mM **1** for 24 - 48h and then the ovaries were extracted from 15 females of the indicated genotype and labeled with 4 μM **7** (**Figure S11**) for 2h as described (Elliott et al., 2014).

Triple sense codon lines were generated in a similar manner. In this case the double sense codon lines were crossed to a different single sense codon line to generate trans-heterozygotes with three different sense codons. In this case not all combinations produced viable trans-heterozygotes and in some cases trans-heterozygotes were viable but gave no potential recombinant males. Successful combinations were FT58-63 (AM)/FT60 (S), FT60-63 (SM)/FT62 (L) and FT58-60 (AS)/FT63 (M) which generated the lines FT58-60-63 (ASM) and FT60-62-63 (SLM). These lines were screened by

crossing to nos-vp16-GAL4 virgins and compared with the double sense codon lines as described above (Elliott et al., 2014).

SORT-E from D. melanogaster

Ovaries were dissected from 250 females flies of FT58-60-63/nos-vp16-Gal4 (ASM) which had been fed normal food either supplemented with **1** (10 mM) or without **1**. The ovaries were homogenized into 8 M urea 15 mM tris (250 μL) and the resultant protein lysate clarified by filtration. Bradford assay was used to determine the protein concentration. For SORT-E and subsequent mass spectrometry, typically 7 mg of fly ovary protein was used and labeled with **2** (20 μM) in an identical procedure as described in the protocol for *E. coli* above.

III. Supplemental program

Python scripts

```
#CodonCounter.py
#Call with one argument, a file with a list of proteins
#Requires Biopython package, an annotated genbank file, and the PaxDB
file if abundance data is desired


from Bio import SeqIO
import sys
from Bio.Seq import Seq
from Bio.Alphabet import IUPAC
from Bio import SeqUtils
import numpy as np

proteinList = open( sys.argv[1] , 'r')
proteinListOut_fn = sys.argv[1].split('.')[0] + '_out.txt'
proteinListOut = open( proteinListOut_fn , 'w')

#Will hold tuples of form (TCT,ATG,AGC,AGT,AAA,AAG)
codons = []

K12 = SeqIO.read("Ecoli K12 genome.gb", "genbank")
abundanceData = np.genfromtxt('511145-E.coli_whole_organism-
integrated_dataset.txt',skiprows=9, dtype=None)
abundanceRange = range( abundanceData.shape[0] )
abundanceLocusTags = []
abundances = []
for i in abundanceRange:
    abundanceLocusTags.append(  abundanceData[i][1].split('.')[1] )
    abundances.append( abundanceData[i][2] )


for entry in proteinList:
    found = 0
    protein = entry.split()[0]
    for feature in K12.features:
        if (feature.type=="CDS"):
            gene = feature.qualifiers['gene'][0]
            synonyms = [x.strip() for x in
feature.qualifiers['gene_synonym'][0].split(';')]
            synonyms.append(gene)
            if protein in synonyms:
                found = 1
                start = feature.location.start.position
                end = feature.location.end.position
                sense = feature.strand
                locustag = feature.qualifiers['locus_tag'][0]
                if sense == 1:
                    geneseq = K12.seq[start:end]
                else:
                    geneseq = K12.seq[start:end].reverse_complement()
                if locustag in abundanceLocusTags:
```

```
                    abundanceIndex = abundanceLocusTags.index( locustag
)
                    abundance = abundances[ abundanceIndex ]
                else:
                    abudance = 0
                codons = [geneseq[i:i+3] for i in
range(0,len(geneseq),3)]
                TCTs = codons.count( 'TCT' )
                TCCs = codons.count( 'TCC' )
                TCAs = codons.count( 'TCA' )
                ATGs = codons.count( 'ATG' )
                proteinListOut.write('%s \t %s \t %s \t %s \t %s \t %s
\n' %(protein,TCTs,TCCs,TCAs,ATGs,abundance))
                break
    if found == 0:
        proteinListOut.write('**' + entry)

proteinList.close()
proteinListOut.close()
```

SASA calculator

```
#GetSASAscores.py
#This script is fed a list with this structure
#     gene-name,  PDB-id,     chain-id
#     gene-name,  PDB-id,     chain-id
#     ...
#For each protein, it calculates the SASA score for each site, the tRNA
decoding efficiency for each site
#and provides final scores

#This function writes up a SASA-calculating program that is shelled out
to pymol as a subprocess
#It needs to know the PDB-id and chain-id of the protein you want
def writeProgram( PDB, chain, dPos ):
    programText = """
import pymol
from pymol import cmd
from pymol import stored
pymol.finish_launching()

cmd.fetch('%s')
stored.resids = %s
stored.resnames = []

for i in stored.resids:
    #check if residue exists
    stored.test = []
    cmd.iterate('resi '+str(i)+' and chain %s and name CA',
'stored.test.append(resn)')
    if stored.test == []:
        stored.resnames.append('NONE')
    else:
        cmd.iterate('resi '+str(i)+' and chain %s and name CA',
'stored.resnames.append(resn)')
    stored.test = []
```

```
cmd.set('dot_solvent', 1)
cmd.set('dot_density', 2)

stored.sasa_per_residue = []
for i in stored.resids:
    #check if residue exists
    stored.test = []
    cmd.iterate('resi '+str(i)+' and chain %s and name CA',
'stored.test.append(resn)')
    if stored.test == []:
        stored.sasa_per_residue.append(0.8)
    else:
        stored.sasa_per_residue.append(cmd.get_area('resi '+str(i)+'
and chain %s'))

stored.sa_per_residue = []
for i in stored.resnames:
    if i == 'ALA':
        stored.sa_per_residue.append(106)
    elif i == 'CYS':
        stored.sa_per_residue.append(135)
    elif i == 'ASP':
        stored.sa_per_residue.append(163)
    elif i == 'GLU':
        stored.sa_per_residue.append(194)
    elif i == 'PHE':
        stored.sa_per_residue.append(197)
    elif i == 'GLY':
        stored.sa_per_residue.append(84)
    elif i == 'HIS':
        stored.sa_per_residue.append(184)
    elif i == 'ILE':
        stored.sa_per_residue.append(169)
    elif i == 'LYS':
        stored.sa_per_residue.append(205)
    elif i == 'LEU':
        stored.sa_per_residue.append(164)
    elif i == 'MET':
        stored.sa_per_residue.append(188)
    elif i == 'ASN':
        stored.sa_per_residue.append(157)
    elif i == 'PRO':
        stored.sa_per_residue.append(136)
    elif i == 'GLN':
        stored.sa_per_residue.append(198)
    elif i == 'ARG':
        stored.sa_per_residue.append(248)
    elif i == 'SER':
        stored.sa_per_residue.append(130)
    elif i == 'THR':
        stored.sa_per_residue.append(142)
    elif i == 'VAL':
        stored.sa_per_residue.append(142)
    elif i == 'TRP':
        stored.sa_per_residue.append(227)
    elif i == 'TYR':
        stored.sa_per_residue.append(222)
```

```
    elif i == 'NONE':
        stored.sa_per_residue.append(1)
    else:
        stored.sa_per_residue.append(180)

stored.sasa_relative = [x/y for x,y in zip(stored.sasa_per_residue,
stored.sa_per_residue)]
print stored.sasa_relative
    """ %(PDB,dPos,chain,chain, chain, chain)
    return programText

import sys
import os
import re
import subprocess
from Bio import SeqIO
from Bio.Seq import Seq
from Bio.Alphabet import IUPAC
from Bio import SeqUtils
import numpy as np


#call the program with a csv, as above
listFn = sys.argv[1]
list = open( listFn , 'r')

outListFn = listFn.split('.')[0]+'_out.txt'
outList = open( outListFn , 'w')

sasaListFn = listFn.split('.')[0]+'_sasa.txt'
sasaList = open( sasaListFn , 'w')

K12 = SeqIO.read("Ecoli K12 genome.gb", "genbank")

for entry in list:
    geneName = entry.split()[0]
    try:
        PDBid = entry.split()[1]
    except IndexError:
        PDBid = '.'
    try:
        chainid = entry.split()[2]
    except IndexError:
        pass
    for feature in K12.features:
        if (feature.type=="CDS"):
            if (feature.qualifiers['gene'][0] == geneName):
                start = feature.location.start.position
                end = feature.location.end.position
                sense = feature.strand
                locustag = feature.qualifiers['locus_tag'][0]
                if sense == 1:
                    geneseq = K12.seq[start:end]
                else:
                    geneseq = K12.seq[start:end].reverse_complement()
                codons = [geneseq[i:i+3] for i in
range(0,len(geneseq),3)]
                Mets = []
```

```
                Sers = []
                for j in range(1, len(codons)): #skip the initial codon
because that uses initiator tRNA
                    if codons[j] == 'ATG':
                        Mets.append( j+1 )
                    if codons[j] == 'TCT' or codons[j] == 'TCA' or
codons[j] == 'TCC':
                        Sers.append( j+1 )
                decodablePositions = str( Mets + Sers)
                break
    #Calculate SASA at all the decodable positions in a subprocess that
uses the pymol interpreter

    if PDBid == '.':
        outList.write(geneName + '\n')
    else:
        SASAfn = geneName+'.py'
        SASAscript = open( SASAfn , 'w' )
        SASAscript.write( writeProgram( PDBid, chainid,
decodablePositions ) )
        SASAscript.close()
        SASAoutput = subprocess.check_output('pymol -rqc %s'%SASAfn,
shell=True)

        #Format the resulting output
        begin = SASAoutput.find('[')
        end = SASAoutput.find(']')
        SASAoutput1 = SASAoutput[begin:end]
        SASAs = [ round(float( re.sub("[^0-9.]", "", x) ) , 2) for x in
SASAoutput1.split(',') ]
            #makes sure all non-numerical characters are removed before
trying to cast as float

        #Determine the Met Score
        if len(Mets) == 0:
            MetMedian = 0
            MetMax = 0
        else:
            MetMedian = np.median(SASAs[0:len(Mets)])
            MetMax = np.max(SASAs[0:len(Mets)])

        #Determine the Ser Score
        if len(Sers) == 0:
            SerMedian = 0
            SerMax = 0
        else:
            SerMedian = np.median(SASAs[len(Mets):len(Mets)+len(Sers)])
            SerMax = np.max(SASAs[len(Mets):len(Mets)+len(Sers)])

        outList.write(geneName + '\t %s \t %s \t %s \t %s
\n'%(MetMedian,MetMax,SerMedian,SerMax))
        sasaList.write(geneName + '\n' + str(SASAs) + '\n')
list.close()
outList.close()
sasaList.close()
```

IV. References

Dong, H., Nilsson, L., and Kurland, C.G. (1996). Co-variation of tRNA abundance and codon usage in Escherichia coli at different growth rates. Journal of molecular biology *260*, 649-663.

Elliott, T.S., Townsley, F.M., Bianco, A., Ernst, R.J., Sachdeva, A., Elsasser, S.J., Davis, L., Lang, K., Pisa, R., Greiss, S.*, et al.* (2014). Proteome labeling and protein identification in specific tissues and at specific developmental stages in an animal. Nature biotechnology *32*, 465-472.

Lang, K., Davis, L., Torres-Kolbus, J., Chou, C., Deiters, A., and Chin, J.W. (2012). Genetically encoded norbornene directs site-specific cellular protein labeling via a rapid bioorthogonal reaction. Nature chemistry *4*, 298-304.

Madrzak, J., Fiedler, M., Johnson, C.M., Ewan, R., Knebel, A., Bienz, M., and Chin, J.W. (2015). Ubiquitination of the Dishevelled DIX domain blocks its head-to-tail polymerization. Nature communications *6*, 6718.

Nguyen, D.P., Elliott, T., Holt, M., Muir, T.W., and Chin, J.W. (2011). Genetically encoded 1,2-aminothiols facilitate rapid and site-specific protein labeling via a bio-orthogonal cyanobenzothiazole condensation. Journal of the American Chemical Society *133*, 11418-11421.

Virdee, S., Ye, Y., Nguyen, D.P., Komander, D., and Chin, J.W. (2010). Engineered diubiquitin synthesis reveals Lys29-isopeptide specificity of an OTU deubiquitinase. Nature chemical biology *6*, 750-757.

Wang, M., Herrmann, C.J., Simonovic, M., Szklarczyk, D., and von Mering, C. (2015). Version 4.0 of PaxDb: Protein abundance data, integrated across model organisms, tissues, and cell-lines. Proteomics *15*, 3163-3168.

Wang, M., Weiss, M., Simonovic, M., Haertinger, G., Schrimpf, S.P., Hengartner, M.O., and von Mering, C. (2012). PaxDb, a database of protein abundance averages across all three domains of life. Molecular & cellular proteomics : MCP *11*, 492-500.
